# Phase II DeCOG-Study of Ipilimumab in Pretreated and Treatment-Naïve Patients with Metastatic Uveal Melanoma

**DOI:** 10.1371/journal.pone.0118564

**Published:** 2015-03-11

**Authors:** Lisa Zimmer, Julia Vaubel, Peter Mohr, Axel Hauschild, Jochen Utikal, Jan Simon, Claus Garbe, Rudolf Herbst, Alexander Enk, Eckhart Kämpgen, Elisabeth Livingstone, Leonie Bluhm, Rainer Rompel, Klaus G. Griewank, Michael Fluck, Bastian Schilling, Dirk Schadendorf

**Affiliations:** 1 Department of Dermatology, University Hospital, University Duisburg-Essen, Essen, Germany; 2 Department of Dermatology, Buxtehude, Germany; 3 University Department of Dermatology, Kiel, Germany; 4 Skin Cancer Unit, German Cancer Research Center (DKFZ), Heidelberg, Germany; 5 Department of Dermatology, Venereology and Allergology, University Medical Center Mannheim, Ruprecht-Karl University of Heidelberg, Mannheim, Germany; 6 University Department of Dermatology, Leipzig, Germany; 7 University Department of Dermatology, Tübingen, Germany; 8 Department of Dermatology, Erfurt, Germany; 9 Department of Dermatology, University Hospital, Ruprecht-Karl University of Heidelberg, Heidelberg, Germany; 10 Department of Dermatology, University Hospital, Erlangen, Germany; 11 Department of Dermatology, Kassel, Germany; 12 Department of Dermatology Hornheide, Münster, Germany; University Clinic of Navarra, SPAIN

## Abstract

**Purpose:**

Up to 50% of patients with uveal melanoma (UM) develop metastatic disease with limited treatment options. The immunomodulating agent ipilimumab has shown an overall survival (OS) benefit in patients with cutaneous metastatic melanoma in two phase III trials. As patients with UM were excluded in these studies, the Dermatologic Cooperative Oncology Group (DeCOG) conducted a phase II to assess the efficacy and safety of ipilimumab in patients with metastatic UM.

**Patients and Methods:**

We undertook a multicenter phase II study in patients with different subtypes of metastatic melanoma. Here we present data on patients with metastatic UM (pretreated and treatment-naïve) who received up to four cycles of ipilimumab administered at a dose of 3 mg/kg in 3 week intervals. Tumor assessments were conducted at baseline, weeks 12, 24, 36 and 48 according to RECIST 1.1 criteria. Adverse events (AEs), including immune-related AEs were graded according to National Cancer Institute Common Toxicity Criteria (CTC) v.4.0. Primary endpoint was the OS rate at 12 months.

**Results:**

Forty five pretreated (85%) and eight treatment-naïve (15%) patients received at least one dose of ipilimumab. 1-year and 2-year OS rates were 22% and 7%, respectively. Median OS was 6.8 months (95% CI 3.7–8.1), median progression-free survival 2.8 months (95% CI 2.5–2.9). The disease control rate at weeks 12 and 24 was 47% and 21%, respectively. Sixteen patients had stable disease (47%), none experienced partial or complete response. Treatment-related AEs were observed in 35 patients (66%), including 19 grade 3–4 events (36%). One drug-related death due to pancytopenia was observed.

**Conclusions:**

Ipilimumab has very limited clinical activity in patients with metastatic UM. Toxicity was manageable when treated as per protocol-specific guidelines.

**Trial Registration:**

ClinicalTrials.gov NCT01355120

## Introduction

Uveal melanoma (UM), arising from the iris, ciliary body, or choroid of the eye, represents 3% of all melanomas [1]. It is the most common primary intraocular malignant tumor in adults with an incidence of about 5 cases per million [1]. Up to 50% of patients develop metastatic disease, typically in the liver (89%) [2]. Prognosis at this stage is generally poor with a 1- and 2-year death rate of 80% and 92%, respectively [2]. UM is genetically distinct from cutaneous melanoma, with 80% to 90% of UMs showing activating mutations in *GNAQ* or *GNA11* [3,4] and lacking activating mutations in *BRAF*, *NRAS* and *TERT* promoter [5–7]. Treatment modalities for metastatic UM include most commonly systemic chemotherapy and hepatic intra-arterial chemoembolization [8,9]. However, the impact of these therapies on patients` survival is questionable [8,9]. To date, the improved understanding of the molecular biology of UM has not yet translated to successful treatment with targeted therapies [9], but clinical trials with protein kinase C (PKC) and MEK inhibitors (NCT01801358) [10–12] as well as other agents such as the multikinase inhibitor sorafenib (NCT01377025)[13], the c-Met/VEGFR2 inhibitor cabozantinib (NCT01835145) and the histone-deacetylase inhibitor vorinostat (NCT01587352) are in progress.

Apart from targeted therapies, agents modulating immunological checkpoints have shown great promise in the clinical management of patients with metastatic melanoma. Cytotoxic T-lymphocyte-associated antigen 4 (CTLA-4) is an immune checkpoint molecule that down-regulates T-cell activation, and its blockade by agonistic antibodies enhances antitumor immunity [14]. Ipilimumab, a fully human monoclonal antibody against CTLA-4, has shown an overall survival benefit in previously treated and treatment-naïve patients with metastatic melanoma in two randomized phase III trials [15,16]. As patients with metastatic UM had been excluded from these trials [15,16], the activity of ipilimumab in UM remains ill-defined. There is only one currently presented clinical phase II trial, which evaluated 10mg/kg ipilimumab in treatment-naïve patients with advanced UM [17]. Other published data are retrospective analyses of patients with UM who received treatment with ipilimumab under an expanded access program (EAP) or as a commercially available drug (S1 Table) [18–23].

We performed an open-label, multicenter, single-arm phase II clinical trial (DeCOG-trial) to further evaluate the efficacy and safety of 3mg/kg ipilimumab in treatment-naïve and pretreated patients with advanced UM seen in daily routine in interdisciplinary skin cancer units in Germany.

## Patients and Methods

The protocol for this trial (S1 Protocol and S2 Protocol) and supporting TREND checklist (S1 TREND Checklist) are available as supporting information.

### Patients

Eligibility criteria included documented unresectable stage III or stage IV metastatic ocular melanoma according to American Joint Committee on Cancer cutaneous melanoma staging criteria [24]. Pretreated and treatment-naïve patients were eligible. Previous systemic treatment had to be completed ≥ 28 days before receiving ipilimumab. Additional requirements included age ≥18 years, Eastern Cooperative Oncology Group (ECOG) performance status ≤2, life expectancy of ≥ 6 months (estimation of life expectancy was at the discretion of the participating investigators), measurable disease according to Response Evaluation Criteria In Solid Tumors (RECIST) 1.1 [25], adequate bone marrow, renal and hepatic function. Patients with a history of active autoimmune disease and chronic use of systemic corticosteroids were excluded. Patients with asymptomatic, radiographically stable previously treated or untreated brain metastases were eligible.

### Study Design

**Fig 1:**
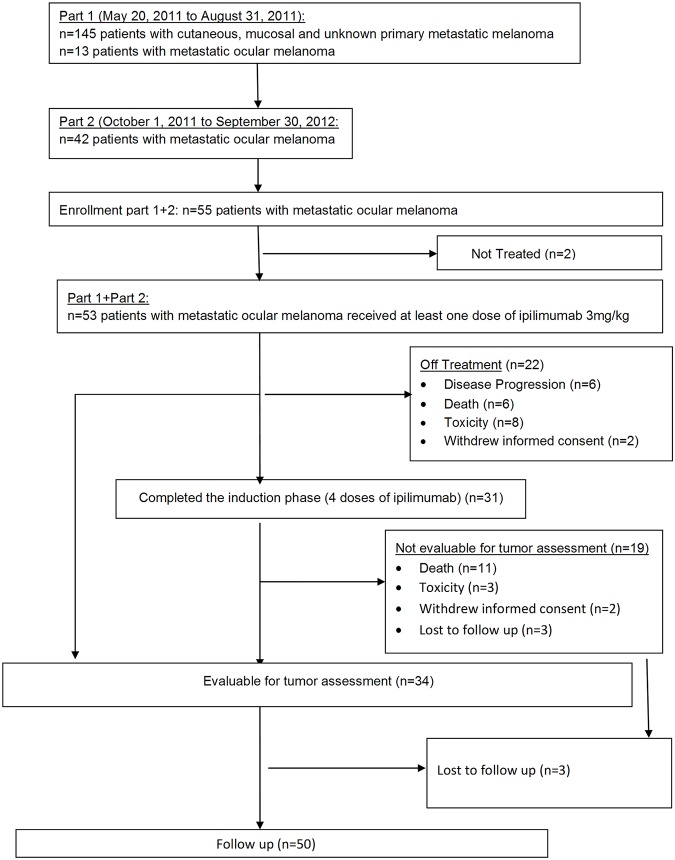
Consort Diagram for DeCOG-study.

This multicenter, open-label, phase II study (DeCOG-MM-PAL11-Trial; CA184–137) was conducted in two parts (Fig. 1). Part 1 of the study (Fig. 1), which allowed recruitment of pretreated melanoma patients irrespective of location of the primary melanoma, was closed on August 31, 2011 as the study had recruited sufficient numbers of patients with cutaneous melanoma. Part 2 was open for recruitment from October 2011 to September 2012. This part was only eligible for patients with pretreated or treatment-naïve metastatic ocular melanoma to allow for a valid analysis of this subgroup (Fig. 1). Thus, patients with ocular melanoma were included in part 1 and part 2 of the study (Fig. 1). Twenty five Dermatologic Cooperative Oncology Group (DeCOG) skin cancer units in Germany participated. The study was approved by institutional ethics committee University Duisburg-Essen (approval number 10–4531) and the German competent authority Paul-Ehrlich-Institut (Langen, Germany, approval number 1233), and conducted in accordance with the Declaration of Helsinki/Good Clinical Practice. All patients gave written informed consent. Ipilimumab was administered intravenously over 90 min at a dose of 3mg/kg every 3 weeks for a total of four infusions. Patients with progressive disease (PD) at ≥ 3 months from week 12 assessment following stable disease (SD), an initial partial (PR) or complete response (CR) were eligible for re-induction with ipilimumab following at the same dosage. Dose reduction was not allowed, but skipping of one dose of ipilimumab was recommended when adverse events (AE) occurred. Rapid disease progression, intolerable toxicity or patient withdrawal led to treatment discontinuation. The primary endpoint was the overall survival (OS) rate at 12 months.

### Assessments

Regular assessments, including a physical examination and standardized blood testing, were carried out at baseline and every 3 weeks during induction and re-induction phases. Tumor assessments were conducted at baseline, weeks 12, 24, 36 and 48 using the RECIST version 1.1 [25]. Adverse events (AEs) were graded according to the National Cancer Institute Common Toxicity Criteria (CTC version 4.0). All AEs were recorded from the time of the first ipilimumab administration until 70 days after treatment discontinuation. AEs were defined as an immune-related AE (irAE) if they were associated with drug exposure, consistent with an immune phenomenon and if other causes were ruled out. IrAE management was based on protocol-specific treatment algorithms. All AEs that were definitely, probably or possibly related to study drug were defined as related AEs.

### Statistical Methods

This report includes results based on the data cutoff of December 6, 2013. Since advanced ocular melanoma is a rare disease, all patients being available in the study period were included into the study to get as much information as possible. Patient and disease characteristics were analyzed using descriptive statistics. Categorical values were expressed as counts and percentage whereas continuous values were expressed as median and range values. OS was defined as the time from the first administration of ipilimumab to death from any cause. Patients last known to be alive were censored at the date of last contact. Progression-free survival (PFS) was defined as the time from the first dose of ipilimumab to the first date of documented progression as per RECIST, or date of death, whichever came first. Patients last known to be alive and progression-free were censored at the date of last contact. PFS rate at 6 months was defined as the proportion of patients being alive and without progress 6 months after the first ipilimumab administration. Patients with unknown survival status or unknown status of progression at 6 months were censored. The one- and two-year survival rates were defined as the proportion of patients being alive 12 or 24 months after their first ipilimumab administration. Patients with unknown survival status at 12 or 24 months were censored. OS, PFS, PFS rate at 6 months, one- and two-year survival rates were estimated by the Kaplan-Meier method. For medians of OS and PFS, 95% confidence intervals (CIs) were calculated using the Brookmeyer and Crowley method. The log-rank test was used to compare the one-year and two-year OS rates between several subgroups, i.e. the presence of brain metastases, the number of prior therapies (0 vs. ≥ 1), the lactate dehydrogenase (LDH) level prior to receiving ipilimumab (<2-fold upper level norm (ULN) vs.≥ 2xULN), the number of ipilimumab doses (<4 vs. 4), and the absolute lymphocyte count (ALC) (<1000/μl vs. ≥1000/μl) before the first (week 1), the second (week 4) and the third dose (week 7) of ipilimumab. Two sided p-values were evaluated and a p-value of <0.05 was considered statistically significant. All variables with significant differences between their stratifications regarding the overall survival were included in a multivariate Cox proportional hazards model. To determine potential predictors, all independent covariates (LDH, number of ipilimumab doses, ALC week 7), were entered into a backward Cox regression model for the overall survival. The stay level was p = 0.05. All covariates being still significant were considered as potential predictors. For the hazard ratio, 95% CIs were calculated using the Wald method. The overall response rate (ORR) was defined as the proportion of patients with PR and CR whereas the disease control rate (DCR) was defined as the proportion of patients with CR, PR and SD. Lost to follow-up was documented if the patient did not respond to phone calls (3 times) and to a written invitation. Analyses were carried out using SAS software, version 9.3 (Cary, NC).

## Results

### Patients

Between May 2011 to September 2012, 53 patients with metastatic UM were enrolled and received at least one dose of ipilimumab (Table 1). Baseline patient characteristics are reported in table 1. All 53 patients had distant metastases with lung, liver and/or brain involvement. Forty five patients (85%) had received at least one previous systemic anti-cancer treatment, including chemotherapy and targeted agents, and eight patients were treatment-naive (15%) (Table 1). Thirty one patients (58%) completed the induction phase, none were re-induced. The median number of doses received in the induction phase was four (range 1–4). Among the 22 patients (42%) who did not complete the induction phase, six (11%) died, six (11%) developed PD, eight (15%) had intolerable AEs and two (4%) withdrew their informed consent.

**Table 1:** Patients characteristics.

Patient characteristics		Ocular Melanoma	
		N	%
**Number of patients**		53	100
**Sex**	Male	23	43
	Female	30	57
**Age, years**	Median (range)	67 (34–84)	
**ECOG baseline**	0	38	72
	1	14	26
	2	1	2
**Patients with only lung metastases**		2	4
**Patients with liver metastases**		51	96
**Brain metastases**	No	50	94
	yes	3	6
**LDH**	<2 upper level norm	33	62
	> = 2 upper level norm	20	38
**Prior systemic therapy in**	No	8	15
stage IV (except	Yes	45	85
radiotherapy)	1	28	53
	2	9	17
	≥3	4	7.5
	Not known	4	7.5
**Immunotherapy**	No	53	100
**Small molecules**	No	46	87
	Yes	7	13
	Sorafenib	7	13
**Chemotherapy**	0	16	30
	1	25	47
	2	9	17
	≥3	3	6

Abbreviations: ECOG, Eastern Cooperative Oncology Group; LDH, lactate dehydrogenase

### Efficacy, Overall survival

**Fig 2:**
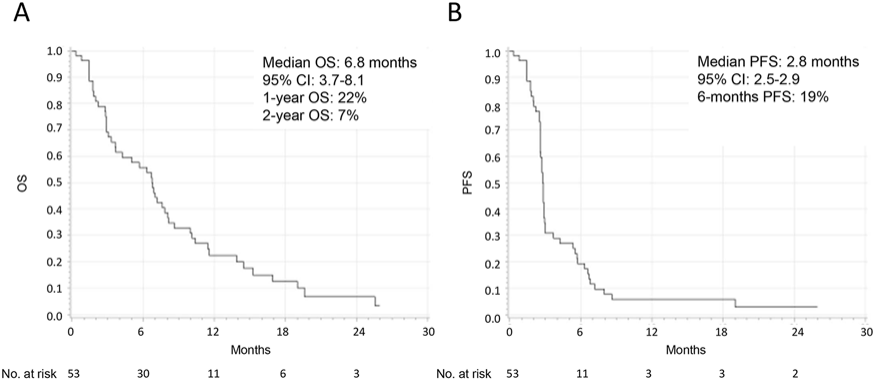
Kaplan-Meier curves for overall survival (A) and progression-free survival (B) of treatment-naïve and pretreated patients with metastatic ocular melanoma who received ipilimumab 3 mg/kg.

1-year, 2-year rates for OS and 6-month rate for PFS were 22% (95% CI 12–35), 7% (95% CI 1–18) and 19% (95% CI 10–31), respectively. Median OS and PFS from the first dose of ipilimumab were 6.8 (95% CI 3.7–8.1; Fig. 2A) and 2.8 (95% CI 2.5–2.9; Fig. 2B) months, respectively. Thirty four of 53 patients were evaluable for efficacy assessment (Table 2). Among the 19 patients (36%) who were not assessable, 11 died before the assessment of change in tumor burden, three had severe AEs, two withdrew their informed consent and three were lost to follow-up. The DCR was 47% at week 12 and 21% at week 24 (Table 2). No partial or complete response was observed. Eight patients died between week 33 and week 86 after start of ipilimumab treatment, where no tumor assessment at week 24 had been performed. The reason for not performing tumor assessment at week 24 was unknown.

**Table 2:** Best responses to treatment.

Overall response rate/Disease control rate	Ocular Melanoma	
	N	%
**Number of patients**	53	100
**Number of patients with measurable disease and at least one tumor assessment**	34	100
**Disease control rate (CR or PR or SD)** ^**a**^		
Week 12	16	47
Week 24	7	21
**Overall response rate (PR+CR) (RECIST)**	0	0
Stable disease	16	47
Progressive disease	18	53

^a^Disease control rate refers to total number of patients with measurable disease and at least one tumor assessment (n = 34).

**Fig 3:**
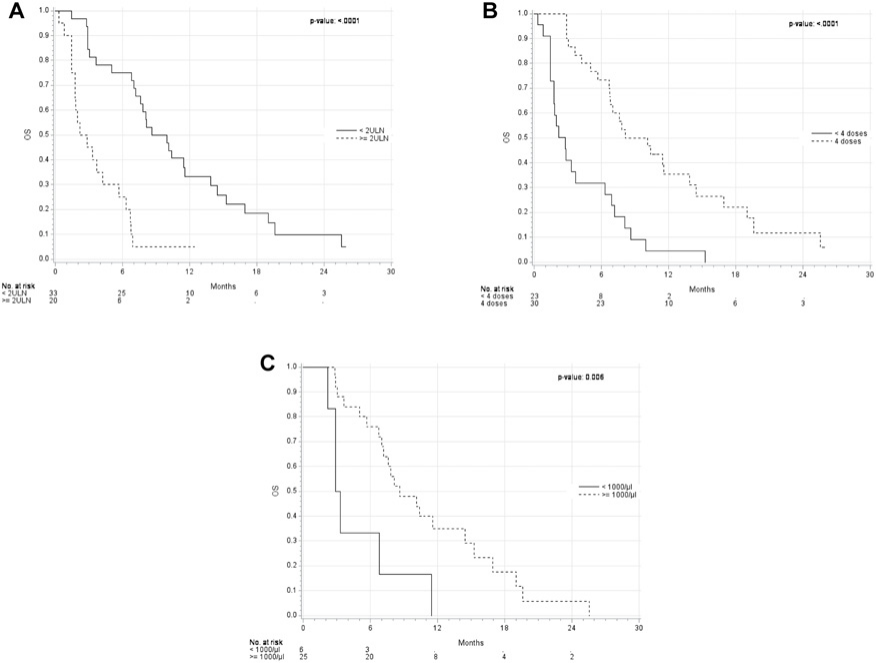
Kaplan-Meier curves for overall survival stratified by the lactate dehydrogenase (LDH) level before the first dose of ipilimumab (A), the number of ipilimumab doses (B), and the absolute lymphocyte count (ALC) before the third dose (week 7) of ipilimumab (C). (A) LDH level (<2-fold upper level norm (ULN) versus ≥ 2xULN). LDH < 2xULN: median OS: 9.3 months (95% CI 7.0–11.6); LDH ≥ 2xULN: median OS 2.5 months (95% CI 1.5–5.7). (B) the number of ipilimumab doses (<4 versus 4). 4 doses: median OS: 9.1 months (95% CI 6.7–13.9); < 4 doses: median OS: 2.8 monhts (95% CI 1.5–6.3). (C) ALC (<1000/μl versus ≥1000/μl). ALC ≥1000/μl: median OS: 8.6 (95% CI 7.0–14.5); ALC <1000/μl: median OS: 3.1 (95% CI 2.2–11.5).

The 1-year OS rate was higher in patients with a LDH level < 2xULN (33% vs. 5%, p<0.0001; Fig. 3A), in patients who received four ipilimumab doses (36% vs. 5%, p<0.0001; Fig. 3B), and in patients with an ALC ≥ 1000/μl before the third dose of ipilimumab (week 7) (35% vs. 0%, p<0.006; Fig. 3C). The apparent better OS observed in patients who received all four ipilimumab doses, could be solely based on a time dependent bias, as receiving four doses of ipilimumab required surviving >10 weeks after therapy initiation. This only applied to half of the patients who received <4, but not surprisingly all with four doses of ipilimumab. Presence of brain metastases, no prior therapy and ALC before the first and the second dose of ipilimumab were not associated with OS. In a multivariate analysis, the only factor independently associated with better OS was an ALC ≥ 1000/μl before the third dose of ipilimumab (week 7), e.g. patients with ALC < 1000/μl were at higher risk of death than patients with an ALC ≥ 1000/μl (hazard ratio 3.6; 95% CI 1.4–9.4).

### Safety

Fifty one patients (96%) experienced one or more AEs (Table 3). Treatment-related AEs were reported in 35 patients (66%); 19 patients (36%) had treatment-related grade 3 or 4 AEs. The majority of treatment-related AEs were irAEs, occurring in 32 patients (60%). Most common irAEs were gastrointestinal disorders—diarrhea and colitis, skin-related toxic effects—pruritus and rash, and hepatic disorders—increased alanine aminotransferases (ALT) and aspartate aminotransferases (Table 3). The most frequent grade 3 or 4 irAEs were diarrhea and colitis, noted in seven (13%) and six patients (11%), respectively. Immune-related AEs were generally reversible when managed as per protocol-specific treatment guidelines. Most of the irAEs resolved with corticosteroid therapy. Three of six patients with grade 3 or 4 colitis required additional immunosuppression with anti-TNF-alpha (infliximab), and one patient with increased liver enzymes was treated with mycophenolate mofetil. The addition of infliximab and mycophenolate mofetil led to a complete remission of the latter irAEs. Non-irAEs included arthralgia, fatigue, nausea, fever and influenza-like symptoms. There was one possible treatment-related death due to pancytopenia with following cerebral hemorrhage and respiratory insufficiency.

**Table 3:** Adverse events.

Adverse Events (AE)^a^	Ocular melanoma	
	Number of Patients (%) (n = 53)	
	All grades	Grade 3/4
**Patients with at least one AE**	51 (96)	32 (60)
**Treatment-related AE**	35 (66)	19 (36)
**Any irAE**	32 (60)	16 (30)
**irDermatitis**	11 (21)	0
Pruritus	5 (9)	0
Rash	3 (6)	0
Erythema multiforme	3 (6)	0
**irGastrointestinal disorders**	25 (47)	13 (24)
Colitis	9 (17)	6 (11)
Diarrhea	16 (30)	7 (13)
**irEndocrine disorders**	1 (2)	0
Hypophysitis	0	0
Hypothyroidism	0	0
Hyperthyroidism	1 (2)	0
**irHepatic disorders**	7 (13)	4 (8)
Increased ALT	4 (7)	2 (4)
Increased AST	3 (6)	2 (4)

Abbreviations: ir, immune related; ALT, alanine aminotransferases; AST, aspartate aminotransferases.

^a^Patients may have had more than one adverse event.

## Discussion

This is the first phase II trial evaluating the efficacy and safety of 3mg/kg ipilimumab in a large cohort of 45 pretreated and 8 treament-naïve patients with metastatic UM in a prospective fashion. Apart from our study there is only one further clinical phase II study evaluating 10 mg/kg ipilimumab in 32 treatment-naïve patients with UM [17]. All other published data are retrospective analyses of patients with metastatic UM who received treatment with ipilimumab under an EAP or as a commercially available drug [18–23] (S1 Table). Our findings show that ipilimumab has very limited clinical activity in patients with metastatic UM. In this study, patients without prior therapy did not demonstrate an improved one-year or two-year OS rate compared to pretreated patients. The overall safety profile was consistent with previous clinical data [15].

There are several limitations to interpreting data from this phase II trial; 1) the single-arm, non-randomized phase II design, however, no clear standard therapy for metastatic UM exists, 2) the lack of central review of imaging studies, 3) the missing classification of tumor assessments according to immune-related response criteria [26], 4) eleven patients died before the first assessment of change in tumor burden and thus, retrospectively, did not meet the inclusion criteria “life expectancy of ≥6 months”. Life expectancy, however, can always only be estimated and the rapid death of 11 patients reflects the poor prognosis of patients with metastatic UM for whom median time from diagnosis of metastasis to death of less than 6 months has been reported [2], 5) the sample sizes of multivariate subgroup analyses were small and its value therefore limited, however, this is a common problem for all studies involving patients with a rare tumor such as UM [22].

The 1-year OS rate of 22% and the median OS of 6.8 months in our study are consistent with two previous retrospective analyses of patients with UM who received treatment with ipilimumab in the Dutch- and Italian EAP [18,20] (S1 Table). The 1-year OS rate and the median OS for patients in the Dutch EAP were 27% and 5.2 months, respectively [20] and in the Italian EAP calculated 31% and 6 months, respectively [18]. In contrast to the 3 European studies from Germany, Italy and Netherlands who all used 3 mg/kg ipilimumab, the median OS was between 9 and 10 months for patients with UM who were treated with ipilimumab in further four retrospective studies [19,21–23]. Besides the study from Deo et al. [23] with a reported 1-year OS rate of 46%, none of these retrospective studies reported a 1-year OS rate (S1 Table). Apart from our trial, there is only one further clinical phase II study of the Spanish Melanoma Group (GEM) evaluating the efficacy of 10 mg/kg ipilimumab in 32 patients with treatment-naïve metastatic UM [17]. In this trial the median OS was 9.8 months, the 1-year OS rate was not reported. The irDCR was close to 50%. Due to the different ipilimumab dose of 10mg/kg and the inclusion of only treatment-naïve patients with metastatic UM, comparing results of this trial [17] with the results of our trial and previous studies [18,20] is difficult (S1 Table). Treatment of UM patients with 10mg/kg ipilimumab may have driven selection to fitter patients with a better upfront prognosis. Regarding other treatment modalities for metastatic UM, e.g. chemotherapy, 1-year OS rates range between 32% to 60% [10,13,27,28] and median OS between 9 to14 months [10,13,27–29] (S1 Table). For patients treated with the MEK inhibitor selumetinib the 1-year OS rate and the median OS were 42% and 11.8 months, respectively [10]. The 1-year OS rate after diagnosis of metastasis in the largest published series of unselected patients with UM was 20% with a median OS of less than 6 months [2]. At the time of diagnosis of metastasis, 397 (61%) out of 650 had no melanoma specific treatment, i.e. best supportive care, compared with 253 patients (39%) who had specific treatment, i.e. chemotherapy, radiation, immunotherapy, alone or in combination. In this trial, there were no differences in OS after diagnosis of metastasis between patients receiving treatment compared with those not receiving treatment of metastasis [2]. When comparing results of different studies, it has to be kept in mind that the trial design, the patient numbers and patient selection criteria influences OS. To summarize, regardless of trial differences, the results of our prospective phase II trial show that ipilimumab has very limited activity in patients with metastatic UM.

Given the side effects [15,16] and treatment costs [30] of ipilimumab as well as the poor prognosis of advanced UM with rapid clinical deterioration [2], early biomarkers of response would be very helpful. In our study, an ALC ≥ 1000/μl at week 7 was the only factor independently associated with a better OS. This is in line with the results of previous studies, which reported that the rise in ALC during ipilimumab treatment at week 4 or week 7 appears to correlate with OS [22,31,32]. However, the sample size of patients with an ALC < 1000/μl in our study was small and these results therefore need further confirmation in a large prospective trial. A large prospective phase I/II trial on ipilimumab in patients with locally treated UM (adjuvant arm) and metastatic UM (metastatic arm) is still recruiting patients and will not be finished before 2017 (NCT01585194). Besides CTLA-4 blockade by ipilimumab, targeting new immune checkpoint inhibitory receptors and ligands such as programmed cell death 1 (PD-1) and programmed cell death ligand 1 (PD-L1) have evolved as further important targets in cutaneous melanoma [33–38]. PD-L1 expression is found in primary and metastatic UM [39] and treatment strategies targeting PD-1/PD-L1 may also be of interest.

The understanding of the molecular biology of UM has led to more recent phase I/II studies investigating different kinase inhibitors as single agent [12], in combination with chemotherapy [10] or in combination with a PKC inhibitor (NCT01801358). In a randomized phase II study comparing the MEK inhibitor selumetinib with chemotherapy (temozolamide or dacarbazine) in patients with metastatic UM, the median PFS was significantly improved from 7 weeks with chemotherapy to 15.9 weeks with selumetinib. However, there was no improvement in OS. In a phase I study of the MEK inhibitor trametinib, 8 of 16 patients with UM experienced SD [12]. However, PFS was limited to only 1.8 months [12]. Recently, the PKC inhibitor, AEB071 has shown preliminary activity in metastatic UM with 72 out of 141 patients having PR/SD and a median PFS of 15 weeks [11]. A phase I/II study evaluating the combination of the MEK inhibitor MEK162 and the PKC inhibitor AEB071 in metastatic UM is currently recruiting patients (NCT01801358).

Similar to previous studies of ipilimumab at a dose of 3 mg/kg [15,40] immune-related dermatological AEs, i.e. pruritus and rash, and immune-related gastrointestinal AEs, i.e. diarrhea and colitis were the most frequent treatment-related adverse events. The rate of grade 3 and 4 treatment-related AEs in our study was higher compared with a previous phase III study of ipilimumab [15]. Immune-related gastrointestinal and hepatic AEs were the most common grade 3 and 4 irAEs. Consistent with our findings, grade 3 and 4 hepatic disorders were the most frequent irAEs in patients with metastatic UM treated in the Italian EAP [18]. Most of the irAEs were reversible when managed as per protocol-specific treatment guidelines and resolved with systemic glucocorticosteroid therapy. There was one possible treatment-related death due to pancytopenia. Although attributed to ipilimumab, it is possible that the concomitant medication of the patient caused the pancytopenia.

In conclusion, ipilimumab has very limited clinical activity in patients with metastatic UM. Due to the low number of patients in the treatment-naïve group, further confirmation of our findings in a randomized controlled trial evaluating the efficacy of ipilimumab in treatment-naïve patients with metastatic UM versus pretreated patients is required. The ALC at week 7 appears to be an early biomarker of response and should be investigated in prospective clinical trials. Immune-related AEs were manageable and reversible in most of the cases. Intensive biomarker research and patient characterization are necessary to help determine the optimal patient subgroup and treatment time point for ipilimumab and other novel immune checkpoint inhibitors in patients with UM in both the adjuvant and first line metastatic settings.

## Supporting Information

S1 Protocol DeCOG trail protocol part 1. (PDF)

S2 Protocol DeCOG trail protocol part 2. (PDF)

S1 Table Summary of previous studies reporting efficacy of ipilimumab and other therapies in metastatic uveal melanoma within the last 5 years. (PDF)

S1 TREND Checklist (PDF)
