# Supplementary material for: Phase II DeCOG-Study of Ipilimumab in Pretreated and Treatment-Naïve Patients with Metastatic Uveal Melanoma
Source: PLoS One. 2015 Mar 11;10(3):e0118564. doi: 10.1371/journal.pone.0118564 (PMC4356548; doi:10.1371/journal.pone.0118564)
Supplement: S1 Protocol — (PDF) [file pone.0118564.s001.pdf]

Clinical Trial Protocol DeCOG –MM-PAL11

THE IPI – MULTIBASKET TRIAL IN ADVANCED MELANOMA:

PROSPECTIVE CLINICAL PHASE II MULTIBASKET STUDY IN MELANOMA PATIENTS WITH ADVANCED DISEASE

Sponsor's Protocol Code Number	DeCOG –MM-PAL11
EUDRACT number	2010-021946-22
Version number / Protocol date	1.0 (07.April 2011)
Development phase	II
Document status	Final

Sponsor: University Hospital Essen
Hufelandstr. 55
D- 45122 Essen
Germany
Represented by Prof. Dr. Dirk Schadendorf

Coordinating Investigator: Prof. Dr. Dirk Schadendorf
Department of Dermatology, Skin Cancer Center
Comprehensive Cancer Center Essen
Hufelandstr. 55
D- 45122 Essen,
Germany
Telephone Number: +49 (0) 201-723 4342
Fax Number: +49 (0) 201-723 5935
e-mail: dirk.schadendorf@uk-essen.de

Clinical Research Organisation: Alcedis GmbH
Winchesterstraße 2
D- 35394 Gießen (Europaviertel)
Germany
Telephone Number +49 (0) 641/ 94436-0
Fax Number: +49 (0) 641/ 94436-70

This protocol is confidential information and is intended solely for the guidance of the clinical investigation. This protocol may not be disclosed to third parties not associated with the clinical investigation or used for any purpose without the prior written consent of the sponsor.

Signature Sheet

Sponsor:

University Hospital Essen
Hufelandstr. 55
D- 45122 Essen
Germany
Represented by Prof. Dr. Dirk Schadendorf

08/04/2011
Date

Signature

Coordinating Investigator:

Prof. Dr. Dirk Schadendorf
Department of Dermatology, Skin Cancer Center
Comprehensive Cancer Center Essen
Hufelandstr. 55
D- 45122 Essen
Germany
Telephone Number: +49 (0) 201-723 4342
Fax Number: +49 (0) 201-723 5935
e-mail: dirk.schadendorf@uk-essen.de

08/04/2011
Date

Signature

Statistical Processing:

Alcedis GmbH
Dr. Christine Windemuth-Kieslbach
Winchesterstraße 2
D- 35394 Gießen (Europaviertel), Germany

08/04/2011
Date

Signature

Clinical Research Organisation:

Alcedis GmbH
Dr. Elke Heidrich-Lorsbach
Winchesterstraße 2
D- 35394 Gießen (Europaviertel), Germany

08/04/2011
Date

Signature

Gelöscht: 08 March 2011

Signature Page for Investigators

**Sponsor's Protocol Code Number**	DeCOG –MM-PAL11
**EudraCT-No.**	2010-021946-22
**Title**	Prospective Clinical Phase II Multibasket Study in Melanoma Patients with advanced Disease
**Version**	1.0
**Date of protocol**	07.April 2011

I have read this protocol and agree that it contains all necessary details for carrying out this study. I will conduct the study as outlined herein and will complete the study within the time designated, in accordance with all stipulations of the protocol and in accordance with Good Clinical Practices, local regulatory requirements, and the Declaration of Helsinki. I will provide copies of the protocol and all pertinent information to all individuals responsible to me who assist in the conduct of this study. I will discuss this material with them to ensure that they are fully informed regarding the study drug and the conduct of the study.

I will use only the informed consent forms approved by the Institutional Review Board/Independent Ethics Committee (IRB/IEC) and will fulfill all responsibilities for submitting pertinent information to the IRB/IEC responsible for this study.

I agree that the Sponsor (University Hospital Essen) or its representatives shall have access to any source documents from which case report form information may have been generated.

Center Number

Investigator's name

Institution

Address

______________________________ __________________________
Signature Investigator Date

TABLE OF CONTENTS

PROTOCOL SYNOPSIS 9
1 INTRODUCTION 18
1.1 Clinical Experience and Research Hypothesis 18
1.2 Product Development Rationale 18
1.2.1 CTLA-4 and T Cell Activation 19
1.3 Summary of Results of Investigational Program 21
1.3.1 Pharmacology of Ipilimumab 21
1.3.2 Pre-Clinical Toxicology of Ipilimumab 21
1.3.3 Human Pharmacokinetics of Ipilimumab 22
1.3.4 Clinical Safety with Ipilimumab 22
1.3.5 Immune-Related Adverse Events (irAEs) with Ipilimumab 24
1.3.6 Clinical Efficacy of Ipilimumab 29
1.3.7 Rationale for using immune-related Tumor Assessment Criteria (irRC) 32
1.4 Overall Risk/Benefit Assessment 33
1.5 Study Rationale 34
2 STUDY OBJECTIVES 37
2.1 Primary Objective 37
2.2 Secondary Objectives 37
2.3 Exploratory Objectives 37
3 STUDY DESIGN 38
3.1 Overall Study Design 38
3.2 Enrollment of Patients 40
3.3 Number of Patients 40
3.4 Timelines 40
3.5 Definition of the End of Study 40
3.6 Plan for Treatment after the End of Study 40

4 SUBJECT SELECTION CRITERIA 41
4.1 Inclusion Criteria 41
4.2 Exclusion Criteria 42
4.3 Justification of Gender 43
5 STUDY THERAPY WITH IPILIMUMAB 44
5.1 Induction Treatment 44
5.2 Re-Induction Treatment 45
5.3 Definition of Selected Reversible irAEs that May be Considered For Re-Treatment 46
5.4 Dose Calculations 47
5.5 Preparation, Administration, Dose Modification, Discontinuation 47
5.5.1 Preparation and Administration Guidelines 47
5.5.2 Dose Modifications 48
5.5.3 Discontinuation of Study Therapy 50
5.5.4 Immune-Related Adverse Events (irAEs): Definition, Monitoring, and Treatment 51
5.5.5 Other Guidance 52
5.6 Prohibited and Restricted Therapies During the Study 54
5.6.1 Prohibited Therapies 54
5.6.2 Restricted Therapies 54
6. STUDY PROCEDURES AND OBSERVATIONS 55
6.1. Procedures by Visit 55
6.1.1. Study Procedures by Visit and Treatment Cycle 55
6.2. Safety Assessments 61
6.3. Stopping rules 61
6.4. Criteria for Evaluation 62
6.4.1. Safety Evaluation 62
6.4.2. Efficacy Evaluation 62

6.4.3. Evaluation of Tumor Response Using RECIST Criteria 63
6.4.4. Definition of Tumor Response Using irRC 65
6.4.5. Response Endpoints 67
6.5. Translational Research Programm 68
6.5.1. Soluble Factors in Patients´ Serum 68
6.5.2. Microtissue Array 69
6.5.3. Storage and Confidentiality of Specimens 69
7. INVESTIGATIONAL PRODUCT: IPILIMUMAB 69
7.1. Identification 70
7.2. Packaging and Labeling 70
7.3. Storage, Handling, and Dispensing 70
7.3.1. Storage 70
7.3.2. Handling and Disposal 70
7.3.3. Dispensing 70
7.4. Drug Ordering and Accountability 71
7.4.1. Initial Orders 71
7.4.2. Re-Supply 71
7.5. Ipilimumab Accountability 71
7.6. Ipilimumab Destruction 72
8. ADVERSE EVENT REPORTING 72
8.1. Collection of Safety Information 72
8.1.1. Serious Adverse Events 72
8.1.2. Nonserious Adverse Events 73
8.2. Assignment of Adverse Event Intensity and Relationship to Investigational Product 73
8.3. Collection and Reporting 74
8.3.1. Serious Adverse Events 74
8.3.2. Handling of Expedited Safety Reports 75
8.3.3. Non-serious Adverse Events 76
8.3.4. Pregnancy 76

8.3.5. Other Safety Considerations 77
9. STATISTICAL METHODOLOGY 78
9.1. Sample Size Justification 78
9.2. Data collection 78
9.3. Endpoints 79
9.3.1. Primary Efficacy Parameter 79
9.3.2. Secondary Efficacy Parameters 79
9.4. Study population 80
9.5. Statistical analysis 80
9.6. Statistical Software 81
10. DATA MANAGEMENT 82
10.1. Patient Identification 82
10.2. Data Collection 82
10.2.1. Employed Software 82
10.2.2. Data Security and Storage 83
11. MONITORING 84
11.1. Monitoring 84
11.2. Audit, Inspections 84
12.1. Coordinating Investigator in Accordance with Section 40 AMG 85
12.2. Compliance with the Protocol and Protocol Revisions 85
12.3. Good Clinical Practice 86
12.4. Study Monitoring 86
12.5. Maintenance of a Study File 86
12.6. Authorisation of Competent Authority 86
12.7. Ethics Committee Approval 87
12.8. Notification to the Competent Supervisory Authorities87
12.9. Archival 87

12.10. Confidentiality 88
12.11. Patient Insurance 88
12.12. Final Report 88
12.13. Third Party Financing 88
13. ETHICAL CONSIDARATIONS 88
13.1. Declaration of Helsinki 88
13.2. Patient Information 89
13.3. Informed Consent 89
13.4. Use, Storage and Transmission of Data 89
Appendix 1: List of Abbreviations 90
Appendix 2: AJCC 2010- Melanoma TNM Classification 92
Appendix 3: Participating Trial Centers 94
Appendix 4: ECOG Performance Status 95
Appendix 5: Declaration of Helsinki 96
Appendix 6: Common Toxicity Criteria (CTCAE) Scale 100
Appendix 7: Patient Information and Informed Consent 101
Appendix 8: irAE Treatment Algorithm 102
Appendix 9: RECIST Criteria 103
Appendix 10: Management Algorithm for AEs 105
REFERENCES 106

PROTOCOL SYNOPSIS

**Protocol Title:**<br>**Prospective Clinical Phase II Multibasket Study in Melanoma Patients with advanced Disease**
**Sponsor:**<br>University Hospital Essen<br>Hufelandstr. 55<br>D- 45122 Essen<br>Represented by Prof. Dr. Dirk Schadendorf
**Coordinating Investigator:**<br>Prof. Dr. Dirk Schadendorf<br>Department of Dermatology, Skin Cancer Center<br>Comprehensive Cancer Center Essen<br>Hufelandstr. 55<br>D- 45122 Essen,<br>Germany<br>Telephone Number: +49 (0) 201-723 4342<br>Fax Number: +49 (0) 201-723 5935<br>e-mail: dirk.schadendorf@uk-essen.de
**Study Design:**<br>This is an open-label, multi-center, single-arm clinical phase II study to further characterize the efficacy and safety of ipilimumab in patients with pretreated, metastatic and/or inoperable melanoma.
**Planned Study Period:**<br>Period of recruiting 8-9 months<br>Enrolment start date (FPI): QII 2011<br>Enrolment finish date (LPI): December 2011<br>End of study: QIV 2012
**Planned Total Number of Patients:**<br>Up to 200 patients
**Country(ies) and No. of Sites:**<br>Germany – up to 26 sites
**Study Rationale**<br>Various phase II clinical studies using ipilimumab in unresectable stage III-IV have been conducted. Results of the first pivotal phase III study MDX010-20 have been presented at ASCO 2010 (O´Day et al., JCO) and are published in NEJM (Hodi et al., NEJM, 2010). In the second ongoing phase III trial CA184-024, a different dose and treatment scheme is currently tested. Results are expected at the end of 2010.<br>However, due to strictly defined inclusion and exclusion criteria in phase III trials, results of

registrational trials might not answer all questions regarding safety and efficacy of ipilimumab in all advanced melanoma patient cohorts seen in daily routine. E.g. on one hand, in MDX010-20 trial, only patients positive for HLA-A2 could be enrolled (since patients were randomized in a double-blind fashion in a 1:3:1 ratio to receive HLA-A2-restricted melanoma gp 100 peptide vaccine monotherapy, MDX-010 in combination with melanoma gp100 peptide vaccine, or MDX-010 monotherapy.) In the second currently ongoing phase III study CA184-024 patients were not restricted to HLA-A2+ haplotype. However, another dosing and treatment scheme was tested (combination with DTIC, ipilimumab 10mg/kg and maintenance period following induction phase for patients who benefitted from ipilimumab) for untreated advanced malignant melanoma patients. Patients with primary mucosal melanoma were excluded in CA184-024 study; patients with primary ocular melanoma were excluded in both MDX-010-20 as well as in CA184-024.
The currently described study concept with ipilimumbab 3mg/kg would make a rational therapeutic strategy in a broad range of pretreated metastatic melanoma patients seen in daily clinical practice.

Research Hypothesis

A large clinical phase II is proposed to treat a broad range of metastatic melanoma patients who have failed at least one systemic treatment in unresectable stage III-IV melanoma in order to further characterize efficacy and safety of ipilimumab in a broad range of clinically relevant patient cohorts seen in daily routine, neither addressed in MDX-010-20 study nor in CA184-024. Enrollment will not be restricted to any specific HLA subtype and patients with uveal or ocular melanoma can be treated with ipilimumab as well. Wide access to ipilimumab in skin cancer-specialized clinical centers will be achieved in addition to a standardized evaluation of its clinical results and a further evaluation of algorithms for the management of side effects. Patients will be treated according to the MDX-010-20 protocol with ipilimumab monotherapy 3mg/kg four times during the induction phase.

Treatment:

Treatment with the anti-CTLA-4 mAb Ipilimumab monotherapy of each patient in the scope of this trial is defined as induction plus re-induction of eligible patients until 12 months after first receipt of study medication

Induction phase:
Ipilimumab will be applied to melanoma patients according to the protocol of the completed Medarex study MDX-010-20: Ipilimumab by IV infusion, 3 mg/kg, day 1 (W1), 22 (W4), 43 (W7), 64 (W10)

Re-induction:

Patients who progress following stable disease of ≥ 3 months duration starting from diagnosis at week 12 tumor assessment or patients who have progressed following an initial response (partial or complete) assessed at week 12 may be offered additional cycles of therapy with the originally assigned treatment regimen until off-treatment criteria are met, provided they meet re-treatment eligibility requirements. Please note that re-induction is allowed only if progressive disease was confirmed within not less than 4 weeks. No patient will be re-treated if they experience a Grade 3 or higher gastrointestinal or certain other immune-related adverse events (irAE) (refer to section 5.2 and 5.3). No patient with disease progression following the first cycle of study medication will be permitted to be re-treated with study medication.

Examinations:

The disease will be assessed at baseline, at week 12 and for patients with stable disease or better responses, thereafter every 12 weeks in the absence of PD with a maximum of one year after receive of the first treatment. Response evaluation will be done according to immune-related response criteria (Wolchok et al., CCR 2009).

All patients who prematurely discontinued treatment due to a drug-related adverse event prior to Week 12 (in the absence of disease progression) will return for all study visits and procedures including Week 12 and, if appropriate, further re-staging assessments. Any patient with documented progression at any scheduled re-staging visit and who will not receive any re-induction will undergo no further re-staging visits.

Follow-up phase:

Survival will be assessed every 3 months after the final dose of Ipilimumab until the end of the follow-up phase for the individual patient. FU phase for each subject is 1 year following first treatment dose. End of study will be at recruitment finished plus 1 year post start of treatment of last patient thus ensuring that 1 year survival rate can be estimated.

Study duration:

End of study is 1 year post LPFV. Therefore expected maximum study duration is 8-9 months (Period of recruiting) accrual + 12 months for follow-up.

Primary Objective

- To further characterize efficacy of second line ipilimumab monotherapy 3mg/kg given according to the MDX010-20 protocol in a broad range of pretreated metastatic melanoma patients seen in daily clinical practice

Primary Endpoint

- Overall survival rate at 12 months defined as the rate of patients alive 12 months after the date from the first study treatment for complete study population

Secondary Objective(s)

To explore safety and additional efficacy parameters of the ipilimumab treatment in certain subgroups: (cutaneous versus uveal versus mucosal melanoma; LDH < 2 ULN versus LDH ≥ 2 ULN; status brain metastases versus no brain metastases in medical history; b-raf mutation positive versus b-raf mutation negative; HLA-A2 positive versus HLA-A2 negative)

Secondary objectives are

- Safety / toxicity according to the CTC Criteria (Version 4.0)
- Efficacy according to immune-related response criteria (ir-RC) at any time during treatment
- Efficacy according RECIST criteria
- Progression free survival rate at 6 months
- Overall survival at 1 year in the subgroups (cutaneous, uveal, mucosal)
- To explore clinical efficacy of ipilimumab in relation to b-raf mutation status, brain metastases, LDH, HLA-A2 status

Exploratory objectives are

- To examine the value of peripheral blood absolute lymphocyte count (ALC) as a predictive biomarker in various patient cohorts with unresectable stage III-IV melanoma treated with ipilimumab monotherapy
- To evaluate possible surrogate markers in peripheral blood and tumour biopsy (translational research program)

Secondary Endpoint (s)

- All adverse events ≥ Grade 3 according to CTCAE, Version 4.0 criteria, that are definitely, probably, or possibly related to the administration of the investigational agents will be assessed.
- Overall response rate (PR+CR) according to immune-related response Criteria (ir-RC) at any time in the study.
- Disease control rate irDCR (rate of CR or PR or SD) according to immune-related response Criteria (ir-RC) at any time in the study.
- Overall response rate (PR+CR) according to RECIST criteria at any time in the study.
- Disease control rate (CR or PR or SD) according to RECIST criteria at any time in the study.
- Progression free survival rate at 6 months, PFS is defined as the time from the first study treatment date until the first documented tumor progression date (as defined by immune related response criteria) or date of death, whichever occurs first.
- Overall survival
- Clinical efficacy parameter (1-year OS, ORR, DCR, PFS at 6 months) according to b-raf mutational status of tumor (b-raf V600E yes versus no), brain metastases, LDH and HLA-A2

Exploratory Endpoints

- Pharmacodynamic effects of ipilimumab on Absolute Lymphocyte Count (ALC) and correlation of ALC to anti-tumor activity (ORR) of ipilimumab during induction phase (W12)
- Evaluation of possible surrogate markers in peripheral blood and tumour biopsy (translational research program)

Sample Size Justification

Assuming that the entire study cohort of an estimated 200 patients will behave as pretreated cutaneous melanoma patients treated with 3 mg/kg and re-induction for eligible patients as in MDX010-20 trial where the median survival rate of 10.1. months and a 1-year-survival rate of 45.6 % (0.5) was seen, the width of the 95% CI will maximally be 14%, corresponding to a lower confidence limit of 0.43 and an upper of 0.57.

The study includes patient subgroups (uveal, mucosal, with or without asymptomatic brain metastases, with or without elevated LDH levels) for which we currently have only limited efficacy data for ipilimumab on. Retrospective studies suggest the median survival for mucosal melanoma in Stage IV is around 6 months (corresponding assuming an exponential distribution) to about 13% survival rate at 1 year), a median survival for uveal melanoma is around 11 months (corresponding

to about 25% survival rate at 1 year) and a median survival for asymptomatic brain metastases is around 5 months (corresponding to about 13% survival rate at 1 year) (Penel et al., Invest New Drugs, 2008; Becker et al., Brit J Cancer, 2002; Schadendorf et al., Ann Oncol 2006).

Given the differing median survivals for uveal and mucosal melanoma prior to treatment with ipilimumab, the OS at 1 year will be presented separately for the cohorts, uveal, mucosal and cutaneous melanoma with or without asymptomatic brain metastases. Assuming 10% (N=20) subjects enrolled have uveal melanoma, and a further 10% have mucosal melanoma, whilst 80% (N=160) of those enrolled have cutaneous melanoma, the width of the 95%CI will, for any survival rate at 1 year, maximally span 41% for the uveal and mucosal cohorts, and 18% for the cutaneous cohort.

Statistics

The primary endpoint is the one year survival rate.
As a simple rate, it will be calculated as the number of patients being still alive 12 months after their first administration of the study treatment divided by the number of patients having received at least one administration of the study treatment. Only subjects known to be alive at 1 year will be included in the numerator. Exact (Clopper and Pearson) two-sided 95%CI will be given.
As a time to event variable, it will be derived from the Kaplan-Meier curve based on the survival data from all subjects receiving at least one administration of the study treatment. In this case missing data will be censored at the last contact. Two-sided 95%CI will be given.

Apart from the primary endpoint descriptive statistics will be used.

Results will be presented overall and per cohort (cutaneous versus uveal versus mucosal melanoma). Results may also be presented for the subgroups:

(1) – LDH < 2 ULN versus LDH ≥ 2 ULN
(2) - status brain mets versus no brain mets in medical history
(3) - b-raf mutation positive versus b-raf mutation negative
(4) - HLA-A2 positive versus HLA-A2 negative

Proportions (Survival rate at 1 year, irOR, irDCR, irPFS at 6 mths) will be presented with exact (Clopper and Pearson) two-sided 95% CI. irOR, irDCR and irPFS are defined according to the irRC. The date of origin for the survival rate at one year and irPFS at 6 months is the date of the first ipilimumab dose. Only subjects known to be progression free at 6 months / alive at 1 year will be included in the respective numerators. Any subject receiving at least one dose of ipilimumab will be included in the denominators.

Additional analyses treating survival and PFS as time to event endpoints will be performed i.e.: Kaplan-Meier plots will be produced. Survival will be measured, regardless of any re-induction, from the date of the first ipilimumab dose given on-study until date of death. Survival time for subjects whose date of death is unknown, will be censored at the last date of contact. PFS will be measured, regardless of any re-induction, from the date of the first dose of ipilimumab until the first on-study documented date of progression as per irRC, or date of death, whichever comes first. PFS time of subjects not known to progress or die, will be censored at the date of the last tumor assessment. Median survival, median PFS, survival rate at 1 year and PFS rate at 6 months, all as derived from the Kaplan-Meier curves, will also be presented, together with two-sided 95%CI.

Safety will be reported for 70 days post last treatment. This period will be considered the on-study safety period. The analysis of safety will be based on the frequency of AEs, relationship to ipilimumab and their severity for all treated subjects (i.e.: all subjects receiving at least one dose of ipilimumab). Worst toxicity grades per subject will be tabulated for all AEs. Additionally, drug-related grade 3 or more AEs, immune-related, and serious AEs will be tabulated separately, and listings by subject will be produced for all deaths, all SAEs and all AEs leading to discontinuation of study drug.

Time and Events Schedule for Protocol

Procedure	Screening / Pre-Treatment Visit	During Treatment Visits[a] [Induction and Re-Induction]: d 1 (W1), d 22 (W4), d 43 (W7), d 64 (W10) (+/- 7 days)	During Re-Staging-Visits[b] (Week 12/24/ 36/48)	End of Treatment Visit X (10 weeks after last administration)	FU-Phase[m] (every 3 months)	Protocol Section
**Eligibility Assessments**						
Tumour Biopsy for b-raf mutation status[j]	X					
Informed Consent	X					13.3
Inclusion/Exclusion Criteria	X					4.1 + 4.2
Medical History	X					6.1.1.1
**Safety Assessments**						
Physical Examination	X	X	X	X	X	6.1.1.1 - 6.1.1.4
Vital Signs[c]	X	X	X	X		6.1.1.1 - 6.1.1.4
ECOG Performance Status	X	X	X	X		6.1.1.1 - 6.1.1.4
ECG	X	(X)[l]	X			
Assessment of Signs and Symptoms	X	X	X	X		6.1.1.1; 6.1.1.4
Adverse Events Assessment		X	X			6.2+ 8.
Laboratory Tests[d, e]	X	X	X	X		6.1.1.1 - 6.1.1.4
Urinalysis (urine dipstick for protein)	X	X	X			6.1.1.2; 6.1.1.3
Urine β-HCG-Pregnancy Test[f]	X		X			6.1.1.1; 6.1.1.2

Time and Events Schedule for Protocol

**Procedure**	**Screening / Pre-Treatment Visit**	**During Treatment Visits[a] [Induction and Re-Induction]:** d 1 (W1), d 22 (W4), d 43 (W7), d 64 (W10) **(+/- 7 days)**	**During Re-Staging-Visits[b] (Week 12/24/ 36/48)**	**End of Treatment Visit X (10 weeks after last administration)**	**FU-Phase[m] (every 3 months)**	**Protocol Section**
**Efficacy Assessments (according to irRC and RECIST criteria, respectively)**						
MRT/CT-brain[g, h]	X		X			6.1.1.1; 6.1.1.3; 6.3.2
MRT/CT-thorax/abdomen/pelvis[g, h]	X		X			6.1.1.1; 6.1.1.3; 6.3.2
**Clinical Drug Supplies**						
Dispense Study Treatment		X				5.
Survival assessment		X	X	X	X	6.1.1.5
**Translational Research Programm (separate informed consent)**						
Whole venous blood 20ml	X		X[n]			6.4
EDTA “full blood” 2 ml	X[i]					6.4
Tissue biopsy (an additional fresh biopsy is optional)	X[j]					6.4

[a/b] Includes visits during induction and re-induction periods of the study.
[c] Blood pressure every 30 minutes during ipilimumab + 1 hour following completion of infusion (Sect. 6.2.1.2)
[d] All period laboratory samples must be collected within a window of up to 3 days before administration of ipilimumab
[e] TSH, free T4 at baseline, Visit 2, 3, 4, 5. **The assessment of TSH at baseline and before each treatment is mandatory. The TSH level must be available to the investigator prior to each administration.**
[f] A pregnancy test must be conducted and a negative result must be confirmed before the first administration of ipilimumab and after week 12
[g] within 6 weeks before start of therapy.
[h] The same method of assessment must be used throughout the trial to ensure comparability
[i] EDTA “full blood” for SNP, HLA-A2 typing once at the beginning of the study or any other time point of the study
[j] if not available until then, for b-raf status of tumor biopsy
[k] laboratory parameters: evaluation for LDH and S-100 should be performed at baseline, visit 2,3,5 (week 4, 7, 12)
[l] at baseline and week 12, if clinically indicated (evident of myocarditis e.g. dyspnoea, functional insufficiency) to be done before each treatment
[m] FU-phase is defined after initial progress and non-eligibility for re-induction. For each subject, survival status (alive/dead and date) will be collected every 3 mths until 1 year post the subjects’s first treatment dose.
[n] Serum and plasma only at screening and at week 12

1 INTRODUCTION

1.1 Clinical Experience and Research Hypothesis

Various phase II clinical studies using ipilimumab in stage IV have been conducted. Results of the first pivotal phase III study MDX010-20 have been presented at ASCO 2010 (O´Day et al., JCO; 2010) and are published in NEJM (Hodi et al., NEJM, 2010). In the second ongoing phase III trial CA184-024, a different dose and treatment scheme is currently tested. Results are expected at the end of 2010.

However, due to strictly defined inclusion and exclusion criteria in phase III trials, results of registrational trials might not answer all questions regarding safety and efficacy of ipilimumab in all advanced melanoma patient cohorts seen in daily routine. E.g. on one hand, in MDX010-20 trial, only patients positive for HLA-A2 could be enrolled (since patients were randomized in a double-blind fashion in a 1:3:1 ratio to receive HLA2-restricted melanoma gp 100 peptide vaccine monotherapy, MDX-010 in combination with melanoma gp100 peptide vaccine, or MDX-010 monotherapy.) In the second currently ongoing phase III study CA184-024 patients were not restricted to HLA-A2+ haplotype. However, another dosing and treatment scheme was tested (combination with DTIC; ipilimumab 10mg/kg and maintenance period following induction phase for patients who benefitted from ipilimumab) Moreover, only previously untreated patients could be enrolled. Patients with primary mucosal melanoma were excluded in CA184-024 study; patients with primary ocular melanoma were excluded in both MDX-010-20 as well as in CA184-024.

The currently described study concept with ipilimumab 3mg/kg would make a rational therapeutic strategy in a broad range of pretreated metastatic melanoma patients seen in daily clinical practice promising an efficacious and safe strategy in the therapy of the advanced stage malignant melanoma.

1.2 Product Development Rationale

A large clinical phase II is proposed to treat a broad range of metastatic melanoma patients who have failed or did not tolerate at least one prior systemic treatment in metastatic or non-resectable stage III disease in order to gain more information about and further characterize efficacy and safety of ipilimumab in a broad range of clinically relevant patient cohorts seen in daily routine, neither addressed in MDX-010-20 study nor in CA184-024. Enrollment will not be restricted to any specific HLA subtype and patients with uveal or ocular melanoma can be treated with ipilimumab as well. Furthermore patients with brain metastases and documented control for at least 3 months will be eligble. Potential predictive markers (ALC, b-raf mutation

status) will be prospectively evaluated. Wide access to ipilimumab in skin cancer-specialized clinical centers will be achieved in addition to a standardized evaluation of its clinical results and currently employed algorithms so cope with occurring adverse events. Patients will be treated according the MDX-010-20 protocol with ipilimumab monotherapy 3mg/kg four times during the induction phase only. Patients who benefit from therapy (stable disease of ≥ 3 months beginning at Week 12 or patients with initial partial or complete response at Week 12 assessment) and did not show any immune-related adverse event (irAE) ≥ grade 3 will be eligible for treatment re-induction in the case of melanoma relapse. RE-induction will be part of the study if relapse occurs during 12 months after first receipt of study medication. Re-induction will not be part of the protocol if relapse is experienced after individual end of trial (12 months post first receipt of study medication)

1.2.1 CTLA-4 and T Cell Activation

Figure 1 Mechanism of Action

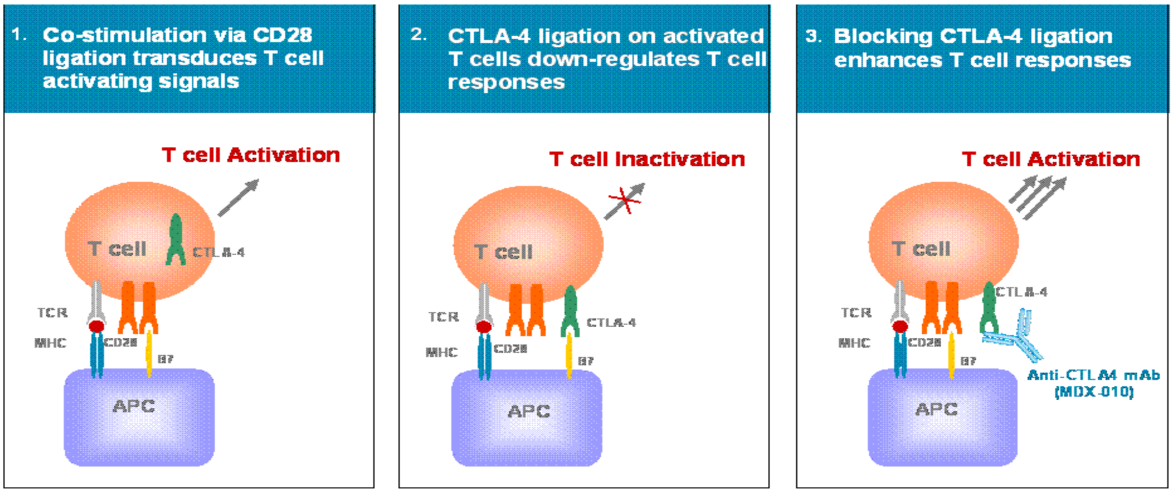

Advances in the understanding of the mechanisms that regulate T cell activation have allowed the rational design of new strategies for immunotherapy of tumors, including melanoma. It has been known for some time that engagement of the T cell antigen receptor by itself is not sufficient for full T cell activation; a second co-stimulatory signal is required for induction of IL-2 production, proliferation and differentiation to effector function of naive T cells. Abundant data now indicate that the primary source of this costimulation is mediated by engagement of CD28 on the T cell surface by members of the B7 family on the antigen-presenting cell (APC) (Lenschow et al., 1996). (See Figure 1)

Expression of B7 has been shown to be limited to “professional” antigen presenting cells; that is, specialized cells of the hematopoietic lineage, including dendritic cells, activated macrophages, and activated B cells. It has been suggested that this sharply-defined restriction

of B7 expression is a fail-safe mechanism for maintenance of peripheral T cell tolerance, insuring that T cell activation can only be stimulated by appropriate APCs (Schwartz et al., 1992). The fact that tumor cells do not express B7 contributes to their poor capacity to elicit immune responses (Chen et al., 1992; Townsend et al., 1993).

The demonstration that induction of expression of B7 on many tumor cells by transfection, transduction, or other mechanisms can heighten tumor immunogenicity led to great interest in pursuing this as an approach to tumor immunotherapy. As demonstrated in vivo in murine tumor models, the utility of B7 expression as a vaccination approach is limited by the following factors: (1) B7-expressing tumor cell vaccines are only effective when the tumor cells have a high degree of inherent immunogenicity; (2) while B7-expressing vaccines have been shown in many cases to be effective in inducing protective immune responses, they have demonstrated only limited utility in inducing responses to established tumors; and (3) inactivation of tumor cells by radiation has been shown to destroy the immuno-enhancing activity of the B7 gene product (Townsend et al., 1994; Allison et al., 1995).

In the past few years it has become apparent that co-stimulation is even more complex than originally thought. After activation, T cells express CTLA-4, a close homologue to CD28. CTLA-4 binds members of the B7 family with a much higher affinity than CD28 (Linsley et al., 1991). Although there was initially some controversy as to the role of CTLA-4 in regulating T cell activation, it has become clear that CTLA-4 down-regulates T cell responses (Thompson & Allison, 1997). This was initially suggested by the following in vitro observations: (1) blockade of CTLA-4/B7 interactions with antibody enhanced T cell responses; (2) cross-linking of CTLA-4 with CD3 and CD28 inhibited T cell responses; and (3) administration of antibodies to CTLA-4 in vivo enhanced the immune response to peptide antigens or superantigens in mice (Walunas et al., 1994; Kearney et al., 1995; Krummel et al., 1995; 1996). Blocking CTLA-4-B7 interaction while preserving signaling via CD28 resulted in enhanced T cell responses in vitro (Kearney et al., 1995).

Perhaps the most convincing demonstration of the down-regulatory role of CTLA-4 came from examination of mice with a null mutation (Tivol et al., 1995; Waterhouse et al., 1995; Chambers et al., 1997). CTLA-4 knockout mice appear to have spontaneously activated T cells evident at approximately 1 week after birth, followed by rampant lymphoproliferation and lymphadenopathy. These mice die at approximately 3 weeks of age, either as a result of polyclonal T cell expansion and tissue destruction or as a result of toxic shock resulting from lymphokine production by the T cells. Since thymocyte differentiation and selection proceed normally in CTLA-4-deficient mice, the rampant T cell expansion that occurs in the mice indicates that CTLA-4 plays a critical role in down-regulating T cell responses in the periphery (Krummel et al., 1996)

1.3 Summary of Results of Investigational Program

1.3.1 Pharmacology of Ipilimumab

Ipilimumab is a human immunoglobulin G (IgG1)κ anti-CTLA-4 monoclonal antibody (mAb). In vitro studies were performed with ipilimumab to demonstrate that it is specific for CTLA-4, actively inhibits CTLA-4 interactions with B7.1 and B7.2, does not show any cross-reactivity with human B7.1, B7.2 negative cell lines, and stains the appropriate cells without non-specific cross-reactivity in normal human tissues, as demonstrated by immunohistochemistry. Ipilimumab does cross-react with CTLA-4 in non-human primates including cynomolgus monkeys.

Ipilimumab was originally produced and purified from a hybridoma clone. Subsequently, a transfectoma (CHO cell) has been generated that is capable of producing more ipilimumab on a per cell basis than the hybridoma. Material from the transfectoma will be utilized in this and future ipilimumab clinical studies. Biochemical, immunologic and in vivo preclinical primate assessments demonstrated similarity between hybridoma and transfectoma-derived ipilimumab.

1.3.2 Pre-Clinical Toxicology of Ipilimumab

Complete information on the pre-clinical toxicology studies can be found in the Ipilimumab Investigator Brochure (IB). Non-clinical toxicity assessments included in vitro evaluation for the potential of ipilimumab to mediate complement-dependent cellular cytotoxicity (CDCC) or antibody-dependent cellular cytotoxicity (ADCC), and toxicology assessments in cynomolgus monkeys alone and in the presence of vaccines.

The in vitro studies demonstrated that ipilimumab did not mediate CDCC of PHA- or (CD)3-activated human T cells. However, low to moderate ADCC activity was noted at concentrations up to 50 ug/mL. These data are consistent with the requirement of high levels of antigen expression on the surface of target cells for efficient ADCC or CDCC. Since ipilimumab is a human IgG1, an isotype generally capable of mediating CDCC and ADCC, the lack of these activities is likely due to a very low expression of CTLA-4 on activated T cells. Therefore, these data suggest that ipilimumab treatment would not result in depletion of activated T cells in-vivo. Indeed, no depletion of T cells or T cell subsets were noted in toxicology studies in cynomolgus monkeys.

No mortality or signs of toxicity were observed in three independent 14-day intravenous toxicology studies in cynomolgus monkeys at multiple doses up to 30 mg/kg/dose. Furthermore, ipilimumab was evaluated in sub chronic and chronic toxicology studies in cynomolgus monkeys with and without Hepatitis B (HepB) Vaccine and Melanoma Vaccine.

Ipilimumab was well tolerated alone or in combination in all studies. There were no significant changes in clinical signs, body weight values, clinical pathology values or T cell activation markers. In addition, there were no significant histopathology changes in the stomach or colon.

1.3.3 Human Pharmacokinetics of Ipilimumab

Pharmacokinetic (PK) profiles for ipilimumab have been analyzed. The primary objective of Protocol MDX010-015 was to determine the safety and PK profile of single and multiple doses of ipilimumab derived from a transfectoma or hybridoma cell line. Mean plasma concentrations of ipilimumab administered at doses of 3 mg/kg (hybridoma-derived drug product); 2.8 mg/kg, 5 mg/kg, 7.5 mg/kg, 10 mg/kg, 15 mg/kg, and 20 mg/kg (transfectoma-derived drug product) demonstrated approximate dose proportionality. Equimolar doses of hybridoma-derived and transfectoma-derived drug product had comparable PK profiles. The range of mean volume of distribution at steady state (Vss) across cohorts 2.8, 3, 5, 7.5, 10, 15, and 20 mg/kg, was 57.3 to 82.6 mL/kg, indicating drug distribution was mostly limited to the intravascular space. The clearance was low (range 0.11 to 0.29 mL/h/kg) and reflective of the half-life (range 297 to 414 h), which is consistent with the long terminal disposition phase of ipilimumab. There was moderate variability in the PK parameters among subjects, with CV of 11% to 48% in AUC(0-21d), 20% to 59% in CL, and 17% to 46% in Vss.

1.3.4 Clinical Safety with Ipilimumab

Ipilimumab immunotherapy is currently under investigation in patients with unresectable advanced melanoma (unresectable Stage III or Stage IV) to potentially demonstrate an improvement on a large unmet medical need in this population.

Ipilimumab has been administered to approximately 2901 patients with different cancers in 25 completed or ongoing clinical trials as of 31-Mar-2009 with a dose range between 0.3 mg/kg and 20 mg/kg and in various combinations.

In general, the safety profile of ipilimumab administered as single doses of up to 20-mg/kg and multiple doses of up to 10 mg/kg every 3 weeks was characterized by adverse reactions that were mostly immune in nature. Drug-related SAEs were reported in studies of ipilimumab administered as monotherapy, as well as in combination with vaccines, cytokines, chemotherapy, or radiation therapy.

The overall summary of safety for the 2901 patients treated with ipilimumab in the completed or ongoing clinical trials and the subset of 658 patients treated at the 10 mg/kg dose level is presented in Table 1.

Table 1: Ipilimumab - Overall Summary of Safety

	Number of Subjects (%)	
	**Ipilimumab 0.3 - 20 mg/kg N = 2901**	**Ipilimumab 10 mg/kg N = 658**
Any Drug-related AE	2357 (81.2)	561 (85.3)
Grade 1	699 (24.1)	158 (24.0)
Grade 2	889 (30.6)	198 (30.1)
Grade 3	617 (21.3)	163 (24.8)
Grade 4	127 (4.4)	38 (5.8)
Grade 5	20 (0.7)	4 (0.6)
Any Serious Adverse Events	1258 (43.4)	310 (47.1)
Grade 3 – 4	806 (27.8)	179 (27.2)
Any Drug-related Serious Adverse Events	595 (20.5)	179 (27.2)
Grade 3 – 4	469 (16.2)	140 (21.3)

1.3.4.a Details of Drug-Related Adverse Events

Treatment-emergent adverse events (AEs) considered by the investigator to be related to study drug were reported for 81.2% of all treated subjects and 85.3% of subjects treated with ipilimumab at 10 mg/kg.

Among all treated subjects, the most frequently reported treatment-related AEs of any grade included fatigue (27.8%), diarrhea (27.5%), nausea (23.4%), rash (21.8%), pruritus (19.9%), pyrexia (11.9%), and vomiting (11.7%).

Similarly, among subjects treated with ipilimumab at 10 mg/kg, the most frequently reported treatment-related AEs of any grade included diarrhea (38.1%), fatigue (30.5%), rash (34.5%), pruritus (29.8%), nausea (17.6%), pyrexia (12.3%), vomiting (10.9%). and colitis (10.2%).

1.3.4.b Details of Drug-Related Serious Adverse Events

Among all 2901 treated subjects, SAEs (serious adverse events) considered possibly, probably, or definitely related to study drug were reported for 20.5% of subjects. Drug-related SAEs reported in at least 1% of the 2901 subjects included diarrhea (5.8%), colitis (4.7%), ALT increased (2.3%), AST increased (2.2%), pyrexia (1.6%), and vomiting (1.3%). Among the 658 subjects who received ipilimumab at 10 mg/kg, SAEs considered possibly, probably, or definitely related to study drug were reported for 27.2% of subjects. Drug-related SAEs reported in at least 1% of the 658 subjects treated at 10 mg/kg included diarrhea (8.5%), colitis

(7.0%),vomiting (2.1%), AST increased (2.1%), ALT increased (2.0%), autoimmune hepatitis (2.0%), pyrexia (1.8%), hypopituitarism (1.7%), dehydration (1.7%), nausea (1.2%), and abdominal pain (1.1%).

1.3.4c Safety of Ipilimumab Every 3 Weeks for 4 Doses

As of 31 March 2009, 658 subjects with metastatic refractory melanoma have been enrolled to be treated with 10 mg/kg of ipilimumab every 3 weeks for four doses and followed for at least 12 weeks. The overall rate of irAEs (immune-related adverse event (irAE)), regardless of grade for subjects receiving 10 mg/kg, was 21.9% including 12.9%% with serious gastrointestinal irAEs, 5.1% with hepatic dysfunction and 3.2% with serious endocrinopathy. The most recent Ipilimumab Investigator Brochure (and addenda) should be consulted for the most current details of safety. More than 200 subjects with metastatic refractory melanoma from studies MDX 020 or CA184-022 were treated with monotherapy 3 mg/kg of ipilimumab every 3 weeks for four doses and followed for at least 12 weeks. The overall rate of irAEs from CA184-022 (n=71), regardless of grade, was 65% (58% Grade 1 or 2 and 7% Grade 3 or 4). The most common events were skin-related (44% Grade 1 or 2 and 1% Grade 3 or 4), gastrointestinal, including diarrhea and intestinitis (30% Grade 1 or 2 and 3% Grade 3 or 4). The formal safety analysis from the MDX-020 study (n=137) is published in Hodi et al., NEJM, 2010, however, no new safety signals were identified. The most recent ipilimumab Investigator Brochure (and addenda) should be consulted for the current details of safety.

1.3.5 Immune-Related Adverse Events (irAEs) with Ipilimumab

Many of the adverse events considered related to ipilimumab may be immune in nature and presumably a consequence of the intrinsic biological activity of ipilimumab. An irAE is defined as any adverse event associated with drug exposure and consistent with an immune-mediated event. Disease progression, infections and other etiologic causes are ruled out or deemed unlikely as contributing to the event. Supportive data, such as autoimmune serology tests or biopsies, are helpful but not necessary to deem an event an irAE. Events of unclear etiology which were plausibly “immune-mediated” have been conservatively categorized as irAEs even if serologic or histopathology data are absent. These irAEs likely reflect a loss of tolerance to some self antigens or an unchecked immune response to gut or skin flora. Some breakthrough of immunity may be inseparably linked to the clinical antitumor activity of ipilimumab.

Immune-related AEs predominately involve the GI tract, endocrine glands, liver or skin.

Among all 2901 treated subjects, 59.6% (1729/2901) of subjects reported any irAE and 15.2% (441/2901) of subjects reported serious irAEs. Among subjects who received ipilimumab at 10 mg/kg, 21.9% (144/658) of subjects reported serious irAEs.

Table 2 summarizes the incidence of serious irAEs among all treated subjects and subjects who received ipilimumab 10 mg/kg.

Table 2: Serious Immune-related Adverse Events Reported for at Least 2% of Subjects in any Event Category

	Number of Subjects (%)	
	**Ipilimumab 0.3 - 20 mg/kg**	**Ipilimumab 10 mg/kg**
	**N = 2901**	**N = 658**
irAEs[a]		
Any	441 (15.2)	144 (21.9)
Grade 3	298 (10.3)	87 (13.2)
Grade 4	59 (2.0)	25 (3.8)
GI irAE[a]		
Any	236 (8.1)	85 (12.9)
Grade 3	166 (5.7)	58 (8.8)
Grade 4	17 (0.6)	10 (1.5)
Liver irAE[a]		
Any	109 (3.8)	33 (5.0)
Grade 3	72 (2.5)	18 (2.7)
Grade 4	32 (1.1)	13 (2.0)
Endocrine irAE[a]		
Any	61 (2.1)	21 (3.2)
Grade 3	44 (1.5)	12 (1.8)
Grade 4	3 (0.1)	1 (0.2)

[a]Based on treatment-related adverse events retrieved from the clinical database using pre-defined MedDRA terms that were considered potential irAEs.

With few exceptions, irAEs were clinically manageable and reversible with supportive care or corticosteroids. Management algorithms are included in the investigator broschure (IB).

Corticosteroid treatment did not adversely affect antitumor responses in those subjects who had both an irAE requiring steroid therapy and an objective tumor response. Systemic corticosteroids do not appear adversely associated with ipilimumab-induced clinical response when used to manage irAEs in patients with advanced melanoma. Similar results were observed regardless of whether mWHO or the novel irRC criteria were used. Steroids can be used promptly to manage severe irAEs and minimize the risk for serious complications (Asim Amin et al., 2009).

In the setting where subjects were enrolled to receive ipilimumab every 3 weeks dosing until progression, irAEs could be reported at any time, with colitis and rash reported most often during the early doses and hypophysitis reported with later doses.

Gastrointestinal irAEs

The most common Grade 3 or greater irAE involved the lower GI tract and clinically manifested as diarrhea or hematochezia. Diarrhea resulting from treatment with ipilimumab ranged from mild to severe and was life-threatening in some cases. Some cases of diarrhea began as mild and became very severe. Among subjects who received ipilimumab at 10 mg/kg, GI irAEs of any grade were reported for 40.0% (263/658) of subjects, and Grade 3 - 4 GI irAEs were reported for 12.6% (83/658) of subjects. Serious GI irAEs, mostly involving diarrhea or colitis, were reported in 12.9% (85/658) of subjects treated with ipilimumab at 10 mg/kg.

Inflammatory Hepatotoxicity

Immune-related hepatic dysfunction, including hepatitis or abnormal liver function tests (LFT) attributed to ipilimumab therapy, has been reported. Subjects may develop elevations in LFTs in the absence of clinical symptoms. Inflammatory hepatotoxicity includes non-infectious hepatitis (eg, autoimmune hepatitis). Among subjects who received ipilimumab at 10 mg/kg, inflammatory hepatotoxicity of any grade was reported for 9.0% (59/658) of subjects, and Grade 3 - 4 inflammatory hepatotoxicity was reported for 6.4% (42/658). Serious inflammatory hepatotoxicity has been reported in 5.0% (33/658) of subjects who received ipilimumab at 10 mg/kg. Inflammatory hepatotoxicity is usually reversible when immediately treated with high-dose steroids, if applicable, with or without additional immunosuppressants as recommended in the hepatotoxicity management algorithm presented as an appendix in the IB.

Hypophysitis/Hypopituitarism and Other Endocrine Conditions

Hypophysitis/hypopituitarism, clinically manifested by fatigue, has been reported. Most subjects with hypopituitarism presented with nonspecific complaints such as fatigue, confusion, visual disturbance, or impotence. Some had headache as the predominant presentation. The majority of subjects with hypopituitarism demonstrated enlarged pituitary glands based on brain magnetic resonance imaging (MRI). Low adrenocorticotropic hormone (ACTH) and cortisol were the most common biochemical abnormality reported; low thyroid stimulating hormone (TSH), testosterone, or prolactin was also reported in some subjects (Blansfield et al., 1995).

Hypophysitis/hypopituitarism was controlled with appropriate hormone-replacement therapy and may be dose related. Among subjects who received ipilimumab at 10 mg/kg,

endocrinopathy of any grade was reported for 7.6% (50/658) of subjects, and Grade 3-4 endocrinopathy was reported for 2.4% (16/658) of subjects. Serious drug-related endocrinopathy, such as hypophysitis/hypopituitarism, was reported in 3.2% (21/658) of subjects who received ipilimumab at 10 mg/kg.

The first onset of endocrine irAEs typically occurred between weeks 6 and 12 of treatment. Endocrine events were generally manageable with hormone-replacement therapy, and the majority of subjects were not weaned from steroids.

Rash and Other Skin Conditions

Rash was one of the most common irAEs, and most cases were Grade 1 or 2 in intensity; pruritus has also been reported (Jaber et al., 2006). When biopsied, pleomorphic infiltrates were noted in the skin. Among subject who received ipilimumab at 10 mg/kg, skin irAEs of any grade were reported for 52.9% (348/658) of subjects, and Grade 3 - 4 skin irAEs were reported for 2.9% (19/658) of subjects. Serious skin irAEs were reported in < 1% (4/658) of subjects who received ipilimumab at 10 mg/kg. Skin irAEs were generally reversible.

Other presumed irAEs reported include, but were not limited to, arthritis/arthralgias, pneumonitis, pancreatitis, autoimmune (aseptic) meningitis, autoimmune nephritis, pure red cell aplasia, noninfective myocarditis, occular inflammation, Guillain-Barre syndrome (GBS), myasthenia gravis, and neuropathy (eg, motor neuropathy, neuritis), of which were individually reported for < 1% of subjects.

Other reported irAEs

Ocular inflammation, manifested as Grade 2 or Grade 3 episcleritis or uveitis, was associated with concomitant diarrhea in a few subjects and occasionally occurred in the absence of clinically apparent GI symptoms (Robinson et al., 2004). Serious ocular inflammation was reported in 1 of 658 (0.2%) subjects who received ipilimumab at 10 mg/kg (8 [0.3%] of 2901 subjects program-wide reported serious occular inflammation). Preliminary analysis (based on the manual extraction of the SAE data from the internal safety database) indicated that the median time to event onset was approximately 61 days (range: 14 - 114 days). Based on the available data with known outcome, most of the subjects recovered or improved with or without corticosteroid therapy with a median duration of around 6 days (range: 5 - 23 days).

Management algorithms for general irAEs, and ipilimumab-related diarrhea, hepatotoxicity, endocrinopathy, and neuropathy are pesented as appendices in the current IB.

Additionally, as of February 2006, there has been observation from a National Cancer Institute (NCI) study of bowel wall perforation in some patients who were administered a high-dose IL-2 following treatment with ipilimumab. Of the 22 patients administered high-dose IL-2, three patients experienced bowel wall perforations. This is a higher rate than would be expected with high-dose IL-2 treatment alone. All three patients had metastatic melanoma and had previously received their last dose of ipilimumab > 77 days before the first dose of IL-2. Two of the patients had clinically significant ipilimumab-related diarrhea or colitis and the symptoms had completely resolved prior to IL-2 administration. One patient did not experience ipilimumab-related diarrhea. It is unknown whether this observation represents a true association or is mechanistically unrelated to prior ipilimumab exposure.

1.3.5.a Drug-Related Deaths

Based on reports from the safety data base as of June 30, 2009, there have been reports of death (approximately 1% [35/3800]), deemed by the investigator as possibly related to the administration of study drug. The most common cause of drug related deaths was GI perforation. Other causes included multiorgan failure, sepsis, hypotension, acidosis, Guillain-Barré-Syndrome and adult respiratory distress syndrome. The most recent Ipilimumab Investigator Brochure (and its addenda) should be consulted for the most current details of safety, including mortality data.

1.3.5.b Dose-Dependent Incidences of Immune-Related Adverse Events

Based on a review of the program-wide SAE data as previously reported, evidence had suggested that ipilimumab-associated irAEs were dose dependent in frequency, and higher irAE rates had been observed at 10 mg/kg than at lower doses of ipilimumab. Subsequently, this dose-dependent effect was further demonstrated in CA184-022 in which three dose levels of ipilimumab were studied, including 0.3 vs 3 vs 10 mg/kg (Wolchok et al., 2010).. Table 3 summarizes the overall irAE frequencies by dose from CA184-022 based on safety data from the locked clinical database.

Qualitatively, the safety profile of ipilimumab at 10 mg/kg remains consistent with the low-dose safety profile in that most of the drug-related SAEs are characteristic of immune-related toxicity, and most of the irAEs are reported in the GI, hepatic, and endocrine systems. However, the data presented in Table 3 suggest that the frequency of irAEs in association with 10 mg/kg of ipilimumab at multiple doses is higher compared with the irAE frequency reported for lower doses.

Table 3. Summary of Immune-Related Adverse Events (irAEs) by Treatment Groups - Treated Subjects (CA184-022)

	Number of Subjects (%)		
	Ipilimumab		
	**0.3 mg/kg (N=72)**	**3 mg/kg (N=71)**	**10 mg/kg (N=71)**
Overall irAEs	26.4	64.8	70.4
Grade 3-4	0	7.0	25.4
GI irAEs	16.7	32.4	39.4
Grade 3-4	0	2.8	15.5
Hepatic irAEs	0	0	2.8
Grade 3-4	0	0	2.8
Endocrine irAEs	0	5.6	4.2
Grade 3-4	0	2.8	1.4
Skin irAEs	12.5	45.1	46.5
Grade 3-4	0	1.4	4.2

1.3.5.c Neuropathies

Isolated cases of motor neuropathy of an autoimmune origin have been reported among patients treated with ipilimumab. Four cases have been diagnosed as Guillain-Barre syndrome (GBS) including one in the MDX010-020 trial, two of which were considered study related. In both cases, the GBS was atypical in nature and more clinically resembled polyneuritis. As of 30 June 2009, 27 cases of neuropathy SAEs have been reported. Of these, 22 were assessed as unrelated to study therapy because alternative etiologies, including brain metastases, spinal cord compression, aterial thrombosis, or platinum-base chemotherapy were identified in almost every case.

1.3.6 Clinical Efficacy of Ipilimumab

Treatment with ipilimumab has demonstrated clinically important and durable tumor responses in several malignancies including melanoma, prostate cancer, and renal cell carcinoma. The most extensively studied tumor type has been malignant melanoma and the principal demonstration of the efficacy of ipilimumab at the 10 mg/kg dose comes from 3 Phase 2 multicenter trials in 487 subjects with advanced melanoma: CA184022 (Wolchok et al., 2010; CA184022 Clinical Study Report 2008), CA184008 (O´Day et al., Ann Oncol 2010; CA184008 Clinical Study Report, 2008) and CA184007 (CA184007 Clinical Study Report).

CA184022 was a dose-ranging trial in which subjects were randomized (1:1:1) to a high (10 mg/kg) intermediate (3.0 mg/kg), and low (0.3 mg/kg) ipilimumab dose (Wolchok et al., 2010).

CA184022 and CA184008 enrolled pretreated subjects and efficacy data at 10 mg/kg are pooled for these studies; CA184007 enrolled a mixed population of pretreated and previously untreated subjects. In all 3 studies, ipilimumab as monotherapy was administered by intravenous (IV) infusion every 3 weeks for 4 doses (induction), followed by a dose every 12 weeks (maintenance).

The overall survival (OS) results from these 3 completed primary Phase 2 studies are presented in Table 4

Table 4 Updated Overall Survival Results for 3 Primary Studies in Advanced Melanoma (as of 09-Mar-2009)

	CA184022	CA184008	CA184007	
	All Randomized	All Treated	All Randomized[a]	
**Parameter**	**Ipilimumab 10 mg/kg** N = 72	**Ipilimumab 10 mg/kg** N = 155	**Ipilimumab 10 mg/kg + Placebo** N = 57	**Ipilimumab 10 mg/kg + Budesonide** N = 58
OS, Median (Months)<br>95% CI (Months)[b]	11.43<br>(6.90, 16.10)	10.22<br>(7.59, 16.30)	19.29<br>(11.99, --)	17.68<br>(6.80, --)
Survival Rate at 1 Year (%)<br>95% CI (%)[c]	48.64<br>(36.84, 60.36)	47.22<br>(39.52, 55.11)	62.41<br>(49.37, 75.07)	55.87<br>(42.71, 68.79)
Survival Rate at 2 Years (%)<br>95% CI (%)[c]	29.81<br>(19.13. 41.14)	32.83<br>(25.37, 40.49)	41.78<br>(28.30, 55.46)	40.57<br>(27.12, 54.37)

[a] Data are presented for the per-protocol mixed population of pretreated and previously untreated subjects.

[b] Based on Kaplan-Meier estimation and CI computed using the bootstrap method.

[c] Median and associated 2-sided 95% CIs calculated using the method of Brookmeyer and Crowley.

Consistent with the known mechanism of action of ipilimumab, the reduction in tumor burden in the 3 primary melanoma studies was characterized by novel patterns of measurable clinical effect. In addition to objective response (complete response [CR] and partial response [PR]), and SD as measured by mWHO, novel patterns of clinical activity, which are related to the immunological mechanism of action of ipilimumab, were reported in all 3 studies. These patterns of activity were characterized by overall reductions from baseline in total tumor burden (index plus measurable new lesions, when present) after the appearance of new lesions and/or after an initial tumor burden increase. Of 227 treated subjects who received 10 mg/kg ipilimumab in studies CA184022 and CA184008, 9.7% of subjects demonstrated disease control after an initial increase in tumor burden and/or appearance of new lesions

(Wolchok et al., 2010; O´Day et al., Ann Oncol 2010; CA184022 Clinical Study Report 2008; CA184008 Clinical Study Report, 2008; CA184007 Clinical Study Report 2008).

Survival data from these 3 primary studies (CA184022, CA184008, and CA184007) are supported by OS data from CA184004 and MDX-010-28. Both studies enrolled previously untreated and pretreated subjects with advanced melanoma. In CA184004, 42 subjects were randomized to 10 mg/kg ipilimumab. The median follow-up for survival in this study was 8.6 months. The median OS was 11.8 months (95% CI: 6.1, --); the 1-year survival rate was 44.2% (95% CI: 24.1%, 64.1%) (CA184004 Clinical Study Report, 2008). Data are available for 23 response-evaluable subjects treated at 10 mg/kg ipilimumab in MDX010-15 who enrolled in the follow-up study MDX010-28. The median follow-up for survival in this study was 13.4 months. The median OS was 13.2 months (95% CI: 9.4, 19.4). The overall survival for 2 subjects with ongoing responses was 26.0+ months and 25.0+ months (MDX010-19 Clinical Study Report, 2007).

In this protocol the proposed induction dosing of 3 mg/kg every 3 weeks for 4 doses followed by reinduction for eligible patients is based on the Phase 3 study MDX010-20 published in NEJM 2010 (Hodi et al.) and supported by the safety and activity of 3 mg/kg ipilimumab from two Phase 2 studies (CA184022 and CA184004). MDX010-20 demonstrated a statistically significant and clinically meaningful survival benefit with a hazard reduction for death of 34% (hazard ratio of 0.66) in subjects with pre-treated advanced melanoma who received ipilimumab monotherapy with a 1-year survival rate of 45% and a 2-year survival rate of 23% which was statistically highly significant compared tot he control arm (Hodi et al., 2010).

Further, clinical activity was also observed upon re-induction of ipilimumab therapy in MDX010-20 (Hodi et al., 2010). In parallel with MDX010-20, the randomized, Phase 2, dose-ranging study CA184022 investigated doses of 0.3, 3, and 10 mg/kg doses administered every 3 weeks for 4 doses (induction) followed by one dose every 12 weeks (maintenance). There was increasing clinical activity with increasing doses of ipilimumab based on the PK, PD,19 and BORR (Wolchok et a., Lancet Oncology 2010). The results were consistent with those from MDX010-20 and across the Phase 2 program.

The regimen of 3 mg/kg ipilimumab every 3 weeks for 4 doses used in the Phase 3 study is the best characterized to date, with ipilimumab demonstrating an OS benefit. Therefore, the recommended dose and schedule of ipilimumab for the treatment of patients with advanced melanoma who have received prior therapy reflects that used in the pivotal Phase 3 study: 3 mg/kg ipilimumab administered IV over a 90-minute period every 3 weeks for a total of four doses as tolerated (induction), with additional treatment (re-induction of the same dose and schedule as tolerated) considered for patients who develop progressive disease after prior complete response, partial response, or stable disease lasting longer than 3 months from the first tumor assessment.

Further details on clinical results can be found in the current version of the Ipilimumab Investigator Brochure.

1.3.6.a Relationship Between Response and Immune Related Events in Patients with Metastatic Melanoma

Drug-related AEs of any grade considered to be immune-mediated in nature (irAEs) were reported for 59.6% (1729/2901) of subjects in clinical studies of ipilimumab. These irAEs are a consequence of inhibiting CTLA-4 function and most were reported as Grade 1 or 2. An association between best overall response (BORR) and higher grade (Grade 3-4) irAEs was suggested in early studies of ipilimumab 3 mg/kg but this association was not observed in 4 Phase 2 studies of ipilimumab administered at 10 mg/kg (CA184022, CA184008, CA184007 and CA184004). There were proportionally more subjects with irAEs of any grade who experienced response or stable disease than subjects without irAEs who experienced response or stable disease, but due to the small sample sizes, these observations were statistically inconclusive (Wolchok et al., 2010; O´Day et al., Ann Oncol 2010; CA184022 Clinical Study Report 2008; CA184008 Clinical Study Report, 2008; CA184007 Clinical Study Report 2008). Generally, disease control and long term survival are observed among patients regardless of whether they do or do not develop Grade ≥ 2 irAEs.

1.3.7 Rationale for using immune-related Tumor Assessment Criteria (irRC)

Ipilimumab is an immuno-stimulating antibody with evidence of anti-cancer activity (durable objective response and stable disease) in subjects with advanced melanoma, including subjects with established brain disease. Observations of clinical activity defined by tumor response endpoints defined by conventional criteria (eg, mWHO) may be inadequate to realize the kinetics and magnitude of clinical benefit achieved with ipilimumab.

Histopathological evidence has demonstrated ipilimumab can produce an influx or expansion of tumor infiltrating lymphocytes (Townsend et al., 1993). Therefore, early increases in lesion size detected radiologically or upon gross examination could be misinterpreted as progressive tumor growth and precede objective tumor shrinkage. In addition the appearance of new lesions may have categorized a subject to have progressive disease using conventional tumor assessment criteria despite the concurrent observation of objective tumor responses in preexisting lesions and a net reduction in global tumor burden that includes the new lesions.

Hence the appearance of new lesions in and of themselves may not necessarily constitute progressive disease. The immune-related response criteria (irRC) were developed as a tool to gauge tumor response using the changes in global tumor burden (Wolchok et al., 2009). In addition, the irRC may be useful to inform a physician’s decision to continue dosing in subjects who may receive benefit from additional ipilimumab therapy. The ir-response assessment is

based solely on objective measurements (SPD) of index and new lesions. Non-index lesions are not considered. The irRC identified 9.7% of subjects (22/227 treated subjects) from CA184022 and CA184008 at 10 mg/kg who demonstrated disease control in the form of stable or reduced measurable tumor burden, including new lesions, at or after disease progression by mWHO.

1.4 Overall Risk/Benefit Assessment

As indicated above, ipilimumab has increasing clinical activity with increasing doses, however, there is also an increasing incidence of serious adverse events by increasing dosage.

The regimen of 3 mg/kg ipilimumab every 3 weeks for 4 doses used in the Phase 3 study (MDX010-020) is the best characterized to date, with ipilimumab demonstrating an OS benefit (Hodi et al., NEJM, 2010). Therefore, the recommended dose and schedule of ipilimumab for the treatment of patients with advanced melanoma who have received prior therapy reflects the regimen used in the pivotal Phase 3 study: 3 mg/kg ipilimumab administered IV over a 90-minute period every 3 weeks for a total of four doses as tolerated (induction), with additional treatment (re-induction of the same dose and schedule as tolerated) considered for patients who develop progressive disease after prior complete response, partial response, or stable disease lasting longer than 3 months from the first tumor assessment and no severe toxicity experienced.

The proposed induction dosing in this study of 3 mg/kg every 3 weeks for 4 doses followed by re-induction for eligible patients is based on the Phase 3 study MDX010-20 and supported by the safety and activity of 3 mg/kg ipilimumab from two Phase 2 studies (CA184022 and CA184004). MDX010-20 demonstrated a statistically significant and clinically meaningful survival benefit with a hazard reduction for death of 34% (hazard ratio of 0.66) in subjects with pre-treated advanced melanoma who received ipilimumab monotherapy (Hodi et al., NEJM, 2010) in comparison to gp100 monotherapy arm. Further, clinical activity was also observed upon re-induction of ipilimumab therapy in MDX010-20 (Hodi et al. JCO 2010) In parallel with MDX010-20, the randomized, Phase 2, dose-ranging study CA184022 investigated doses of 0.3, 3, and 10 mg/kg doses administered every 3 weeks for 4 doses (induction) followed by one dose every 12 weeks (maintenance) (Wolchok et al., 2010). There was increasing clinical activity with increasing doses of ipilimumab based on the PK, PD, and BORR (Wolchok et al., 2010). The results were consistent with those from MDX010-20 and across the BMS Phase 2 program.

Characteristic organ-specific inflammatory irAEs were reported with ipilimumab therapy, typically during induction therapy. IrAEs were mostly reversible within days to weeks following cessation of therapy or treatment with symptomatic therapy, corticosteroids or other anti-inflammatory agents, depending upon severity. Accumulated clinical experience resulted in

detailed toxicity management guidelines (also termed algorithms), by use of which irAEs can be effectively managed, especially when irAEs are recognized early and subjects are treated in a timely fashion. This can minimize the occurrence of irAE complications, such as GI perforation/colectomy or hepatic failure.

Since currently no cure for patients suffering from advanced melanoma is available and no drug ever has shown any survival benefit in prospective controlled randomized clinical phase III studies, ipilimumab at its 3mg/kg given four-times every 3 weeks is currently the best choice for melanoma patients who have failed systemic treatment in order to improve one- and two-year survival. Considering this background an irAE event rate of less than 25% grade 3-4 is highly acceptable.

In the context of the historical data which demonstrate a 2-year survival rate of approximately 8% to 18% in subjects with previously untreated advanced melanoma (Korn et al., 2008; Chapman et al., 1999; Middleton et al., 2000; Bedekian et al., 2006) 2-year survival rates for ipilimumab treated patients in clinical studies ranges from 29.81% to 41.78%.

The overall risk-benefit ratio for patients entering this protocol is therefore at least comparable to and possibly better than alternative options.

1.5 Study Rationale

Melanoma is the most common tumor of the skin that develops from a neoplastic transformation of melanocytes. The incidence of cutaneous melanoma is increasing at a faster rate than for any other solid tumor and is estimated as 68,000 new cases annually in the USA. Globally, the incidence of melanoma varies by region, and according to World Health Organization (WHO) over 130,000 new cases of melanoma are recognized annually around the world. Melanoma is a tumor with significant impact on society and when found to be metastatic, there is no effective treatments for most patients. Although most patients have localized disease at the time of diagnosis and are cured by surgical excision of the primary tumor, metastases can develop and most of these patients die of melanoma-associated causes. In the USA, over 8,000 deaths in 2008 were associated with melanoma.

Most melanomas develop in the skin (cutaneous melanoma). The AJCC classification is helpful in segregating risk for recurrence and survival in primary melanoma, especially when combined with the Breslow thickness criteria. The American Joint Committee on Cancer (AJCC) first published a staging schema in 1978 that incorporated primary tumor thickness, ulceration, level of invasion, regional and distant metastases subtypes and serum LDH levels that correlated with prognosis for relapse and survival (Balch et al J Clin Oncol 2009). For

metastatic melanoma, however, there is no current classification scheme that allows to predict a response to treatment in more than 25% in a given cohort. Melanomas from any anatomic site may metastasize. The overall median survival from diagnosis of stage IV melanoma has been estimated to be 8 months. One year survival for these patients has been reported as approximately 25%, with approximately 15% of patients surviving 5 years (SEER 2009). In metastatic melanoma, chemotherapy is used mostly with palliative intent. Currently registered agents for the treatment of melanoma include the alkylating agent dacarbazine (DTIC) and high dose IL-2 (US only). The administration of dacarbazine (DTIC) is a standard treatment with response rates in the range of 5-15% and progression free survival of approximately 8 weeks (Chapman et al, 1999). Cytotoxic therapies have not been reported to prolong overall survival.

This trial is designed in light of the high unmet medical need in the treatment of metastatic melanoma and the promising clinical results derived from trials with ipilimumab. Various phase II clinical studies using ipilimumab in unresectable stage III-IV have been conducted. Results of the first pivotal phase III study MDX010-20 were presented at ASCO 2010 (O´Day et al., JCO 2010) and published in parallel by Hodi et al. (NEJM, 2010). In the second ongoing phase III trial CA184-024, a different dose and treatment scheme is currently tested in the first-line setting. Results are expected at the end of 2010.

However, due to strictly defined inclusion and exclusion criteria in phase III trials, results of registrational trials might not answer all questions regarding safety and efficacy of ipilimumab in all advanced melanoma patient cohorts seen in daily routine. E.g. on one hand, in MDX010-20 trial, only patients positive for HLA-A2 could be enrolled (since patients were randomized in a double-blind fashion in a 1:3:1 ratio to receive HLA2-restricted melanoma gp 100 peptide vaccine monotherapy, MDX-010 in combination with melanoma gp100 peptide vaccine, or MDX-010 monotherapy.) In the second currently ongoing phase III study 184-024 patients were not restricted to HLAA2+ haplotype. However, a different patient group was enrolled (first line situation) and another dosing and treatment scheme was tested (combination with DTIC; ipilimumab10mg/kg and maintenance period following induction phase for patients who benefitted from ipilimumab). Patients with primary mucosal melanoma were excluded in CA184-024 study; patients with primary ocular melanoma were excluded in both Mdx010-20 as well as in CA184-024.

A large clinical phase II trial is proposed to treat broad range of metastatic melanoma patients who have failed at least one systemic treatment regimen in advanced, non-resectable melanoma. This study aims to gain more information about efficacy and safety of ipilimumab in a broad range of clinically relevant pretreated patient cohorts seen in daily routine, neither addressed in MDX-010-20 study nor in CA184-024. Enrollment will not be restricted to any specific HLA subtype and patients with uveal or ocular melanoma as well as cutaneous

melanoma patients with or without asymptomatic brain metastases can be treated with ipilimumab as well. Potential predictive markers (ALC, B-raf mutational status) will be prospectively evaluated. Wide access to ipilimumab in skin cancer-specialized clinical centers will be achieved in addition to a standardized evaluation of its clinical results and of the proposed management guidelines handling the side effects. Patients will be treated according the MDX010-20 protocol with ipilimumab monotherapy 3mg/kg four times during the induction phase only. Patients who benefit from therapy (stable disease of ≥ 3 months beginning at Week 12 or patients with initial partial or complete response at Week 12 assessment) and did not show any immune-related adverse event (irAE) ≥ grade 3 will be eligible for treatment re-induction in the case of melanoma relapse.

2 STUDY OBJECTIVES

2.1 Primary Objective

To further characterize efficacy of second line ipilimumab monotherapy 3mg/kg given according to the MDX010-20 protocol in a broad range of pretreated metastatic melanoma patients seen in daily clinical practice:

- Overall survival rate at 12 months defined as the rate of patients alive 12 months after the date from the first study treatment for complete study population

2.2 Secondary Objectives

To explore safety and additional efficacy parameters of the ipilimumab treatment in certain subgroups: (cutaneous versus uveal versus mucosal melanoma; LDH < 2 ULN versus LDH ≥ 2 ULN; status brain metastases versus no brain metastases in medical history; b-raf mutation positive versus b-raf mutation negative)

Secondary objectives are

- Safety / toxicity according to the CTC Criteria (Version 4.0)
- Efficacy according to immune-related response criteria (ir-RC) at any time during treatment
- Efficacy according RECIST criteria
- Progression free survival rate at 6 months
- Overall survival
- To explore clinical efficacy of ipilimumab in relation to b-raf mutation status, brain metastases and LDH

2.3 Exploratory Objectives

- To examine the value of peripheral blood absolute lymphocyte count (ALC) as a predictive biomarker in various patient cohorts with unresectable stage III-IV melanoma treated with ipilimumab monotherapy
-
- To evaluate possible surrogate markers in peripheral blood and tumour biopsy (translational research program)

3 STUDY DESIGN

3.1 Overall Study Design

This is an open-label, multi-center, single-arm clinical phase II study to further characterize the efficacy and safety of ipilimumab (3 mg/kg iv every 3 weeks over 10 weeks) in patients with pretreated, metastatic and/or inoperable melanoma. .

The study will have one final analysis. The final analysis will occur 12 months after the last patient has been entered the clinical study. Prior to closure of the study, it will be ensured that survival data has been collected for each subject for 1 year following the subject's first treatment dose. A Safety Data Monitoring Committee (SDMC) will be responsible for reviewing all Grade 3-4 irAE on a regular basis. Based on its review the DMSB will provide the sponsor with recommendation regarding trial modification, continuation or termination.

Figure 2 Study design

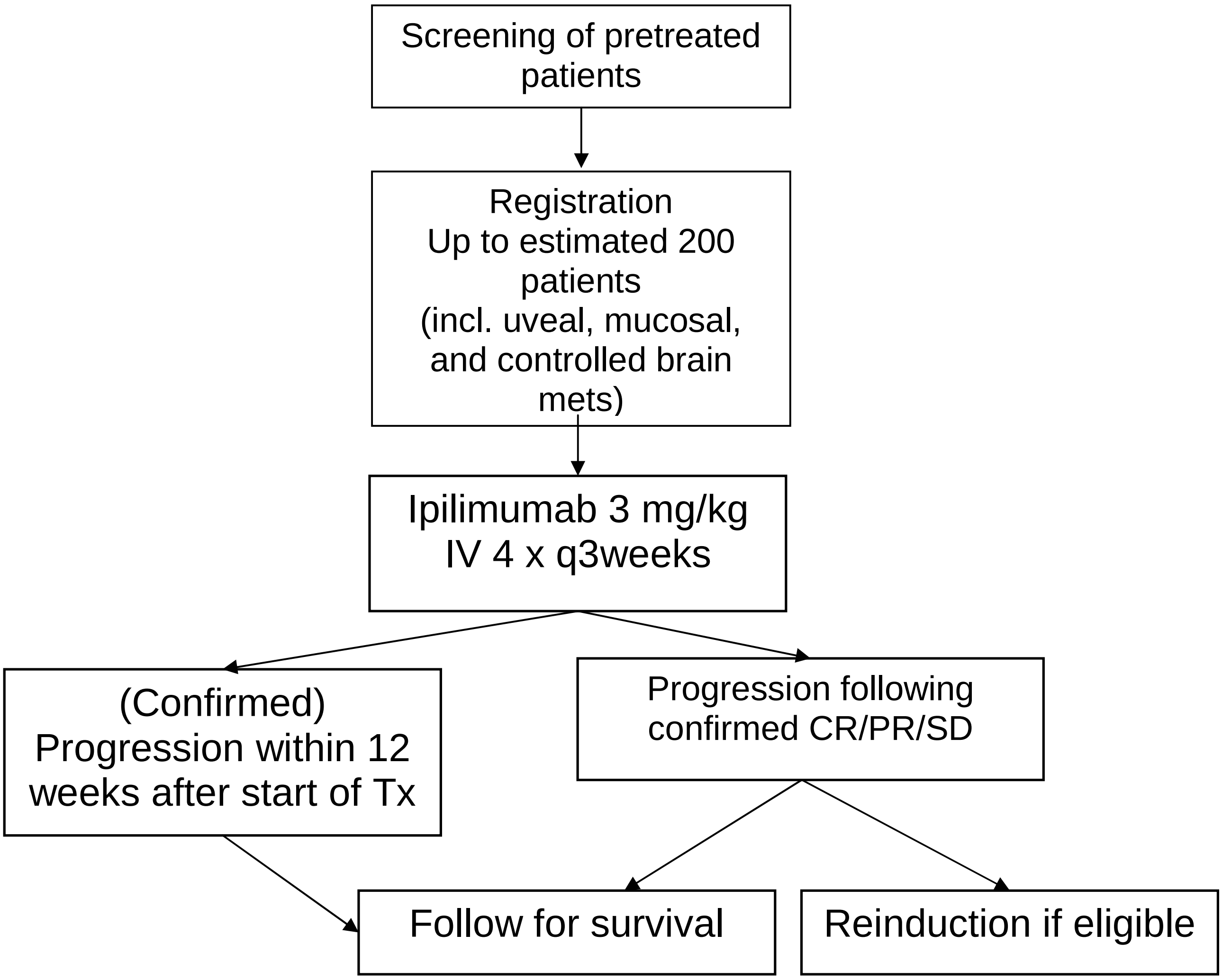

Tx = treatment with study medication

3.2 Enrollment of Patients

The selection of patients occurs through the investigator according to the inclusion and exclusion criteria after informing the patient written and orally about the study and after the patient has signed the informed consent. Patients considered eligible by the Investigator once all screening procedures are complete will be enrolled by online registration.

Online registration is possible 24 hours a day. After the registration form has been saved, the registration is notified immediately by an automatically generated fax to the investigator. The site should begin the patient on study treatment within 14 days of registration.

3.3 Number of Patients

The enrollment of up to 200 patients with unresectable stage III-IV melanoma is planned.

3.4 Timelines

Period of recruiting	8-9 months
Enrolment start date (FPI):	QII 2011
Enrolment finish date (LPI):	December 2011
End of study:	QIV 2012

3.5 Definition of the End of Study

End of study will be at recruitment finished plus 1 year post start of treatment of last patient thus ensuring that 1 year survival rate can be estimated. In case that the last patient will not start treatment, the trial will end 12 months after the last patient entered the trial. Therefore expected maximum study duration is 8-9 months accrual + 12 months for follow-up.

3.6 Plan for Treatment after the End of Study

Further patient treatment will be at the discretion of the Investigator. All patients in re-induction phase when their study period is ending, will be offered free ipilimumab to complete the re-induction treatment. Off-study patients eligible for a re-induction may be treated with ipilimumab if deemed appropriate by the treating physician, either using commercial drug if licensed in Germany or via the Early Access Program if the Marketing Authorization is not granted yet.

4 SUBJECT SELECTION CRITERIA

For entry into the study, the following criteria MUST be met. Any exceptions from the protocol-specific selection criteria must be approved by the Coordinating Investigator and/or the Institutional Review Board (IRB) before enrollment.

4.1 Inclusion Criteria

Patients meeting all of the following criteria will be considered for admission to the trial:

1. Histologically proven cutaneous, mucosal or uveal melanoma
2. Measurable disease according to RECIST in unresectable stage III-IV
3. Minimum age of 18 years,
4. Able and willing to give valid written inform consent
5. Must have received at least one line of prior systemic therapy for advanced malignant melanoma resulting in one of the following conditions:
   a. relapsed following a response of CR, PR or SD or
   b. failed to demonstrate a response of CR, PR or SD or
   c. did not tolerate such a regimen due to toxicity.
6. An interval of at least 28 days since treatment with chemotherapy, biochemotherapy, surgery, radiation, or immunotherapy, and recovery from any clinically significant toxicity experienced during treatment is recommended. Prior treatment must be completed by the time of ipilimumab administration. Palliative radiation therapy outside of the brain or therapeutic radiation to the brain after the patient's condition is stabilized and systemic steroids required for the management of symptoms due to brain metastases is decreased to the lowest fixed dose possible and does not require the 28-day waiting period. Patient must have recovered from any acute toxicity associated with prior therapy.
7. Expected survival of at least six months
8. ECOG Performance Status 0, 1 or 2.
9. Within the last 2 weeks prior to study day 1 the following laboratory parameters, which should be within the ranges specified:

Lab Parameter	Range
White blood cells (WBC)	≥ 2500/mm^3 (≥ 1 2.5 x 10^9/L)
Absolute neutrophil count (ANC)	≥ 1000/mm^3 (≥ 1.0 x 10^9/L)
Platelets	≥ 75.000/mm^3 (≥ 75 x 10^9/L)
Hemoglobin	≥ 9 g/dL (≥ 90 g/L; may be transfused)
Creatinine	≤ 2.0 x ULN
Bilirubine total	≤ 2.0 x ULN (excepted patients with Gilbert's Syndrome, who must have a total bilirubin less than 3.0 mg/dL)

ALT, AST	≤ 2.5 x ULN for patients without liver metastasis,<br>≤ 5 x ULN for patients with liver metastases

10. No childbearing potential or negative pregnancy test of women of childbearing potential performed within 7 days prior to the start of treatment.
Women of childbearing potential (WOCP) must be using an effective method of contraception (Pearl-Index < 1, e.g. oral contraceptives, other hormonal contraceptives [vaginal products, skin patches, or implanted or injectable products], or mechanical products such as an intrauterine device or barrier methods [diaphragm, spermicides]) throughout the study and for up to 26 weeks after the last dose of investigational product, in such a manner that the risk of pregnancy is minimized.
No men of fathering potential or men of fathering potential must be using an effective method of contraception to avoid conception throughout the study and for up to 26 weeks after the last dose of investigational product, in such a manner that the risk of pregnancy is minimized.

WOCBP include any female who has experienced menarche and who has not undergone successful surgical sterilization (hysterectomy, bilateral tubal ligation, or bilateral oophorectomy) or is not post-menopausal. Post-menopause is defined as:

- Amenorrhea ≥ 12 consecutive months without another cause, or
- For women with irregular menstrual periods and taking hormone replacement therapy (HRT), a documented serum follicle stimulating hormone (FSH) level ≥ 35 mIU/mL.

Women who are using oral contraceptives, other hormonal contraceptives (vaginal products, skin patches, or implanted or injectable products), or mechanical products such as an intrauterine device or barrier methods (diaphragm, condoms, spermicides) to prevent pregnancy, or are practicing abstinence or where their partner is sterile (eg, vasectomy) should be considered to be of childbearing potential.

WOCBP must have a negative serum or urine pregnancy test (minimum sensitivity 25 IU/L or equivalent units of HCG) at Baseline within 7 days before the start of ipilimumab and at week 12.

4.2 Exclusion Criteria

Patients will be excluded from the study for any of the following reasons:

1. The patient requires concomitant therapy with any of the following: IL-2, interferon, or other non-study immunotherapy regimens; cytotoxic chemotherapy; immunosuppressive agents; other investigation therapies; any other systemic therapy for cancer including any other experimental treatment.

2. The patient requires chronic use of systemic corticosteroids. Systemic steroids for management of symptoms due to brain mets should be avoided if possible or subject should be stable on the lowest clinically effective dose. Topical or inhalational steroids are permitted.
3. Use of any investigational or non-registered product (drug or vaccine) other than the study treatment.
4. Active autoimmune disease: Patients with a history of inflammatory bowel disease, including ulcerative colitis and Crohn's Diseasse, are excluded from this study, as are patients with a history of symptomatic disease (eg, rheumatoid arthritis, systemic progressive sclerosis [scleroderma], systemic lupus erythematosus, autoimmune vasculitis [eg, Wegener's Granulomatosis]); motor neuropathy considered of autoimmune origin (e.g. Guillain-Barre Syndrome and Myasthenia Gravis).
5. Symptomatic CNS metastases (Remark: Asymptomatic stable, untreated or pretreated central nervous system (CNS) metastasis are allowed)
6. Family history of congenital or hereditary immunodeficiency.
7. The patient is known to be positive for Human Immunodeficiency Virus (HIV) or other chronic infections (HBV, HCV) or has another confirmed or suspected immunosuppressive or immunodeficient condition.
8. The patient has psychiatric or addictive disorders that may compromise his/her ability to give informed consent or to comply with the trial procedures.
9. Lack of availability for clinical follow-up assessments.
10. The patient has concurrent severe medical problems, unrelated to the malignancy, that would significantly limit full compliance with the study or expose the patient to unacceptable risk.
11. Other serious illnesses, e.g., serious infections requiring antibiotics or bleeding disorders.
12. Patients with serious intercurrent illness, requiring hospitalization.
13. For female patients: the patient is pregnant or lactating. Women of childbearing potential: Refusal or inability to use effective means of contraception
14. Any underlying medical or psychiatric condition, which in the opinion of the investigator will make the administration of ipilimumab hazardous or obscure the interpretation of AEs, such as a condition associated with frequent diarrhea.
15. Subjects with melanoma who have another active, concurrent, malignant disease are not eligible for this trial, with the exception of adequately treated basal or squamous cell skin cancer, superficial bladder cancer, or carcinoma in situ of the cervix.

4.3 Justification of Gender

Within this clinical trial, no gender specific differences are expected concerning the efficacy and safety of the used study medication, so that it is not necessary to consider the gender during recruitment and evaluation or conception of this clinical trial. Therefore reasons according to §7 Abs. 2 Nr. 12 GCP V are not to apply.

5 STUDY THERAPY WITH IPILIMUMAB

5.1 Induction Treatment

Subjects enrolled after IC will receive ipilimumab according to the protocol of the completed Medarex study MDX010-20 (Hodi et al., NEJM, 2010) as a single agent at a dose of 3mg/kg for 4 times over 10 weeks (1 cycle): The day subjects receive the first dose of protocol ipilimumab therapy will define Day 1. Ipilimumab, 3 mg/kg, day 1 (W1), 22 (W4), 43 (W7), 64 (W10) must be administered intravenously over a 90-minute period every 3 weeks for a total of four doses, as tolerated. This dose demonstrated an overall survival benefit for the first time in treating metastatic melanoma in a controlled randomized clinical study (Hodi et al., NEJM, 2010).

– Electrocardiogram (ECG) examination should be performed during screening and week 12. Patients with an existing myocarditis are excluded from this study. For patients with suspicion of myocarditis e.g. dyspnoea, functional insufficiency etc. ECG examinations have to be performed before each treatment.
- Laboratory evaluations should be performed (refer to section 6.1.1.2) and the results examined before administration of each ipilimumab dose.
- As durable disease stabilization and/or objective tumor response can be seen after early progression before Week 12, it is recommended that, in the absence of dose-limiting toxicities (eg, serious irAEs), all four doses of ipilimumab be administered over the initial 12 weeks even in the setting of apparent clinical progression, providing the subject’s performance status remains stable.
- All subjects who enter the induction period, including those who may have discontinued treatment for drug-related AEs should obtain a 12-week, 24-week 36-week and 48 week tumor assessment. Those patients who have evidence of clinical progression during the induction period, should obtain further tumor assessment according to Standard of Care.
- Based on clinical experience in the ongoing and completed melanoma studies, the following recommendations apply for subject management in light of the Week 12 or later tumor assessments:

    - The appearance of new lesions in subjects with other stable or shrinking baseline tumor burden may be experiencing clinical benefit and should continue in follow-up before alternative anti-cancer agents are considered. These subjects can be seen to have continued tumor shrinkage in follow-up scans.
    - Clinical progression warranting alternative anti-cancer treatment should be considered only in subjects whose overall tumor burden appears to be

substantially increased and/or in subjects whose performance status is decreased.

5.2 Re-Induction Treatment

Patients who have progressed following stable disease (SD) of ≥ 3 months duration from Week 12 assessment OR patients who have progressed following an initial response to the induction treatment (CR or PR diagnosed at week 12 assessment may be offered additional cycles of therapy (4 infusions of ipilimumab 3mg/kg every 3 weeks) with the originally assigned treatment regimen (“Re-induction”) until off-treatment criteria are met, provided they meet re-treatment criteria specified below.

- Re-treatment criteria:

Patients who received ipilimumab as induction in this study and have met each of the following criteria within **28 days** before re-treatment, are eligible for “re-induction”:

• Had no unacceptable toxicity requiring ipilimumab discontinuation; OR
• Patients who did not permanently discontinue treatment due to select irAEs as follows:
− Reversible autoimmune hepatitis
− Medically manageable endocrinopathy
− Reversible dermatological toxicity
• ECOG performance status of 0, 1 or 2
• Electrocardiogram (ECG): during screening and week 12. Patients with an existing myocarditis are excluded from this study. For patients with suspicion of myocarditis e.g. dyspnoea, functional insufficiency etc. ECG examinations have to be performed before each treatment.

• Patient must meet all other inclusion criteria as outlined before (see Inclusion criteria)
AND
• Have experienced disease progression progression as documented by physical examination or diagnostic imaging (including brain imaging) after demonstrating expanded clinical benefit (see below).

All patients eligible for Re-induction treatment with ipilimumab their TSH level must be assessed at baseline and before each dose and the TSH level be available to the investigator prior to the administration of the dose. **The assessment of TSH is mandatory prior each treatment.** If a subject has thyroid dysfunction and concomitant symptoms (i.e. fatigue), the subject should be monitored more frequently and be treated as per standard medical practice.

The management algorithm for endocrine abnormalities is available on page 59 (appendix 10-15).

- Re-induction treatment:

During the Re-Induction treatment, subjects enrolled who are eligible for additional treatment cycles will repeat the treatment phase as outlined in INDUCTION TREATMENT. They will receive ipilimumab at the 3 mg/kg dose level every 3 weeks for the first 10 weeks (Weeks 1, 4, 7 and 10) of Re-induction.

Ipilimumab therapy should be offered to all subjects who have not experienced unacceptable toxicity (refractory Grade > 3 irAEs) and are considered by the investigator to be obtaining clinical benefit, either because of apparent tumor stability or continued shrinkage and/or late response. No patient will be re-treated if they experience a Grade 3 or higher nondermatologic autoimmune breakthrough event (ABE) considered related to study therapy or a Grade 3 or higher gastrointestinal or certain other immune-related adverse events (irAE) which require permanent discontinuation of ipilimumab (refer to section 5.5.3). If a patient has experienced a study medication-related dermatologic or non-dermatologic ABE < Grade 3, then the event must have improved to Grade 1 or less before re-treatment can be considered. No patient with disease progression following the first cycle of study medication will be permitted to be re-treated with study medication.

Patients can be eligible for more than one re-induction. The number of re-inductions is not limited. All re-inductions initiated while patient is on-study (***each patient study period,*** first dosing + 12 months) will be performed in the frame of the study. Any re-induction starting after each patient study period (first dosing + 12 months) would be done outside of the study.

5.3 Definition of Selected Reversible irAEs that May be Considered For Re-Treatment

Patients with selected irAEs related to ipilimumab that have completely resolved with immunosuppressive therapy or are adequately controlled with hormone therapy, may be considered for further re-treatment with ipilimumab under this study, at the time of disease progression. The list of completely reversible or medically managed immune-related adverse events (irAEs) eligible for consideration is:

- Reversible autoimmune hepatitis
- Medically managed endocrinopathy
- Reversible dermatological toxicity

All other irAEs are not considered eligible for re-treatment, including, but not limited to,

≥ Grade 3 colitis or diarrhea, or uveitis of any grade.

5.4 Dose Calculations

Calculate **Total Dose** as follows:

Patient body weight in kg x 3 mg = total dose in mg

Calculate **Total Infusion Volume** as follows:

Total dose in mg ÷ 5 mg/mL = infusion volume in mL

Calculate **Rate of Infusion** as follows:

Infusion volume in mL ÷ 90 minutes = rate of infusion in mL/min.

For example, a patient weighing 115 kg would be administered 345 mg of ipilimumab (115 kg x 3 mg/kg = 345 mg) with an infusion volume of 69 mL (345 mg ÷ 5 mg/mL = 69 mL). Since the final volume of the dose is less than 90 ml, add 0.9% saline to bring the final volume up to 90 ml. The infusion rate is 1.0 mL/min (90 mL ÷ 90 minutes)

5.5 Preparation, Administration, Dose Modification, Discontinuation

5.5.1 Preparation and Administration Guidelines

As with all injectable drugs, care should be taken when handling and preparing ipilimumab. Whenever possible, it should be prepared in a laminar flow hood or safety cabinet using standard precautions for the safe handling of intravenous agents, applying aseptic technique. Latex gloves are required. If ipilimumab concentrate or solution comes in contact with skin or mucosa, immediately and thoroughly wash with soap and water.

The supplies needed for ipilimumab preparation and administration include calibrated syringes and infusion containers. Ipilimumab is to be administered as an IV infusion using a volumetric pump through a 0.2 or 1.2 micrometer in-line filter (supplied by site) at the 3 mg/kg dose. See the current Investigator Brochure for additional information on allowable filter types. The infusion must be completed in 90 minutes with a 10 ml normal saline flush at the end. The ratio and rate will be specified in the pharmacy manual. The total dose must be calculated using the most recent subject weight (obtained within 3 days of the dosing visit, and prior to the infusion).

1. As ipilimumab is stored at refrigerated temperatures (2-8ºC), allow the appropriate number of vials of ipilimumab to stand at room temperature for approximately five minutes.
2. Aseptically withdraw the required volume of ipilimumab solution into a syringe. Insert the needle at an angle into the ipilimumab vial by placing the needle – bevel side down – against the glass, with the tip touching the neck of the vial. The initial solution

concentration is 5 mg/mL. [Note: A sufficient excess of ipilimumab is incorporated into each vial to account for withdrawal losses].

3. Ensure that the ipilimumab solution is clear colorless, essentially free from particulate matter on visual inspection. If multiple vials are needed for a subject, it is important to use a separate sterile syringe and needle for each vial to prevent problems such as dulling of needle tip, stopper coring, repeated friction of plunger against syringe barrel wall, etc.
4. Inject ipilimumab solution withdrawn into an appropriate size evacuated infusion bag to produce a final infusion volume that has been calculated from the weight of the patient. For example, if preparing a 3 mg/kg treatment for a 65 kg patient you will use 195 mg, the drug solution volume will be 39 mL total.
5. If the total dose calculates to less than 90 mL of solution then the total dose needed should be diluted to a total volume of 90 mL in 0.9% sodium chloride.
6. Mix by GENTLY inverting several times. DO NOT shake.
7. Visually inspect the final solution. If the initial diluted solution or final dilution for infusion is not clear or contents appear to contain precipitate, the solution should be discarded.
8. Do not draw into each vial more than once. Any partial vials should be safely discarded and should not be stored for reuse.
9. Ipilimumab should be administered under the supervision of a physician experienced in the use of intravenous (IV) agents. Ipilimumab is administered as an IV infusion only.

After final drug reconciliation, unused ipilimumab solution should be disposed of at the site following procedures for the disposal of anticancer drugs.

5.5.2 Dose Modifications

Dose de-escalation of ipilimumab from 3 mg/kg is not allowed.

Ipilimumab Dose Skipping Rule

Decisions to skip an ipilimumab dose must be made on specified safety criteria. Treatment with ipilimumab will be skipped or discontinued if the subject experiences at least one adverse event, specified below, considered by the investigator to be **"possibly", probably" or "certainly" related to ipilimumab treatment.** The investigator should contact the PI for any adverse event that will prompt a skipped dose or discontinuation of ipilimumab.

The following criteria will be used to determine dose skipping, restarting doses, or discontinuing ipilimumab.

It may be necessary to skip study drug dosing for the following related adverse event(s):

- Any ≥ Grade 2 non-skin related adverse event (including irAEs), except for laboratory abnormalities
- Any ≥ Grade 3 laboratory abnormality

- Any adverse event, laboratory abnormality or intercurrent illness that, in the judgment of the investigator, presents a substantial clinical risk to the subject with continued dosing.

It is necessary to skip study drug dosing for the following adverse events:

- Any ≥ Grade 3 skin related adverse event regardless of causuality.

Restart ipilimumab dosing if/when the adverse event(s) resolve(s) to ≤ Grade 1 severity or returns to baseline within 2 weeks of initial dose administration:

- If the *adverse event has resolved,* restart ipilimumab dosing at the next scheduled timepoint per protocol.
- If the adverse event has not resolved in the protocol-specified dosing window (3 weeks [±7 days], the next scheduled dose will be skipped and dosing will be resumed at the subsequently scheduled dose.
- If > 1 dose is expected to be skipped, the dosing schedule modifications must be discussed with the coordinating investigator prior to implementation.

5.5.3 Discontinuation of Study Therapy

Subjects MUST be discontinued from study therapy AND/ OR withdrawn from the study for the following reasons:

- Withdrawal of informed consent (subject's decision to withdraw for any reason)
- Any clinical adverse event, laboratory abnormality or intercurrent illness which, in the opinion of the investigator, indicates that continued treatment with study therapy is not in the best interest of the subject
- Clinical deterioration of subject's condition such that further benefit from ipilimumab dosing is unlikely or requires a change of therapy
- The development of progression (PD) in the global tumor burden (progression must be confirmed by serial imaging 4-6 weeks later)
- Pregnancy
  - All WOCBP should be instructed to contact the investigator immediately if they suspect they might be pregnant (e.g., missed or late menstrual period) at any time during study participation. Institutional policy and local regulations should determine the frequency of on study pregnancy tests for WOCBP enrolled in the study.
  - The investigator must immediately notify the coordinating investigator in the event of a confirmed pregnancy in a patient participating in the study.
- Termination of the study
- The compulsory detention for treatment of either a psychiatric or physical (e.g., infectious disease) illness

If study drug administration is discontinued, the reason for discontinuation will be recorded.

5.5.3.a Permanent Discontinuation for Related Adverse Events

The following treatment related non-neurological adverse events require permanent discontinuation of ipilimumab:

- Any ≥ Grade 2 eye pain or reduction of visual acuity that does not respond to topical therapy and does not improve to ≤ Grade 1 severity within 2 weeks of starting therapy, OR, requires systemic treatment.
- Any ≥ Grade 3 bronchospasm or other hypersensitivity reaction.
- Any other ≥ Grade 3 non-skin related adverse event with the exception of events listed under "No Discontinuation" (below).
  - Any ≥ Grade 4 laboratory abnormalities, except AST, ALT, or Total Bilirubin
  - AST or ALT > 8 x ULN
  - Total Bilirubin > 5 x ULN
  - Any other ≥ Grade 4 adverse event

- Any adverse event, laboratory abnormality or intercurrent illness which, in the judgment of the investigator, presents a substantial clinical risk to the patient with continued dosing.

Please refer to the IB for specific treatment algorithms.

The following neurological adverse event requires permanent discontinuation of ipilimumab and defines unacceptable neurotoxicity:

- Any motor neurologic toxicity >/= Grade 3 regardless of causality
- Any>/= Grade 3 treatment related sensory neurologic toxicity

Please refer to the IB for specific treatment algorithms.

5.5.3.b Exceptions to Permanent Discontinuation

- Potentially reversible inflammation (< Grade 4), attributable to a local anti-tumor reaction and a potential therapeutic response. This includes inflammatory reactions at sites of tumor resections or in draining lymph nodes, or at sites suspicious for, but not diagnostic of metastasis.
- Hospitalization for ≤ Grade 2 adverse events where the primary reason for hospitalization is to expedite the clinical work-up.
- Patients with the following conditions where in the investigator’s opinion continuing study drug administration is justified:
  - Ocular toxicity that has responded to topical therapy.
  - Endocrinopathies where clinical symptoms are controlled with appropriate hormone replacement therapy. **Note:** Ipilimumab may not be restarted while the patient is being treated with systemic corticosteroids except for patients on stable doses of hormone replacement therapy such as hydrocortisone.

5.5.4 Immune-Related Adverse Events (irAEs): Definition, Monitoring, and Treatment

Blocking CTLA-4 function may permit the emergence of auto-reactive T cells and resultant clinical autoimmunity. Rash/vitiligo, diarrhea/colitis, uveitis/episcleritis, hepatitis, and hypopituitarism were drug-related, presumptive autoimmune events, now termed irAEs, noted in previous ipilimumab studies.

For the purposes of this study, an irAE is defined as an AE of unknown etiology associated with drug exposure and consistent with an immune phenomenon. Efforts should be made to rule out neoplastic, infectious, metabolic, toxin or other etiologic causes prior to labeling an AE an irAE. Serological, immunological, and histological (biopsy) data should be used to support

the diagnosis of an immune-mediated toxicity. Suspected irAEs must be documented on an AE or SAE form.

Patients should be informed of and carefully monitored for evidence of clinically significant systemic irAE (e.g., systemic lupus erythematosus-like diseases) or organ-specific irAE (e.g., rash, colitis, uveitis, hepatitis or thyroid disease). If an irAE is noted, appropriate work-up (including biopsy if possible) should be performed, and steroid therapy may be considered if clinically necessary.

It is unknown if systemic corticosteroid therapy has an attenuating effect on ipilimumab activity. However, clinical anti-tumor responses have been maintained in patients treated with corticosteroids and discontinued from ipilimumab. If utilized, corticosteroid therapy should be individualized for each patient. Prior experience suggests that colitis manifested as ≥ Grade 3 diarrhea requires corticosteroid treatment.

Specific treatment algorithms for immune-related adverse events are included as appendices in the IB.

5.5.5 Other Guidance

5.5.5.a Treatment of Infusion Reactions Associated with Ipilimumab

Since ipilimumab contains only human protein sequences, it is less likely that any allergic reaction will be seen in patients. However, it is possible that infusion of ipilimumab will induce a cytokine release syndrome that could be evidenced by fever, chills, rigors, rash, pruritus, hypotension, hypertension, bronchospasm, or other symptoms. No prophylactic pre-medication will be given unless indicated by previous experience in an individual patient. Reactions should be treated based upon the following recommendations.

- For mild symptoms (e.g., localized cutaneous reactions such as mild pruritus, flushing, rash):
  - Decrease the rate of infusion until recovery from symptoms, remain at bedside and monitor patient.
  - Complete the ipilimumab infusion at the initial planned rate.
  - Diphenhydramine 50 mg IV may be administered at the discretion of the treating physician and patients may receive additional doses with close monitoring.
  - Premedication with diphenhydramine may be given at the discretion of the investigator for subsequent doses of ipilimumab.
- For moderate symptoms (any symptom not listed above [mild symptoms] or below [severe symptoms] such as generalized pruritus, flushing, rash, dyspnea, hypotension with systolic BP >80 mmHg):
  - Interrupt ipilimumab.

  - Administer diphenhydramine 50 mg IV.
  - Monitor patient closely until resolution of symptoms.
  - Corticosteroids may abrogate any beneficial immunologic effect, but may be administered at the discretion of the treating physician.
  - Resume ipilimumab infusion after recovery of symptoms.
  - At the discretion of the treating physician, ipilimumab infusion may be resumed at one half the initial infusion rate, then increased incrementally to the initial infusion rate.
  - If symptoms develop after resumption of the infusion, the infusion should be discontinued and no additional ipilimumab should be administered that day.
  - The next dose of ipilimumab will be administered at its next scheduled time and may be given with pre-medication (diphenhydramine and acetaminophen) and careful monitoring, following the same treatment guidelines outlined above.
  - At the discretion of the treating physician additional oral or IV antihistamine may be administered prior to dosing with ipilimumab.
- For severe symptoms (e.g., any reaction such as bronchospasm, generalized urticaria, systolic blood pressure <80 mm Hg, or angioedema):
  - Immediately discontinue infusion of ipilimumab, and disconnect infusion tubing from the subject.
  - Consider bronchodilators, epinephrine 1 mg IV or subcutaneously, and/or diphenhydramine 50 mg IV, with solumedrol 100 mg IV, as needed.
  - Patients should be monitored until the investigator is comfortable that the symptoms will not recur.
  - No further ipilimumab will be administered.
- In case of late-occurring hypersensitivity symptoms (e.g., appearance within one week after treatment of a localized or generalized pruritus), symptomatic treatment may be given (e.g., oral antihistamine, or corticosteroids).

5.5.5.b Treatment of Ipilimumab-Related Isolated Drug Fever

In the event of isolated drug fever, the investigator must use clinical judgment to determine if the fever is related to the ipilimumab or to an infectious etiology. If a patient experiences isolated drug fever, for the next dose, pre-treatment with acetaminophen or non-steroidal anti-inflammatory agent (investigator discretion) should be instituted and a repeated antipyretic dose at 6 and 12 hours after ipilimumab infusion, should be administered. The infusion rate will remain unchanged for future doses. If a patient experiences recurrent isolated drug fever following premedication and post dosing with an appropriate antipyretic, the infusion rate for subsequent dosing should be decreased to 50% of the previous rate. If fever recurs following infusion rate change, the investigator should assess the patient’s level of discomfort with the event and use clinical judgment to determine if the patient should receive further ipilimumab.

5.5.5.c Liver Function Tests (LFT) Assessments Required Prior to Administration of ipilimumab

Liver function tests (AST, ALT, T. bilirubin) will be evaluated for every subject prior to administration of ipilimumab. Blood samples must be collected and analyzed at local or central labs within 3 days prior to dosing. LFT results must be reviewed by the principal investigator (or designee) to meet dosing criteria specifications: ≤ 2.5 x ULN for AST, ALT and ≤ 1.5 x ULN for T. bilirubin unless liver metastases are present in which case LFT ≤ 5 x ULN for AST, ALT and T. bilirubin ≤ 3.0 x ULN) prior to dosing.

If, during the course of treatment abnormal LFT values are detected, the subject must be managed using the hepatotoxicity algorithm section of the ipilimumab Investigators.

5.6 Prohibited and Restricted Therapies During the Study

5.6.1 Prohibited Therapies

Patients in this study may not use vaccines for the treatment of cancer or prevention of disease (including those for common medical conditions) for up to one month pre and post dosing with ipilimumab. Concomitant systemic or local anti-cancer medications or treatments are prohibited in this study while receiving ipilimumab treatments.

Patients may not use any of the following therapies during the study:

- Any non-study anti-cancer agent (investigational or non-investigational)
- Any other investigational agents
- Any other (non-CA184024 related) CTLA-4 inhibitors or agonists
- CD137 agonists
- Immunosuppressive agents
- Chronic systemic corticosteroids
- Any non-oncology vaccine therapies used for the prevention of infectious diseases (for up to 30 days prior to or after any dose of study drug).

5.6.2 Restricted Therapies

“Not applicable.”

6. STUDY PROCEDURES AND OBSERVATIONS

6.1. Procedures by Visit

6.1.1. Study Procedures by Visit and Treatment Cycle

Note that results of all safety laboratory tests (that is, all chemistry and all hematology results) must be obtained and reviewed before ipilimumab administration, as applicable. All laboratory results must be within the established range before ipilimumab is administered. All induction period laboratory samples must be collected within a window of up to 3 days before administration of ipilimumab. Laboratory evaluations using a local laboratory must be performed and the result examined by the investigator before administration of each dose of ipilimumab.

The disease will be assessed at baseline, after 12 weeks and for patients with stable disease or better responses, thereafter every 12 weeks in the absence of PD to a maximum of one year. Response evaluation will be done according to RECIST and immune-related response criteria (1).

All patients who prematurely discontinue treatment due to a drug-related adverse event prior to Week 12 (in the absence of disease progression) will return for all study visits and procedures including Week 12 and, if appropriate, further re-staging assessments until disease progression is observed.

– Subjects who are no longer receiving ipilimumab because of unacceptable toxicity (refractory Grade ≥ 3 irAEs) or due to investigator judgment are followed with tumour assessment until ir PD. Efficacy assessments for these subjects during follow-up are as per the standard of care. Date of death is recorded.

– Subjects who discontinue ipilimumab treatments are followed with tumour assessment until ir PD and should be followed until death or the closure of the study (whichever is first).

– Subjects who are no longer receiving ipilimumab because of progression and who have switched to alternative treatment are not followed formally except to record the date of death.

Any patient with documented progression at any scheduled re-staging visit who is not eligible for re-induction treatment will undergo no further re-staging visits and enters the Follow up phase (FU phase).

At the time of closure of the study (LPFV+12 months), it will be ensured that survival status (alive/dead and date) has been collected for each subject for each subject's full follow-up period (i.e: for 1 year from the subject's first treatment dose).

6.1.1.1. Screening/Baseline Visit

At the screening/baseline visit, the patient will consent to the study and eligibility will be determined according to the inclusion/exclusion criteria as detailed in Section 4. Patients must meet all inclusion criteria and none of the exclusion criteria to be considered eligible. In addition, a full medical history, prior and ongoing antineoplastic therapies, concurrent medications, and demographic information will be collected. Baseline CT/MRI and photographic imaging will take place at this visit. The following tests and examinations must be performed and the eligibility criteria satisfied before treatment may begin:

All patients participating in this clinical study with ipilimumab their TSH level must be assessed at baseline and before each dose and the TSH level be available to the investigator prior to the administration of the dose. The assessment of TSH is mandatory prior each treatment.

- Histologic diagnosis of malignant melanoma, B-RAF status of tumor biopsy
- Demographics and medical history
- Measurements of weight, height, temperature, pulse, respiratory rate, and resting systolic and diastolic blood pressure
- Complete physical examination
- ECOG Performance Status
- Current medications
- Baseline signs and symptoms
- Urine pregnancy test for WOCBP.
- Electrocardiogram (ECG): Patients with an existing myocarditis are excluded from this study.
- Baseline MRT/CT brain and MRT/CT chest/abdomen/pelvis scan was performed. The images can be accepted if obtained 6 weeks before initiation of ipilimumab
- Clinical laboratory tests:
  - Hematology: hemoglobin, CBC counts with differential (including absolute lymphocyte count) and direct platelet count.
  - Serum Chemistry: albumin, CRP, SGOT (AST)/SGPT (ALT), bilirubin (direct and total), creatinine, lactate dehydrogenase (LDH),lipase, electrolytes (including sodium, potassium, chloride, and bicarbonate) TSH, Free T4, S-100
  - Urinalysis (urine dipstick for protein)
- B-Raf Mutational Status and Microtissue Array:

Currently nothing is known whether response to ipilimumab might also be modulated by b-raf mutational status. Therefore Formalin Fixed Paraffin Embedded (FFPE) tissue is requested from every patient. FFPEs blocks from histologically confirmed cutaneous melanoma such as. Primary tumor or subsequent metastases such as lymph node etc. will be eligable.

DNA will be extracted and analysed for B-raf V600E mutational status by PCR. Furthermore, tissue micro arrays (TMAs) will be constructed to investigate tumor microenvironment and tumor infiltrating immune cells

6.1.1.2. Treatment Visits (Visits 2 through 5 + End of treatment visit)

Tumor assessments should be performed until disease progression is observed, both for induction and re-induction.

Induction Treatment

Subjects are required to have blood collected and chemistry labs analyzed up to 72 hours prior to each dose of ipilimumab. Investigators or an assigned medical professional must review liver function tests from the predosing labs and ensure the results meet dosing requirements prior to authorizing administration of each dose of ipilimumab.

During this treatment phase, the following procedures will be performed within +/- 7 days of each scheduled visit:

- Vital sign measurements will include weight, temperature, pulse, and resting systolic and diastolic blood pressure. Vital sign measurements of pulse, and systolic and diastolic blood pressure will be collected every 30 minutes for the duration of the ipilimumab, 1 hour following the completion of the infusion, and routinely unless otherwise indicated
- Physical examination
- ECOG Performance Status
- ECG: Patients with evident of myocarditis are excluded. Patients with suspicion of myocarditis (e.g. dyspnoea, functional insufficiency etc.) ECG examinations have to be performed before each treatment.
- Clinical laboratory tests (as outlined in the screening phase) must be analyzed within 3 days prior to dosing (results reviewed prior to study drug administration):

All patients participating in this clinical study with ipilimumab their TSH level must be assessed at baseline and before each dose and the TSH level be available to the investigator prior to the administration of the dose. **The assessment of TSH is mandatory prior each treatment.** If a subject has thyroid dysfunction and concomitant symptoms (i.e. fatigue), the subject should be

monitored more frequently and be treated as per standard medical practice. The management algorithm for endocrine abnormalities is available on page 59 (appendix 10-15).

- − Hematology
- − Serum chemistry
- − TSH, Free T4 (Visits 2 -5 [prior to study drug administration])
- – LDH, S-100 (Visit 2, 3, 5)
- − Urinalysis (urine dipstick for protein)

- • Assessment of signs and symptoms
- • Urine pregnancy test for WOCBP (not at end of treatment visit)
- • Concomitant medications
- • Adverse event assessment

Re-induction Treatment

For all subjects, during the Re-Induction Phase, the following procedures must be performed within 3 days of each scheduled visit and recorded in the CRF at the scheduled visit:

- • Urine pregnancy test for WOCBP
- • Vital sign measurements will include weight, temperature, pulse, and resting systolic and diastolic blood pressure. Vital sign measurements of pulse, and systolic and diastolic blood pressure will be collected every 30 minutes for the duration of the ipilimumab, 1 hour following the completion of the infusion, and routinely unless otherwise indicated.
- • Physical examination
- • ECOG Performance Status
- • ECG (baseline and week 12): Patients with evident of myocarditis are excluded. Patients with suspicion of myocarditis (e.g. dyspnoea, functional insufficiency etc) ECG examinations have to be performed before each treatment
- • Clinical laboratory tests (as outlined in the screening phase) (taken and results reviewed prior to study drug administration):

All patients participating in this clinical study with ipilimumab their TSH level must be assessed at baseline and before each dose and the TSH level be available to the investigator prior to the administration of the dose. **The assessment of TSH is mandatory prior each treatment.** If a subject has thyroid dysfunction and concomitant symptoms (i.e. fatigue), the subject should be monitored more frequently and be treated as per standard medical practice. The management algorithm for endocrine abnormalities is available on page 59 (appendix 10-15).

- − Hematology
- − Serum chemistry
- − TSH, Free T4 (Visits 2 and 4 [prior to study drug administration])
- – LDH, S-100 (Visit 2, 3, 5)
- − Urinalysis (urine dipstick for protein)

• Assessment of signs and symptoms
• Concomitant medications
• Adverse event assessment

All patients who prematurely discontinue treatment due to a drug-related adverse event prior to Week 12 (in the absence of disease progression) will return for all study visits and procedures including Week 12 and, if appropriate, further re-staging assessments until disease progression is observed.

6.1.1.3. Restaging-Visits

Restaging-visits will be invited for all patients in a 12-week intervall who have potentially benefitted from treatment. All patients should undergo radiographic tumor assessments every 12 weeks until disease progression is observed, both for induction and re-induction. Restaging-visits will be perforemd for each subject during a maximum of 1 year following first treatment dose. The End of Study visit should be performed when tumor follow-up on the patient will no longer continue, regardless of whether the patient progressed or not.

The following procedures will be performed within of each scheduled restaging

- Measurements of vital signs (resting systolic and diastolic blood pressure, pulse, respiratory rate, and temperature) and weight
- Physical examination
- ECOG Performance Status
- ECG at week 12 only
- Clinical laboratory tests
  - Hematology
  - Serum chemistry
  - TSH, Free T4
- LDH, S-100 (Visit 5/ week 12)
  - Urinalysis (urine dipstick for protein)
- Assessment of signs and symptoms
- Concomitant medications
- Adverse event assessment.

Study Completion or Early Discontinuation Visit

At the time of study early withdrawal, the reason for early withdrawal and any new or continuing adverse events should be documented.

6.1.1.4. End of Treatment Visit

The end of treatment visit will be performed 10 weeks after last administration of study therapy. The following procedures will be performed during end of treatment visit:

- Measurements of vital signs (resting systolic and diastolic blood pressure, pulse, respiratory rate, and temperature) and weight
- Physical examination
- ECOG Performance Status
- Clinical laboratory tests
  - Hematology
  - Serum chemistry
  - TSH, Free T4
  - Urinalysis (urine dipstick for protein)
- Assessment of signs and symptoms

6.1.1.5. FU-Phase

Patients who have progressed initially and were not eligable for re-induction are followed for survival only on a regular basis e.g. 3 months intervals by phone until death, the trial ends or the patient becomes lost-to-follow-up (LTFU). At the time of closure of the study (LPFV+12 months), it will be ensured that survival status (alive/dead and date) has been collected for each subject for each subject's full follow-up period (i.e: for 1 year from the subject's first treatment dose).

6.2. Safety Assessments

All patients who receive at least one dose of ipilimumab will be considered evaluable for safety parameters. Additionally, any occurrence of nonserious or SAE from time of consent forward, up to and including follow-up visits, will be reported. See Section 8: Adverse Event Reporting.

Safety will be evaluated for all treated patients using the National Cancer Institute (NCI) Common Terminology Criteria for Adverse Events (CTCAE), Version 4.0 (http://www.acrin.org/Portals/0/Administration/Regulatory/CTCAE_4.02_2009-09-15_QuickReference_5x7.pdf). Safety assessments will be based on medical review of adverse event reports and the results of vital sign measurements, physical examinations, and clinical laboratory tests.

Appendix 10 to 15 presents algorithm for safety and provides recommendations for immunerelated adverse events. The algorithm should help to inform and enhance patient care and reduce the risk of immunerelated AEs and to initiate therapy and minimize complications.

Immunerelated AEs are generally mangeable using symptomatic or immunosuppressive therapy as recommended through detailed diagnosis and management guidelines as described in the investigatiors´ Brochure (IB version no: 13 dated 26-Aug-2010). The management guidelines for general immune-related AEs and ipilimumab-related gastrointestinal toxicities, hepatotoxicity, endocrinopathy and neuropathy are provided in the IB Appendices 6,7,8,9, and 10 respectively. In addition if necessary please contact the study core center.

6.3. Stopping Rules

The trial can be prematurely closed or suspended by the sponsor in following cases:

- If ≥ 50% of treated patients present an AE ≥ grade III which is at least possibly related to ipilimumab
- If ≥ 20% of treated patients experience an unexpected at least possibly related AE which is not be alleviated or controlled by appropriate treatment and/or systemic steroids and/or infliximab within 14 days after initiation of such therapy
- If any ipilimumab-related death occurred unless also attributed to disease progression

In case of occurrence of any of the above listed situations the accrual will be suspended if appropriate until the sponsor has reviewed the events together with BMS and determined the appropriate course of action which could include the discontinuation of the trail, a protocol amendment or trial continuation as started. No efficacy data will be analyzed at this time point. Patients who are already on treatment will be informed individually if stopping rules apply based on the interim safety analysis. It will be individually decided together with each patient, whether further treatment according to protocol is applied.

6.4. Criteria for Evaluation

6.4.1. Safety Evaluation

All subjects who receive study drug will be monitored for safety. Study drug adverse effects will be assessed continuously. Adverse events and other symptoms will be graded according to the National Cancer Institute Common Terminology Criteria for Adverse Events, Version 4.0 (Appendix 8: *http://ctep.cancer.gov).* All serious adverse events and adverse events regardless of study drug attribution will be recorded on the case report form and analyzed.

Each serious adverse event must be documented on the electronically available "**Serious adverse events report**" form. The completed form is automatically faxed (fax no. is filed automatically in the system) to the sponsor and Alcedis GmbH. In the event that electronic reporting is not possible, paper SAE forms in the investigator's file handed out at the beginning of the study are at the doctor's disposal for notification by conventional fax. An SAE report should be completed for any event where doubt exists regarding its status of seriousness. The collection of SAEs should begin following the subject's written consent to participate in the study. SAEs include any laboratory test result that meets the definition of an SAE. Adverse events leading to discontinuation from treatment must be documented on the electronically available “Adverse event report” form. These events also include any laboratory abnormality that required the subject to have ipilimumab discontinued.

6.4.2. Efficacy Evaluation

6.4.2.1. Definition of Measurable/Non-Measurable and Index/Non-Index Lesions

Definitions of lesions are based on *RECIST criteria and immune-related response criteria (irRC)* in this study. For the evaluation of the therapeutic response in the study only the RECIST guidelines will be applied. The lesions evaluation by using irRC will be performed simultaneously.

6.4.2.2. Definition of Measurable and Non-Measurable Lesions

- Measurable lesions are lesions that can be accurately measured in at least one dimension with longest diameter at least 20 mm using conventional X-ray, CT or MRI techniques, or at least 10 mm with spiral CT scan. Skin lesions can be considered measurable.
- **Non-Measurable (evaluable) Lesions** are all other lesions, including unidimensionally measurable disease and small lesions (lesions without at least one diameter ≥ 20 mm), and any of the following:
  - Lesions occurring in a previously irradiated area (unless they are documented as new lesions since the completion of radiation therapy), bone lesions, leptomeningeal disease, ascites, pleural or pericardial effusion, lymphangitis cutis/pulmonis, abdominal

masses that are not pathologically/cytologically confirmed and followed by imaging techniques and cystic lesions.

– All measurable and non-measurable lesions should be measured at screening and at the defined tumor assessment timepoints (every 12 weeks).

6.4.2.3. Definition of Index/Non-Index Lesions

All measurable lesions, up to a maximum of **two lesions per organ** and **five lesions in total,** should be identified as *index* lesions to be measured and recorded on the medical record at Screening. The *index* lesions should be representative of all involved organs. In addition, *index* lesions should be selected based on their size (lesions with the longest diameters), their suitability for accurate repeat assessment by imaging techniques, and how representative they are of the patient's tumor burden. At Screening, a sum of the products of diameters (SPD) for all *index* lesions will be calculated and considered the baseline sum of the products of diameters. Response criteria to be followed are listed below. The baseline sum will be used as the reference point to determine the objective tumor response of the *index* lesions at tumor assessment (TA).

Measurable lesions, other than *index* lesions, and all sites of non-measurable disease, will be identified as *non-index* lesions. *Non-index* lesions will be recorded on the medical record and should be evaluated at the same assessment time points as the *index* lesions. In subsequent assessments, *non-index* lesions will be recorded as "stable or decreased disease," "absent," or "progression."

6.4.3. Evaluation of Tumor Response Using RECIST Criteria

6.4.3.1. Definition of Measurable Lesion Response Using RECIST Criteria

Measurable lesion will be measured in a dimension using the longest diameter is used. The tumour response will be investigated during every re-staging visit.

<u>Complete response (CR):</u> Disappearance of all lesions and appearance of no new lesions.

<u>Partial response (PR):</u> At least a 30% decrease in the sum of the longest diameter of measurable lesions. No new lesions may occur or individual lesions progress.

<u>Progressive disease (PD):</u> At least a 20% increase in the total of the longest diameters of measurable lesions (as reference to the smallest sum recorded since the treatment started) or the appearance of new lesions.

If the measurable lesion disappears and the progression of a lesion or the appearance of new lesions is observed elsewhere at the same time, this is also documented as a progressive disease.

<u>Stable disease (SD):</u> Neither a partial nor a complete response in the absence of progression.

6.4.3.2. Definition of Non-Measurable Lesion Response Using RECIST Criteria

- **Complete Response:** Complete disappearance of all *non-measurable* lesions.
- **Stable Disease:** A decrease or tumor stabilization of one or more *non-measurable* lesions. Subject with PR or CR that is not confirmed after at least 4 weeks are scored as SD unless they have new primary lesions.
- **Progressive Disease:** Progression of *non-measurable* lesion(s) (e.g., an increase in pleural effusions, or other fluid collections defined as an approximate doubling of the volume which was present at baseline or nadir, unless there is radiographic evidence of a benign cause for the fluid collection or the effusion is cytologically negative for malignant cells).

6.4.3.3. Determination of Overall Response Using RECIST Criteria

Overall Response (OR) is determined as the combination of assessments of *measurable* and *non-measurable* lesions using the following criteria:

Measurable lesions	***Non- measurable lesions***	***New lesions***	***Overall response***
CR	***CR***	***No***	***CR***
CR	***SD***	**No**	**PR**
**PR**	**SD**	**No**	**PR**
**SD**	**SD**	**No**	**SD**
**PD**	**any**	**Yes or No**	**PD**
**Any**	**PD**	**Yes or No**	**PD**
**Any**	**any**	**Yes**	**PD**

Best OR is the best confirmed response designation over the study as a whole, recorded between the date of first dose until the last tumor assessment for the individual patient in the study. The assessment of response at 12 weeks has particular emphasis due to the mechanism of action of ipilimumab inducing immune responses as basis for clinical responses. For the assessment of best OR, all available assessments per patient are considered. CR or PR determinations included in the best OR assessment must be confirmed by a second (confirmatory) evaluation meeting the criteria for response and performed no less than 4 weeks after the criteria for response are first met.

Imaging of the brain by MRT or CT, chest, abdomen and pelvis by CT scan is required at screening (ie, baseline) and at each tumor assessment visit in 12-week intervals, regardless of the location of known metastases. Similar methods of tumor assessment and similar techniques (CT or MRT) must be used to characterize each identified and reported lesion at screening and during subsequent tumor assessments. Imaging-based evaluation is preferred to clinical examination.

If progressive disease is assessed based only on a new lesion(s) found on bone scans, additional imaging studies of the lesion(s) should be performed to confirm the malignant nature of the new findings on the bone scan. Increased intensity of uptake in previously abnormal areas on bone scans is not considered progressive disease, unless the lesions seen on the correlative imaging studies performed of this area meet the criteria for progression. New areas of abnormal uptake on a bone scan represent progressive disease.

6.4.4. Definition of Tumor Response Using irRC

The sum of the products of diameters at tumor assessment using the immune-related response criteria (irRC) for progressive disease incorporates the contribution of new measurable lesions. Each net Percentage Change in Tumor Burden per assessment using irRC criteria accounts for the size and growth kinetics of both old and new lesions as they appear.

6.4.4.1. Definition of Index Lesions Response Using irRC

- **irComplete Response (irCR):** Complete disappearance of all *index* lesions. This category encompasses exactly the same subjects as “CR” by the mWHO criteria.
- **irPartial Response (irPR):** Decrease, relative to baseline, of 50% or greater in the sum of the products of the two largest perpendicular diameters of all *index* and all new measurable lesions (ie., Percentage Change in Tumor Burden). Note: the appearance of new measurable lesions is factored into the overall tumor burden, but does not automatically qualify as progressive disease until the SPD increases by ≥25% when compared to SPD at nadir.
- **irStable Disease (irSD):** Does not meet criteria for irCR or irPR, in the absence of progressive disease.
- **irProgressive Disease (irPD):** At least 25% increase Percentage Change in Tumor Burden (i.e., taking sum of the products of all *index* lesions and any new lesions) when compared to SPD at nadir.

6.4.4.2. Definition of Non-Index Lesions Response Using irRC

- **irComplete Response (irCR):** Complete disappearance of all *non-index* lesions. This category encompasses exactly the same subjects as “CR” by the mWHO criteria.
- **irPartial Response (irPR) or irStable Disease (irSD):** *non-index* lesion(s) are not considered in the definition of PR, these terms do not apply.
- **irProgressive Disease (irPD):** Increases in number or size of *non-index* lesion(s) does not constitute progressive disease unless/until the Percentage Change in Tumor Burden increases by 25% (i.e., the SPD at nadir of the index lesions increases by the required amount).

6.4.4.3. Impact of New Lesions on irRC

New lesions in and by themselves do not qualify as progressive disease. However their contribution to total tumor burden is included in the SPD which in turn feeds into the irRC criteria for tumor response. Therefore, new non-measurable lesions will not discontinue any subject from the study.

6.4.4.4. Definition of Overall Response Using irRC

Overall response using irRC will be based on these criteria:

- **Immune-Related Complete Response (irCR):** Complete disappearance of *all* tumor lesions (index and nonindex together with no new measurable/unmeasurable lesions) for at least 4 weeks from the date of documentation of complete response.
- **Immune-Related Partial Response (irPR):** The sum of the products of the two largest perpendicular diameters of all index lesions is measured and captured as the SPD baseline. At each subsequent tumor assessment, the sum of the products of the two largest perpendicular diameters of all index lesions and of new measurable lesions are added together to provide the Immune Response Sum of Product Diameters (irSPD). A decrease, relative to baseline of the irSPD compared to the previous SPD baseline, of 50% or greater is considered an immune Partial Response (irPR).
- **Immune-Related Stable Disease (irSD):** irSD is defined as the failure to meet criteria for immune complete response or immune partial response, in the absence of progressive disease.
- **Immune-Related Progressive Disease (irPD):** It is recommended in difficult cases to confirm PD by serial imaging. Any of the following will constitute progressive disease:
  - At least 25% increase in the sum of the products of all index lesions over baseline SPD calculated for the index lesions.
  - At least a 25% increase in the sum of the products of all index lesions and new measurable lesions (irSPD) over the baseline SPD calculated for the index lesions.

Table 3: Immune-Related Response Criteria Definitions

Index Lesion Definition	Non-Index Lesion Definition	New Measurable Lesions	New Unmeasurable Lesions	Percent change in tumor burden (including measurable new lesions when present)	Overall irRC Response
Complete Response	Complete Response	No	No	-100%	irCR
Partial Response	Any	Any	Any	≥ -50%	irPR
				<-50% to <+25%	irSD

Index Lesion Definition	Non-Index Lesion Definition	New Measurable Lesions	New Unmeasurable Lesions	Percent change in tumor burden (including measurable new lesions when present)	Overall irRC Response
				>+25%	irPD
Stable Disease	Any	Any	Any	<-50% to <+25%	irSD
				>+25%	irPD
Progressive Disease	Any	Any	Any	>+25%	irPD

6.4.4.5. Immune-Related Best Overall Response Using irRC (irBOR)

irBOR is the best confirmed irRC overall response over the study as a whole, recorded between the date of first dose until the last tumor assessment before subsequent therapy (except for local palliative radiotherapy for painful bone lesions) for the individual subject in the study. For the assessment of irBOR, all available assessments per subject are considered.

irCR or irPR determinations included in the irBOR assessment must be confirmed by a second (confirmatory) evaluation meeting the criteria for response and performed no less than 4 weeks after the criteria for response are first met.

6.4.5. Response Endpoints

Ipilimumab is expected to trigger immune-mediated responses, which require activation of the immune system prior to the observation of clinical responses. Such immune activation may take weeks to months to be evident. Some patients may have objective volume increase of tumor lesions or other disease parameters (based on study indication, ie, hematologic malignancies) within 12 weeks following start of ipilimumab dosing. Such patients may not have had sufficient time to develop the required immune activation or, in some patients, tumor volume or other disease parameter increases may represent infiltration of lymphocytes into the original tumor or blood. In conventional studies, such tumor volume or relevant laboratory parameter increases during the first 12 weeks of the study would constitute PD and lead to discontinuation of imaging to detect response, thus disregarding the potential for subsequent immune-mediated clinical response.

Therefore, patients with tumor volume increase detected or lack of laboratory parameter response documentation prior to week 12 but without rapid clinical deterioration should

continue to be treated with ipilimumab and clinically observed with a stringent imaging schedule to allow detection of a subsequent tumor response. This will improve the overall assessment of the clinical activity of ipilimumab and more likely capture its true potential to induce clinical responses. Tumor assessments will be made using immune-related response criteria and RECIST criteria.

6.5. Translational Research Programm

Since ipilimumab is a T-cell potentiator it will most likely exert its effects on lymphocytes and other immune cells. Early clinical data suggest a correlation between early increase in ALC with clinical benefit after ipilimumab treatment (Yang et al., 2010; Ku et al., 2010). Furthermore, targeted therapy using agents such as PLX4032 are dependent in its efficacy on the presence of specific b-raf mutations. Based on requested blood samples and tumour tissue the following side studies are planned.

6.5.1. Soluble Factors in Patients´ Serum

Analysis of various soluble factors such as LDH, S-100, IL-8, IL-6; TNF, sFas, bFGF, S-100, VEGF using Multiplex protein technologies in sera of all patients **before** start of treatment and **time point of first evaluation (week 12)**. In context, **20 ml venous blood** will be collected once at the beginning of the study and thereafter at week 12, respectively. Levels will be compared with response and outcome.

- 2 tubes of 10 ml whole venous blood. Samples will need to be processed for serum and plasma and stored at -70 ℃ until collection vi a central laboratory.

Sample processing for serum:

- Draw tubes of 10 ml whole blood in Corvac Serum Separator Tube (Sherwood Medical, St.Louis, MO)
- Allow to coagulate for 1 hour at room temperature.
- Centrifuge to separate clot from serum.
- Divide serum in at least 8 aliquots (approximately 1 ml each) in 1.8 ml cryotubes (Nunc or equivalent).
- Store at -70 ℃ until collection via central labor atory.

Sample processing for plasma:

- Draw tubes of 10 ml whole blood in Corvac Plasma Separator Tube (Sherwood Medical, St.Louis, MO). Tube contains lithium-heparin
- Allow for plasma separation for 1 hour at room temperature.

- Divide plasma in at least 8 aliquots (approximately 1 ml each) in 1.8 ml cryotubes (Nunc or equivalent).

In context of this study, **2 ml EDTA "full blood"** for HLA-typing and single nucleotide polymorphism (SNP) analysis will be collected once at the beginning of the study or any other time point of the study. Full blood can be stored without further processing at -70℃.

- **All samples** will be store at -70 ℃ until collection via centr al laboratory at

  Labor der Universitäts-Hautklinik Essen

  z.H. Frau Antje Sucker

  Hufelandstr. 55

  45122 Essen

  Antje.sucker@uk-essen.de

6.5.2. Microtissue Array

Formalin Fixed Paraffin Embedded (FFPE) tissue is requested once from every patient. FFPEs blocks from histologically confirmed cutaneous melanoma such as primary tumor or subsequent metastases such as lymph node etc. will be eligable. Collecting samples of fresh tissue is optional. This tissue will be store at -70℃.

DNA will be extracted and tissue micro arrays (TMAs) will be constructed to investigate tumor microenvironment and tumor infiltrating immune cells

6.5.3. Storage and Confidentiality of Specimens

Specimens will be stored in a central laboratory for at least 10 years. Specimens' pseudonymity will be maintained and identities are protected from unauthorized parties. Blood samples and biopsy material are to be labeled with hospital name, type of specimen and patient study number. No personal identifiers will be placed on the tube.

7. INVESTIGATIONAL PRODUCT: IPILIMUMAB

The investigational product is defined as a pharmaceutical form of an active ingredient being tested as a reference in the study. In this study, the investigational product is ipilimumab. Bristol-Myers Squibb (BMS) will provide ipilimumab at no cost for this study.

7.1. Identification

Ipilimumab is available in 5 mg/mL single-use vials (40 mL). The sterile solution in the vial is clear and colorless. Ipilimumab is administered via intravenous infusion only.

7.2. Packaging and Labeling

BMS will provide ipilimumab at no cost for this study. Ipilimumab will be provided in open-label containers. The labels will contain the protocol prefix, batch number, content, storage conditions, and dispensing instructions along with the Investigational New Drug (IND) caution statement. Ipilimumab will be supplied at a concentration of 5 mg/mL in vials containing 40 mL solution.

7.3. Storage, Handling, and Dispensing

7.3.1. Storage

Ipilimumab Injection à 200 mg/vial (5 mg/mL), must be stored in a secure area according to local regulations. The investigator must ensure that it is stored in accordance with the environmental conditions as determined by BMS and defined in the Investigator Brochure or SmPC/reference label. Ipilimumab must be stored at a temperature ≥ 2°C and ≤ 8°C and protected from light In preparation of infusion, ipilimumab may be stored in IV infusion bags (PVC, non-PVC/non-DEHP) or glass infusion containers at room temperature or refrigerated (2ºC - 8ºC) for up to 24 hours. Drug must be completely delivered to the subject within 24 hours of preparation. This includes any time in transit plus the total time for the infusion..

7.3.2. Handling and Disposal

As with all injectable drugs, care should be taken when handling and preparing ipilimumab. Whenever possible, ipilimumab should be prepared in a laminar flow hood or safety cabinet using standard precautions for the safe handling of intravenous agents applying aseptic technique. Latex gloves are required. If ipilimumab concentrate or solution comes in contact with skin or mucosa, immediately and thoroughly wash with soap and water. After final drug reconciliation, unused ipilimumab solution should be disposed at the site following procedures for the disposal of anticancer drugs.

7.3.3. Dispensing

It is the responsibility of the investigator to ensure that ipilimumab is only dispensed to study subjects. The ipilimumab must be dispensed only from official study sites by authorized personnel according to local regulations.

7.4. Drug Ordering and Accountability

7.4.1. Initial Orders

Initial orders of ipilimumab will be requested automatically by Alcedis GmbH following submission and approval of the required regulatory documents.

Adequate supplies of ipilimumab will be provided for each participating center for this study by the cooperating contract research organisation (CRO) Creapharm (Business Development Manager, ZA Air Space Avenue de Magudas CS2007 33187 Le Haillan Cedex France). Study medication supplies must be maintained within a secure location at temperatures between 2°C and 8°C. Drug is protocol specific, but not patient specific.

Product will be shipped by courier in a temperature-controlled container. Shipments will be made on Monday through Thursday for delivery onsite Tuesday through Friday. There will be no weekend or holiday delivery of drugs. It is possible that sites may have more than one ipilimumab clinical study ongoing at the same time. It is imperative that only product designated for this protocol number be used for this study.

7.4.2. Re-Supply

Drug re-supply request form should be submitted to Alcedis GmbH before the expected delivery date. Deliveries will be made Tuesday through Friday. Drug is not patient specific. Be sure to check with your pharmacy regarding existing investigational stock to assure optimal use of drug on hand.

7.5. Ipilimumab Accountability

It is the responsibility of the investigator to ensure that a current record of ipilimumab disposition is maintained at each study site where ipilimumab is inventoried and disposed. Records or logs must comply with applicable regulations and guidelines, and should include:

- Amount received and placed in storage area.
- Amount currently in storage area.
- Label ID number or batch number and use date or expiry date.
- Dates and initials of person responsible for each ipilimumab inventory entry/movement.
- Amount dispensed to and returned by each subject, including unique subject identifiers.
- Amount transferred to another area/site for dispensing or storage.
- Non-study disposition (e.g., lost, wasted, broken).
- Amount destroyed at study site.

7.6. Ipilimumab Destruction

If ipilimumab is to be destroyed on site, it is the investigator's responsibility to ensure that arrangements have been made for disposal and that procedures for proper disposal have been established according to applicable regulations, guidelines, and institutional procedures. Appropriate records of the disposal must be maintained.

8. ADVERSE EVENT REPORTING

8.1. Collection of Safety Information

An ***Adverse Event (AE)*** is defined as any new untoward medical occurrence or worsening of a pre-existing medical condition in a patient or clinical investigation subject administered an investigational (medicinal) product and that does not necessarily have a causal relationship with this treatment. An AE can therefore be any unfavorable and unintended sign (including an abnormal laboratory finding, for example), symptom, or disease temporally associated with the use of investigational product, whether or not considered related to the investigational product.

Adverse events can be spontaneously reported or elicited during open-ended questioning, examination, or evaluation of a subject.

8.1.1. Serious Adverse Events

A ***serious adverse event (SAE)*** is any untoward medical occurrence that at <u>any dose</u>:

- results in death
- is life-threatening (defined as an event in which the subject was at risk of death at the time of the event; it does not refer to an event that hypothetically might have caused death if it were more severe)
- requires inpatient hospitalization or causes prolongation of existing hospitalization (see "note" below for exceptions)
- results in persistent or significant disability/incapacity
- is a congenital anomaly/birth defect
- Suspected transmission of an infectious agent (eg, any organism, virus or infectious particle, pathogenic or non-pathogenic) via the study drug is an SAE.
- is an important medical event (defined as a medical event(s) that may not be immediately life-threatening or result in death or hospitalization but, based upon appropriate medical and scientific judgment, may jeopardize the subject or may require intervention [eg, medical, surgical] to prevent one of the other serious outcomes listed in the definition above.) Examples of such events include, but are not limited to, intensive treatment in an

emergency room or at home for allergic bronchospasm; blood dyscrasias or convulsions that do not result in hospitalization.)

- Although overdose and cancer are not always serious by regulatory definition, these events should be reported on an SAE form and sent to the sponsor and to BMS in an expedited manner. An overdose is defined as the accidental or intentional ingestion or infusion of any dose of a product that is considered both excessive and medically important.

NOTE: The following hospitalizations are not considered SAEs:

- ˜ a visit to the emergency room or other hospital department for less than 24 hours that does not result in admission (unless considered an "important medical event" or a life-threatening event)
- ˜ elective surgery, planned before signing consent
- admissions as per protocol for a planned medical/surgical procedure
- routine health assessment requiring admission for baseline/trending of health status (eg, routine colonoscopy)
- medical/surgical admission for purpose other than remedying ill health state and was planned prior to entry into the study. Appropriate documentation is required in these cases
- admission encountered for another life circumstance that carries no bearing on health status and requires no medical/surgical intervention (eg, lack of housing, economic inadequacy, care-giver respite, family circumstances, administrative).

Note that all pregnancies, regardless of outcome, must be reported to the sponsor. All pregnancies must be reported and followed to outcome, including pregnancies that occur in the female partner of a male study subject. **See Section 8.3.4 for instructions on reporting pregnancies.**

8.1.2. Nonserious Adverse Events

A ***nonserious adverse event*** is an AE not classified as serious.

8.2. Assignment of Adverse Event Intensity and Relationship to Investigational Product

The following categories and definitions of causal relationship to investigational product as determined by a physician should be used:

- **Related:** There is a reasonable causal relationship to investigational product administration and the adverse event.
- **Not Related:** There is not a reasonable causal relationship to investigational product administration and the adverse event.

The expression "reasonable causal relationship" is meant to convey in general that there are facts (eg, evidence such as de-challenge/re-challenge) or other arguments to suggest a positive causal relationship.

8.3. Collection and Reporting

The investigator is responsible for documenting all adverse events that occur during the study. Adverse events can be spontaneously documented or elicited during open-ended questioning, examination, or evaluation of a subject. (In order to prevent reporting bias, subjects should not be questioned regarding the specific occurrence of one or more AEs.)

If known, the diagnosis of the underlying illness or disorder should be recorded, rather than its individual symptoms. The following information should be captured for all AEs: onset, duration, intensity, seriousness, relationship to investigational product, action taken, and treatment required. If treatment for the AE was administered, it should be recorded in the medical record.

The investigator shall supply the sponsor and Ethics Committee with any additional requested information, notably for reported deaths of subjects.

8.3.1. Serious Adverse Events

Following the subject's written consent to participate in the study, all SAEs must be collected, including those thought to be associated with protocol-specified procedures. All SAEs must be collected that occur within 70 days of discontinuation of dosing of the investigational product. If applicable, SAEs must be collected that relate to any later protocol-specified procedure (eg, a follow-up skin biopsy). The investigator should notify the study sponsor of any SAE occurring after this time period that is believed to be certainly, probably, or possibly related to the investigational product or protocol-specified procedure.

All SAEs whether related or unrelated to the ipilimumab, must be immediately reported to the sponsor by using the online documentation system (by the investigator or designee) within 24 hours of becoming aware of the event. If only limited information is initially available, follow-up reports are required.

The sponsor will notify all **serious adverse events to BMS (Pharmacovigilance Germany; Fax:+49(0)89/12142-445).**

For reporting, each serious adverse event must be documented on the electronically available "**Serious adverse events report**" form. The completed form is automatically faxed (fax no. is filed automatically in the system) to the sponsor, to BMS and to Alcedis GmbH. In the event that electronic reporting is not possible, paper SAE forms in the investigator's file handed out at the beginning of the study are at the doctor's disposal for notification by conventional fax. In

some instances where a facsimile machine is not available, overnight express mail may be used.

The initial report to the sponsor, to BMS and to Alcedis GmbH must be followed **within 5 days** by a detailed written communication (in English) containing a precise description of the adverse events, all countermeasures taken and their outcome. In the event of death, if an autopsy has been performed, a copy of the autopsy report should be attached.

The sponsor will notify all **serious adverse events to BMS (germany.arzneimittelsicherheit@bms.com, phone number: +49 89/12142-281 (8-17 Uhr); 24-Stunden, emergency line: +49 89/12142-610; Fax number +49 89/12142-445)**

Serious adverse events, whether related or unrelated to investigational product, must be reported on the SAE form expeditiously to comply with regulatory requirements. An SAE report should be completed for any event where doubt exists regarding its status of seriousness.

If the investigator believes that an SAE is not related to the investigational product, but is potentially related to the conditions of the study (such as withdrawal of previous therapy, or a complication of a study procedure), the relationship should be specified in the narrative section of the SAE form.

If an ongoing SAE changes in its intensity or relationship to the investigational product, a follow-up SAE report should be reported immediately to the sponsor using the same procedure described above. As follow-up information becomes available it should be reported immediately using the same procedure used for reporting the initial SAE report. All SAEs should be followed to resolution or stabilization.

8.3.2. Handling of Expedited Safety Reports

In accordance with local regulations, BMS will notify the sponsor of all SAEs that are suspected (related to the investigational product) and unexpected (ie, not previously described in the Investigator Brochure).Sponsor is obliged to forward ESRs to each investigator. In the European Union (EU), an event meeting these criteria is termed a Suspected, Unexpected Serious Adverse Reaction (SUSAR). Investigator notification of these events will be in the form of an expedited safety report (ESR).

Other important findings which may be reported by BMS to the sponsor as an ESR include: increased frequency of a clinically significant expected SAE, an SAE considered associated with study procedures that could modify the conduct of the study, lack of efficacy that poses significant hazard to study subjects, clinically significant safety finding from a nonclinical (eg,

animal) study, important safety recommendations from a study data monitoring committee, or sponsor decision to end or temporarily halt a clinical study for safety reasons.

Upon receiving an ESR from sponsor, the investigator must review and retain the ESR with the Investigator Brochure. Where required by local regulations or when there is a central IRB/IEC for the study, the sponsor will submit the ESR to the appropriate IRB/IEC. The sponsor and IRB/IEC will determine if the informed consent requires revision. The investigator should also comply with the IRB/IEC procedures for reporting any other safety information. Where required, submission of ESRs by the investigator to Health Authorities should be handled according to local regulations.

In addition, suspected unexpected serious adverse reactions shall be reported to the relevant competent health authorities in all concerned countries according to local regulations (either as expedited and/or in aggregate reports).

8.3.3. Non-serious Adverse Events

The collection of non-serious AE information should begin from time of consent forward until end of treatment.

If an ongoing nonserious AE worsens in its intensity, or if its relationship to the investigational product changes, a new nonserious AE entry for the event should be completed. Nonserious AEs should be followed to resolution or stabilization, or reported as SAEs if they become serious. Follow-up is also required for nonserious AEs that cause interruption or discontinuation of investigational product, or those that are present at the end of study participation. Subjects with nonserious AEs at study completion should receive post-treatment follow-up as appropriate.

All identified nonserious AEs must be documented and described in the medical record.

8.3.4. Pregnancy

Sexually active WOCBP must use an effective method of birth control during the course of the study, in a manner such that risk of failure is minimized. Before enrolling WOCBP in this clinical study, the investigator must review the guideline about study participation for WOCBP which can be found in the GCP Manual for Investigators. The topics include the following:

- General Information
- Informed Consent Form
- Pregnancy Prevention Information Sheet
- Drug Interactions with Hormonal Contraceptives
- Contraceptives in Current Use

- Guidelines for the Follow-up of a Reported Pregnancy.

Before study enrollment, WOCBP must be advised of the importance of avoiding pregnancy during study participation and the potential risk factors for an unintentional pregnancy. The subject must sign an informed consent form documenting this discussion.

All WOCBP MUST have a negative pregnancy test within 72 hours before receiving ipilimumab. The minimum sensitivity of the pregnancy test must be 25 IU/L or equivalent units of HCG. If the pregnancy test is positive, the subject must not receive ipilimumab and must not be enrolled in the study.

In addition, all WOCBP should be instructed to contact the investigator immediately if they suspect they might be pregnant (e.g., missed or late menstrual period) at any time during study participation.

If, following initiation of the investigational product, it is subsequently discovered that a study subject is pregnant or may have been pregnant at the time of investigational product exposure, including during at least 6 half-lives after product administration, the investigational product will be permanently discontinued in an appropriate manner (eg, dose tapering if necessary for subject safety). Pregnancy occurring during the clinical investigation, although not considered a serious adverse event, must be reported to the sponsor, BMS and Alcedis GmbH within the same timelines as a serious adverse event and using the same procedure as described for the reporting of SAEs. The outcome of a pregnancy should be followed up carefully and any abnormal outcome of the mother or the child should be reported.

Any pregnancy that occurs in a female partner of a male study participant should be reported to the sponsor. Information on this pregnancy will also be reported as described for SAE-reporting.

Protocol-required procedures for study discontinuation and follow-up must be performed on the subject unless contraindicated by pregnancy (e.g., x-ray studies). Other appropriate pregnancy follow-up procedures should be considered if indicated. In addition, the investigator must report to the sponsor and BMS, and follow-up on information regarding the course of the pregnancy, including perinatal and neonatal outcome. Infants should be followed for a minimum of 8 weeks.

8.3.5. Other Safety Considerations

Any significant worsening noted during interim or final physical examinations, electrocardiograms, x-rays, and any other potential safety assessments, whether or not these procedures are required by the protocol, should also be recorded in the medical record.

9. STATISTICAL METHODOLOGY

In this study, up to 200 patients with unresectable stage III-IV melanoma will be treated with the anti-CTLA-4 mAb Ipilimumab monotherapy over a recruitment period of 8-9 months at up to 25 centers in Germany.

9.1. Sample Size Justification

Assuming that the entire study cohort will behave as cutaneous melanoma treated with 10mg/kg including maintenance therapy, the 1-year-survival rate will be comparable to the 1-year-survival rate seen in BMS CA184-007,-008, and -022 studies, i.e about 50% (0.5), and the width of the 95% CI will maximally be 14%, corresponding to a lower confidence limit of 0.43 and an upper of 0.57. The 1-year-survival rate in the MDX010-020 study published in NEJM (Hodie et al., 2010) is 43% and the 2-year-survival rate 23% for both ipilimumab arms combined using the 3 mg/kg dosing scheme 4-times over 12 weeks.

The proposed study includes patient subgroups (uveal, mucosal) which we currently have only limited efficacy data for ipilimumab on. Retrospective studies suggest the median survival for mucosal melanoma in Stage IV is around 6 months corresponding - assuming an exponential distribution - to about 13% survival rate at 1 year, and median survival for uveal melanoma is around 11 months corresponding to about 25% survival rate at 1 year (Becker et al., 2002; Penel et al., 2008).

Given the differing median survivals for uveal and mucosal melanoma prior to treatment with ipilimumab, the OS at 1 year will be presented separately for the cohorts, uveal, mucosal and cutaneous melanoma. Assuming 10% (N=20) subjects enrolled have uveal melanoma, and a further 10% have mucosal melanoma, whilst 80% (N=160) of those enrolled have cutaneous melanoma, the width of the 95%CI will, for any survival rate at 1 year, maximally span 41% for the uveal and mucosal cohorts, and 18% for the cutaneous cohort.

9.2. Data collection

The data collected in this study will be analyzed by the Sponsor or its designees. All data will be listed individually by patient. For quantitative parameters, descriptive statistics will include the mean, standard deviation, minimum, median, and maximum. For qualitative parameters, descriptive statistics will include the frequency and percentage.

9.3. Endpoints

9.3.1. Primary Efficacy Parameter

The primary endpoint is the one-year survival rate. It is defined as the proportion of patients being alive 12 months after their first administration of the study treatment (ipilimumab).

9.3.2. Secondary Efficacy Parameters

To assess safety and additional efficacy parameters of the ipilimumab treatment.

Secondary endpoints are

- Safety / toxicity according to the CTC Criteria (Version 4.0): All adverse events ≥ Grade 3 according to CTCAE, Version 4.0 criteria, that are definitely, probably, or possibly related to the administration of the investigational agents will be assessed and described using descriptive statistics.
- Overall response rate - irOR (irBOR of irPR or irCR) according to immune-related response Criteria (ir-RC) (see 6.3.4.5), and OR (BOR of PR or CR) according to RECIST (see 6.3.3.3).
- Disease control rate - irDCR (irBOR of irCR or irPR or irSD) according to immune-related response Criteria (ir-RC) (see section 6.3.4.5), and DCR (BOR of PR or CR) according to RECIST (see 6.3.3.3).
- Progression free survival rate at 6 months. PFS is defined as the time from the first study treatment date until documented tumor progression (irPD) date (as defined by immune related response criteria) or date of death, whichever occurs first.

Exploratory endpoints

Pharmacodynamic effects of ipilimumab on Absolute Lymphocyte Count (ALC) and correlation of ALC to anti-tumor activity (ORR) of ipilimumab during induction phase (W12)

9.4. Study population

Intention to Treat (ITT) = Safety population: All patients who were enrolled and received at least one dose of ipilimumab.

Response-evaluable population: All subjects who were treated with at least one dose of ipilimumab with (i) measurable disease at baseline as determined by the irRC; (ii) histologic diagnosis of malignant melanoma; and (iii) at least one screening (i.e. baseline) and at least one on-study TA.

9.5. Statistical analysis

The primary endpoint, one year survival rate, is based on the ITT population. As a simple rate, it will be calculated as the number of patients being still alive 12 months after their first administration of the study treatment divided by the number of patients having received at least one administration of the study treatment. Only subjects known to be alive at 1 year will be included in the numerator. As a time to event variable, it will be,derived from the Kaplan-Meier curve. In this case missing data will be censored at the last contact. See below for further details.

All other analyses will have exploratory character. Results will be presented overall and per cohort/subgroup:

- (1) cutaneous vs uveal vs. mucosal melanoma
- (2) - LDH < 2 UNL versus LDH ≥ 2 UNL
- (3) - status brain mets versus no brain mets in medical history
- (4) - status Braf mutation positive versus. Braf mutation negative
- (5) - HLA-A2 positive versus HLA-A2 negative

Demographic and baseline characteristics will be tabulated for all treated subjects. For categorical variables, summary tabulations of the number and percentage within each category (with a category for missing data) of the parameter will be presented. For continuous variables, the mean, median, standard deviation, minimum and maximum values will be given.

Proportions (Survival rate at 1 year, irOR, irDCR, irPFS at 6 mths, OR and DCR) will be presented with exact (Clopper and Pearson) two-sided 95% CI. irOR, irDCR and irPFS are defined according to the irRC; OR and DCR acording to RECIST; The date of origin for the survival rate at one year, and irPFS at 6 months is the date of the first ipilimumab dose. Only subjects known to be progression free at 6 months / alive at 1 year will be included in the respective numerators. Any subject receiving at least one dose of ipilimumab will be included in the denominators. The denominator for the irOS and irPFS analyses will be the ITT population. The denominator for the irOR, and irDCR, OR and DCR will be the Response-evaluable population..

Additional analyses treating survival and PFS as time to event endpoints will be performed i.e.: Kaplan-Meier plots will be produced. These will use the ITT population. Survival will be measured, regardless of any re-induction, from the date of the first on-study ipilimumab dose until date of death. Survival time for subjects, whose date of death is unknown, will be censored at the last date of contact. PFS will be measured, regardless of any re-induction, from the date of the first dose of ipilimumab until the first on-study documented date of progression, as per irRC, or date of death, whichever comes first. PFS time of subjects not known to progress or die, will be censored at the date of the last tumor assessment. Median survival, median PFS, survival rate at 1 year and PFS rate at 6 months, all as derived from the Kaplan-Meier curves, will be presented, together with 95%CI.

Safety will be reported for 70 days (5 half-lives) post last treatment. This period will be considered the on-study safety period. The analysis of safety will be based on the frequency of AEs and their severity for all treated subjects (i.e.: the Safety population). Worst toxicity grades per subject will be tabulated for all AEs. Additionally, drug-related grade 3 or more AEs, immune-related, and serious AEs will be tabulated separately, and listings by subject will be produced for all deaths, all SAEs and all AEs leading to discontinuation of study drug.

All adverse events and serious adverse events recorded during the study will be summarized using descriptive statistics. The incidence of treatment-emergent adverse events (new or worsening from baseline) will be summarized by body system, preferred term, verbatim of adverse event, intensity (based on CTC grades), and relation to the investigational products. Furthermore all adverse events and serious adverse events will be listed.

9.6. Statistical Software

All statistical analyses will be performed using SAS version 9.2 or higher.

10. DATA MANAGEMENT

10.1. Patient Identification

All data related to patients will be assessed anonymously. Each patient will be clearly identified through the patient number given in the enrolment procedure. At the centre site the investigator compiles a confidential list, in which the patient name and address is assigned to the patient number.

10.2. Data Collection

The data management for this study will be performed by Alcedis GmbH, Winchesterstr. 2, D-35394 Gießen.

The following chapters describe the software employed and measures applied for data security.

Data are recorded, processed and stored using the following software tools.

a. CRF database (Location: Alcedis)

b. SAE database (Location: Alcedis)

Wherever applicable, current GCP guidelines, actual technical standards and guidelines are observed.

10.2.1. Employed Software

CRF database

For data capturing and data management of this clinical trial, a web-based validated software (WBDC) will be employed. The software consists of the following modules:

a) **Administration:** Administration of sites (clinics / office based physicians) by system administrator and project management. Within the individual sites the following system users are defined: Investigator and study nurse. All access rights are administered in a role-based security system.

b) **Forms / Form validator:** Electronic Case Report Forms (eCRFs) for data capture including online validation of CRFs during data capture, e. g. check on range, plausibility, type mismatch.
In addition to the system based plausibility checks, a formal query process will be implemented to resolve inconsistencies in SAE data.

c) **Reports:** Dynamic report generator, e.g. reports for investigators on CRF status.

d) **Database:** Relational database for data management. The data from the relational database will be retrieved using the export engine of *Alcedis Med* and thereafter converted into data sets for further validation and analysis.

The employed technology and technical requirements for data entry on site are as follows:

a) The used software is completely server-based, i.e. all programme processes are executed centrally on a web or database server.
b) Data are saved exclusively in the central database server. This server is located in the facilities of Alcedis GmbH, Winchesterstr. 2, D-35394 Giessen.
c) For system access, users require a conventional desktop computer with internet access.

10.2.2. Data Security and Storage

For client / server communication via the Internet only encrypted transmissions are applied. State of the art encryption technology is used exclusively. For data transmission in this clinical trial an encryption level (128-bit) is employed by means of the Secure Socket Layer Algorithm (SSL).

In addition, the server identifies itself to the client workstation by means of a digital server certificate issued by an authorised certification authority. This ensures that data are sent only to the server of Alcedis GmbH.

Data are protected from potential virtual attacks and physical damage.

Views on data or reports as well as edit or read only rights are controlled with individual passwords. Access authorisation to the CRF databases is granted individually to investigators and programme personnel by means of user accounts.

The project management of the CRO has a read-only access on all patient data stored in the CRF database.

Assurance of data will be made by RAID-Systems (Redundant Array of Independent Disks), thereby ensuring data security even if one hard disc failed.

Furthermore a back up onto magnetic tape is performed according to the following scheme:

- daily back-up over a period of 7 days
- weekly back-up over a period of 5 weeks
- monthly back-up over a period of continuance of the clinical trial

Investigators will get a CD-Rom after the end of the trial containing the data of the patients they have documented.

11. MONITORING

11.1. Monitoring

Study monitoring is undertaken by monitors appointed by ALCEDIS GMBH. The responsible monitor will be allowed, on request, to inspect the various records of the trial (Case Report Forms and other pertinent data).

Due to the electronic documentation system checks for range and plausibility are performed during data entry. The monitor gets an access to read the data only.

In line with ICH GCP guidelines, monitoring will include verification of data entered in the eCRF against original patient records. This verification will be performed by direct access to the original subject records, and the Sponsor guarantees that patient confidentiality will be respected at all times. Participation in this study will be taken as agreement to permit direct source data verification.

11.2. Audit, Inspections

Authorized representatives of the sponsor must be allowed to visit all study site locations periodically to assess the data quality and study integrity. On site they will review study records and directly compare them with source documents, discuss the conduct of the study with the investigator, and verify that the facilities remain acceptable. In addition, the study may be evaluated by sponsor´s internal auditors and government inspectors who must be allowed access to CRFs, source documents, other study files, and study facilities. Audit reports will be kept confidential. THE INVESTIGATOR MUST NOTIFY SPONSOR PROMPTLY OF ANY INSPECTIONS SCHEDULED BY REGULATORY AUTHORITIES, AND PROMPTLY FORWARD COPIES OF INSPECTION REPORTS TO SPONSOR.

12. Legal and ADMINISTRATIVE SECTION

12.1. Coordinating Investigator in Accordance with Section 40 AMG

Prof. Dr. Dirk Schadendorf
Department of Dermatology, Skin Cancer Center
Comprehensive Cancer Center Essen
Hufelandstr. 55
D- 45122 Essen,
Germany
Telephone Number: +49 (0) 201-723 4342
Fax Number: +49 (0) 201-723 5935
e-mail: dirk.schadendorf@uk-essen.de

The principal investigator can demonstrate at least 2 years' experience in the clinical testing of medicinal products.

12.2. Compliance with the Protocol and Protocol Revisions

The study shall be conducted as described in this approved protocol. All revisions to the protocol must be discussed with Coordinating Invesigator, and be prepared by designated person. The investigator should not implement any deviation or change to the protocol without prior review and documented approval/ favorable opinion from the IRB/IEC of an amendment, except where necessary to eliminate an immediate hazard(s) to study subjects. Any significant deviation must be documented in the eCRF. If a deviation or change to a protocol is implemented to eliminate an immediate hazard(s) prior to obtaining IRB/IEC approval/favorable opinion, as soon as possible the deviation or change will be submitted to:

• IRB/IEC for review and approval/favorable opinion
• Regulatory Authority(ies), if required by local regulations

Moreover, Bristol-Myers Squibb will be informed.

Documentation of approval signed by the chairperson or designee of the IRB(s)/IEC(s) must be sent to BMS. If an amendment substantially alters the study design or increases the potential risk to the subject: (1) the consent form must be revised and submitted to the IRB(s)/IEC(s) for review and approval/favorable opinion; (2) the revised form must be used to obtain consent from subjects currently enrolled in the study if they are affected by the amendment; and (3) the new form must be used to obtain consent from new subjects prior to

enrollment. If the revision is an administrative letter, investigators must inform their IRB(s)/IEC(s).

12.3. Good Clinical Practice

This study will be conducted in accordance with Good Clinical Practice (GCP), as defined by the International Conference on Harmonisation (ICH) and in accordance with the ethical principles underlying European Union Directive 2001/20/EC and the United States Code of Federal Regulations, Title 21, Part 50 (21CFR50).

The study will be conducted in compliance with the protocol. The protocol and any amendments and the subject informed consent will receive Institutional Review Board/Independent Ethics Committee (IRB/IEC) approval/favorable opinion prior to initiation of the study.

All potential serious breaches must be reported to the Sponsor immediately. A serious breach is a breach of the conditions and principles of GCP in connection with the study or the protocol, which is likely to affect, to a significant degree, the safety or physical or mental integrity of the subjects of the study or the scientific value of the study.

Study personnel involved in conducting this study will be qualified by education, training, and experience to perform their respective task(s). This study will not use the services of study personnel where sanctions have been invoked or where there has been scientific misconduct or fraud (eg, loss of medical licensure, debarment).

Systems with procedures that ensure the quality of every aspect of the study will be implemented.

12.4. Study Monitoring

The sponsor and the investigators must ensure the clinical trial is conducted correctly.

12.5. Maintenance of a Study File

The principal investigator will keep all study-relevant documents (e.g. study plan, curriculum vitae, ethics committee approval) and correspondence in a special study file.

12.6. Authorisation of Competent Authority

The documents on the pharmacological and toxicological testing of ipilimumab are filed with the competent authority in accordance with section 40 AMG. The application for approval of a

clinical trial with a medicinal product for human use is made on behalf of the sponsor by Acedis GmbH.

12.7. Ethics Committee Approval

Before study initiation, the sponsor must have written and dated approval/favorable opinion from the IRB/IEC for the protocol, consent form, subject recruitment materials/process (eg, advertisements), and any other written information to be provided to subjects. The investigator should also provide the IRB/IEC with a copy of the Investigator Brochure or product labeling, information to be provided to subjects and any updates.

The sponsor should provide the IRB/IEC with reports, updates and other information (eg, expedited safety reports, amendments, and administrative letters) according to regulatory requirements or institution procedures.

12.8. Notification to the Competent Supervisory Authorities

Participating investigators are notified to the competent supervisory authorities by Alcedis GmbH.

12.9. Archival

The investigator must retain investigational product disposition records, copies of CRFs (or electronic files), and source documents for the maximum period required by applicable regulations and guidelines, or institution procedures, or for the period specified by the sponsor, whichever is longer. If the investigator withdraws from the study (eg, relocation, retirement), the records shall be transferred to a mutually agreed upon designee (eg, another investigator, IRB). Notice of such transfer will be given in writing to BMS

The investigator is legally obliged to keep the patient identification list for at least 10 years after the end of the study. The patient data recorded, including the original or copies of test results, the informed consents, the ethics committee approval and the correspondence and other original documents associated with the study must also be stored by the investigator for a period of 10 years. This precondition also applies if the doctor transfers the documents (and the associated obligation of storage) to a successor.

Original data from the study patients (patient records) must be stored in accordance with the archiving period applicable in the study centres, but for not less than 10 years.

12.10. Confidentiality

The contents of the study protocol and case report form must be treated confidentially and may not be disclosed to unauthorised parties either verbally or in writing.

12.11. Patient Insurance

The study patients are insured in accordance with section 40 AMG up to a sum of €500,000.00.

Policy Number 57010328 03018

12.12. Final Report

Once the final biometric report is available, the final medical report will be compiled by the study director

12.13. Third Party Financing

This study will entail no additional financial expenditure either for the organisations funding the hospital or for the health insurance companies in association with the clinical trial. Third party funds will be provided by Bristol-Myers Squibb Company for study co-ordination, documentation, monitoring and analysis.

This study entails no additional financial expenditure for supplementary laboratory analyses or additional diagnostic measures associated with the study therapy, as the study design is deliberately based on the procedure for the cytostatic therapy administered in the previous standard.

13. ETHICAL CONSIDARATIONS

13.1. Declaration of Helsinki

The study is conducted in accordance with the 1996 Declaration of Helsinki (Somerset West, Republic of South Africa, Appendix 7).

13.2. Patient Information

An unconditional prerequisite for a patient participating in the study is his/her written informed consent. Adequate information must therefore be given to the subject by the investigator before informed consent is obtained. A patient information sheet in the local language will be provided for the purpose of obtaining informed consent. In addition to this written information, the investigator will inform the patient verbally. In doing so, the wording used will be chosen so that the information can be fully and readily understood by laypersons.
The patient information sheet will be revised whenever important new information becomes available that may be relevant to the consent of patients.

The written informed consent of the patient to participate in the clinical study has to be given before any study-related activities are carried out. It must be signed and personally dated by the patient and by the investigator/person designated by the investigator to conduct the informed consent discussion.

Provision of consent will be confirmed in the CRF by the investigator. The signed and dated declaration of informed consent will remain at the investigator's site and must be safely archived by the investigator so that the forms can be retrieved at any time for monitoring, auditing and inspection purposes. A copy of the signed and dated information and consent should be provided to the subject prior to participation.

A template for the subject information sheet and corresponding subject informed consent form expression is provided in Appendix 9.

13.3. Informed Consent

All subjects will be at least 18 years old. Before being admitted to the clinical trial, the subject will consent to participate after the nature, scope, and possible consequences of the clinical trial have been explained in a form understandable to him or her.. Freely given written informed consent must be obtained from every subject prior to clinical study participation, including informed consent for any screening procedures conducted to establish subject eligibility for the study. The rights, safety, and well-being of the study subjects are the most important considerations and should prevail over interests of science and society. The informed consent form can be found in Appendix 9.

13.4. Use, Storage and Transmission of Data

Patients will be informed that data, related to their illness, will be saved and in anonymous form used for scientific analysis and publications. Patients are entitled to get information about the saved data. The informed consent to data security and data transfer will be given apart of the informed consent to study participation (Appendix 9).

Appendix 1: List of Abbreviations

Abbreviation	Term
ABE	Automatical Breakthrough Event
AE	Adverse Event
ALC	Absolute Lymphocyte Count
ALT	Alanine Aminotransferase (SGPT)
ANC	Absolute Neutrophil Count
AST	Aspartate aminotransferase (SGOT)
BID	Twice a Day
BMS	Bristol-Myers Squibb Company
BORR	Best Overall Response Rate
CBC	Complete Blood Count
CR	Complete Response
CT scan	Computed Axial Tomography scan
CTC AE	Common Terminology Criteria for Adverse Events
DCR	Disease Control Rate
DLT	Dose Limiting Toxicity
DSMB	Data Safety Monitoring Board
ECG	Electrocardiogram
ECOG PS	Eastern Cooperative Oncology Group Performance Status
eCRF	electronic Case Report Form
ESR	Expedited safety report
HIPAA	Health Insurance Portability and Accountability Act
IC	Informed Consent
irAE	immune-related adverse event (irAE)
IRB	Institutional Review Board
irBOR	Immune-Related Best Overall Response
irRC	Immune Related Response Criteria
ir – PD	Immune-Related Progressive Disease
ir – RC	Immune-Related Response Criteria
LDH	Lactat-Dehydrogenase
LFT	Liver Function Tests

LPFV	Last Patient First Visit
MRI	Magnetic Resonance Imaging
NCI	National Cancer Institute
OR	Overall Response
ORR	Overall Response Rate
PD	Progressive Disease
PFS	Progression Free Survival
PI	Principal Investigator
PO	By Mouth
PR	Partial Response
QD	Once Daily
QoL	Quality Of Life
RECIST	Response Evaluation Criteria In Solid Tumors
SAE	Serious Adverse Event
SD	Stable Disease
SGOT	Serum Glutamic Oxaloacetic Transaminase (AST)
SGPT	Serum Glutamic Pyruvate Transaminase (ALT)
SPD	Sum of the Products Diameters
SPN	Single Nucleotide Polymorphism
TA	Tumor Assessment
T. Bilirubin	Total Bilirubin
TNM Staging	Tumor, Node and Metastasis Staging
TSH	Thyroid Stimulating Hormone
ULN	Upper Limit of Normal
WBC	White Blood Cells
WHO	World Health Organization
WOCBP	Women of Childbearing Potential

Appendix 2: AJCC 2010- Melanoma TNM Classification

(Balch et al., JCO 2009)

T classification	Thickness	Ulceration Status
**T1**	≤1,0 mm	a: without ulceration and level II/III<br>b: with ulceration or level III/IV
**T2**	1.01-2.0 mm	a: without ulceration<br>b: with ulceration
**T3**	2.01-4 mm	a: without ulceration<br>b: with ulceration
**T4**	>4 mm	a: without ulceration<br>b: with ulceration

N classification	No. of Metastatic Nodes	Nodal Metastatic Mass
**N1**	1 node	a: micrometastasis[1]<br>b: macrometastasis[2]
**N2**	2-3 nodes	a: micrometastasis<br>b: macrometastasis,<br>c: in transit met(s)/satellite(s) without metastatic nodes
**N3**	4 or more metastatic nodes,<br>or matted nodes,<br>or in transit met(s)/satellite(s) with metastatic nodes	

M classification	Site	Serum Lactate Dehydrogenase
**M1a**	Distant skin, sc. Or nodal mets	Normal
**M1b**	Lung metastases	Normal
**M1c**	All other visceral metastases	Normal
	Any distant metastasis	Elevated

[1]: micrometastases are diagnosed after sentinel or elective lymphadenectomy; [2]: macrometastases are defined as clinically detectable nodal metastases confirmed by therapeutic lymphadenectomy or when nodal metastasis exhibits gross extracapsular extension

AJCC 2010- Proposed Stage Groupings for Cutaneous Melanoma

(Balch et al., JCO 2009)

	**Clinical Staging**[1]			**Pathological Staging**[2]		
	T	N	M	T	N	M
0	Tis	N0	M0	Tis	N0	M0
IA	T1a	N0	M0	T1a	N0	M0
IB	T1b	N0	M0	T1b	N0	M0
	T2a	N0	M0	T2a	N0	M0
IIA	T2b	N0	M0	T2b	N0	M0
	T3a	N0	M0	T3a	N0	M0
IIB	T3b	N0	M0	T3b	N0	M0
	T4a	N0	M0	T4a	N0	M0
IIC	T4b	N0	M0	T4b	N0	M0
III[3]	Any T	N1	M0			
		N2				
		N3				
IIIA				T1-4a	N1a	M0
				T1-4a	N2a	M0
IIIB				T1-4b	N1a	M0
				T1-4b	N2a	M0
				T1-4a	N1b	M0
				T1-4a	N2b	M0
				T1-4a/b	N2c	M0
IIIC				T1-4b	N1b	M0
				T1-4b	N2b	M0
				Any T	N3	M0
IV	Any T	Any N	Any M1	Any T	Any N	Any M1

[1]: Clinical staging includes microstaging of the primary melanoma and clinical /radiological evaluation for metastases. By convention, it should be used after complete excision of the primary melanoma with clinical assessment for regional and distant metastases.

[2]: Pathologic staging includes staging of the primary melanoma and pathologic information about the regional lymph nodes after partial or complete lymphadenectomy. Pathologic stage 0 or stage 1A patients are the exception; they do not require pathologic evaluation of their lymph nodes.

[3]: There are no stage III subgroups for clinical staging

Appendix 3: Participating Trial Centers

(Proposal from June 2010)

Trial Center	Investigator
Essen	Prof. Dr. Schadendorf, OA Dr. L. Zimmer
Buxtehude	OA Dr. Mohr
Frankfurt/Main	Prof. Dr. Kaufmann,
Homburg/Saar	Prof. Dr. Vogt, OA PD Dr. Rass
Kiel	Prof. Dr. Hauschild
München/LMU	Prof. Dr. C. Berking
Tübingen	Prof. Dr. Garbe
Göttingen	Prof. Dr. Thoms
Köln	Prof. Dr. C. Mauch
Ludwigshafen	Prof. Dr. E. Dippel
Mainz	Prof. Dr. Grabbe, OA Dr. C. Loquai
Heidelberg	Prof. Dr. Enk/ OA Dr. J. Hassel
Münster	Prof. Dr. Sunderkötter
Hornheide	Prof. Dr. Atzpodien
Regensburg	Prof. Dr. Landthaler
Erfurt	Prof. Dr. Herbst
Jena	Prof. Dr. Norgauer
Leipzig	Prof. Dr. Simon
Minden	Prof. Dr. Stadler
Nürnberg	Prof. Dr. Schultz
Kassel	Prof. Dr. Rompel
Erlangen	Prof. Dr. Kämpgen
Mannheim	PD Dr. Utikal
Lübeck	PD Dr. Terheyden
Quedlinburg	PD Ulrich
RESERVE<br>Freiburg, Dresden, Ulm, Augsburg, Würzburg, Schwerin	

Appendix 4: ECOG Performance Status

Grade	Description
**0**	Fully active, able to carry on all pre-disease performance without restriction.
**1**	Restricted in physically strenuous activity but ambulatory and able to carry out work of a light or sedentary nature (e.g. light housework, office work).
**2**	Ambulatory and capable of all self-care but unable to carry out any work activities. Up and about more than 50% of waking hours.
**3**	Capable of only limited self-care, confined to bed or chair more than 50% of waking hours.
**4**	Completely disabled. Cannot carry on any self-care. Totally confined to bed or chair.
**5**	Death

Appendix 5: Declaration of Helsinki

WORLD MEDICAL ASSOCIATION DECLARATION OF HELSINKI

World Medical Association
DECLARATION OF HELSINKI

Recommendations Guiding Physicians
in Biomedical Research Involving Human Subjects
Adopted by the 18th World Medical Assembly
Helsinki, Finland, June 1964
and amended by the
29th World Medical Assembly
Tokyo, Japan, October 1975
35th World Medical Assembly
Venice, Italy, October 1983
41st World Medical Assembly
Hong Kong, September 1989
and the
48th General Assembly
Somerset West, Republic of South Africa, October 1996.

INTRODUCTION

It is the mission of the physician to safeguard the health of the people. His or her knowledge and conscience are dedicated to the fulfilment of this mission.

The Declaration of Geneva of the World Medical Association binds the physician with the words, "The health of my patient will be my first consideration," and the International Code of Medical Ethics declares that, "A physician shall act only in the patient's interest when providing medical care which might have the effect of weakening the physical and mental condition of the patient."

The purpose of biomedical research involving human subjects must be to improve diagnostic, therapeutic and prophylactic procedures and the understanding of the etiology and pathogenesis of disease.

In current medical practice most diagnostic, therapeutic or prophylactic procedures involve hazards. This applies especially to biomedical research.

Medical progress is based on research which ultimately must rest in part on experimentation involving human subjects.

In the field of biomedical research a fundamental distinction must be recognised between medical research in which the aim is essentially diagnostic or therapeutic for a patient, and medical research, the essential object of which is purely scientific and without implying direct diagnostic or therapeutic value to the person subjected to the research.

Special caution must be exercised in the conduct of research which may affect the

environment, and the welfare of animals used for research must be respected.

Because it is essential that the results of laboratory experiments be applied to human beings to further scientific knowledge and to help suffering humanity, the World Medical Association has prepared the following recommendations as a guide to every physician in biomedical research involving human subjects. They should be kept under review in the future. It must be stressed that the standards as drafted are only a guide to physicians all over the world. Physicians are not relieved from criminal, civil and ethical responsibilities under the laws of their own countries.

I. BASIC PRINCIPLES

1. Biomedical research involving human subjects must conform to generally accepted scientific principles and should be based on adequately performed laboratory and animal experimentation and on a thorough knowledge of the scientific literature.
2. The design and performance of each experimental procedure involving human subjects should be clearly formulated in an experimental protocol which should be transmitted for consideration, comment and guidance to a specially appointed committee independent of the investigator and the sponsor provided that this independent committee is in conformity with the laws and regulations of the country in which the research experiment is performed.
3. Biomedical research involving human subjects should be conducted only by scientifically qualified persons and under the supervision of a clinically competent medical person. The responsibility for the human subject must always rest with a medically qualified person and never rest on the subject of the research, even though the subject has given his or her consent.
4. Biomedical research involving human subjects cannot legitimately be carried out unless the importance of the objective is in proportion to the inherent risk to the subject.
5. Every biomedical research project involving human subjects should be preceded by careful assessment of predictable risks in comparison with foreseeable benefits to the subject or to others. Concern for the interests of the subject must always prevail over the interest of science and society.
6. The right of the research subject to safeguard his or her integrity must always be respected. Every precaution should be taken to respect the privacy of the subject and to minimize the impact of the study on the subject's physical and mental integrity and on the personality of the subject.
7. Physicians should abstain from engaging in research projects involving human subjects unless they are satisfied that the hazards involved are believed to be predictable. Physicians should cease any investigation if the hazards are found to outweigh the potential benefits.
8. In publication of the results of his or her research, the physician is obliged to preserve the accuracy of the results. Reports of experimentation not in accordance with the principles laid down in this Declaration should not be accepted for publication.

9. In any research on human beings, each potential subject must be adequately informed of the aims, methods, anticipated benefits and potential hazards of the study and the discomfort it may entail. He or she should be informed that he or she is at liberty to abstain from participation in the study and that he or she is free to withdraw his or her consent to participation at any time. The physician should then obtain the subject's freely-given informed consent, preferably in writing.

10. When obtaining informed consent for the research project the physician should be particularly cautious if the subject is in a dependent relationship to him or her or may consent under duress. In that case the informed consent should be obtained by a physician who is not engaged in the investigation and who is completely independent of this official relationship.

11. In case of legal incompetence, informed consent should be obtained from the legal guardian in accordance with national legislation. Where physical or mental incapacity make it impossible to obtain informed consent, or when the subject is a minor, permission from the responsible relative replace that of the subject in accordance with national legislation.

    Whenever the minor child is in fact able to give a consent, the minor's consent must be obtained in addition to the consent of the minor's legal guardian.

12. The research protocol should always contain a statement of the ethical considerations involved and should indicate that the principles enunciated in the present Declaration are complied with.

II. MEDICAL RESEARCH COMBINED WITH PROFESSIONAL CARE (Clinical Research)

1. In the treatment of the sick person, the physician must be free to use a new diagnostic and therapeutic measure, if in his or her judgement it offers hope of saving life, reestablishing health or alleviating suffering.

2. The potential benefits, hazards and discomfort of a new method should be weighed against the advantages of the best current diagnostic and therapeutic methods.

3. In any medical study, every patient - including those of a control group, if any - should be assured of the best proven diagnostic and therapeutic method. This does not exclude the use of inert placebo in studies where no proven diagnostic or therapeutic method exists.

4. The refusal of the patient to participate in a study must never interfere with the physician-patient relationship.

5. If the physician considers it essential not to obtain informed consent, the specific reasons for this proposal should be stated in the experimental protocol for transmission the independent committee (see I,2).

6. The physician can combine medical research with professional care, the objective being the acquisition of new medical knowledge, only to the extent that medical research is justified by its potential diagnostic or therapeutic value for the patient.

III. NON-THERAPEUTIC BIOMEDICAL RESEARCH INVOLVING HUMAN SUBJECTS (Non-Clinical Biomedical Research)

1. In the purely scientific application of medical research carried out on a human being, it is the duty of the physician to remain the protector of the life and health of that person on whom biomedical research is being carried out.

2. The subjects should be volunteers -- either healthy persons or patients for whom the experimental design is not related to the patient's illness.
3. The investigator of the investigation team should discontinue the research if in his/her or their judgement it may, if continued, be harmful to the individual.
4. In research on man, the interest of science and society should never take precedence over considerations related to the well-being of the subject.

Appendix 6: Common Toxicity Criteria (CTCAE) Scale

For an updated and detailed list of common toxicities please refer to the online version of the Common Toxicity Criteria version 4.0 on http://www.acrin.org/Portals/0/Administration/Regulatory/CTCAE_4.02_2009-09-15_QuickReference_5x7.pdf.

If uncertain or if your toxicity cannot be graded using the CTCAEv4, please contact the coordinating investigator.

Appendix 7: Patient Information and Informed Consent

Appendix 8: irAE Treatment Algorithm

Re-Induction:

Patients who progress following stable disease of ≥ 3 months starting from diagnosis at week 12 tumor assessment duration from beginning of induction treatment or or patients who have progressed following an initial response (partial or complete) assessed at week 12 may be offered additional cycles of therapy with the originally assigned treatment regimen until off-treatment criteria are met, provided they meet re-treatment eligibility requirements. Please note that progressive disease must be confirmed within not less than 4 weeks.

Re-induction is not allowed in patients who experience

- At least one Grade 3 or higher gastrointestinal immune-related adverse events (irAE) including, but not limited to, ≥ Grade 3 colitis or diarrhea **OR**
- Uveitis of any grade **OR**
- At least one certain other immune-related adverse events (irAE) (refer to section 5.2 and 5.3) **OR**
- Disease progression following the first cycle of study medication

Exemption: Re-induction is allowed in patients who experience selected irAEs related to ipilimumab that have completely resolved with immunosuppressive therapy or are adequately controlled with hormone therapy, may be considered for further re-treatment with ipilimumab under this study, at the time of disease progression. The list of completely reversible or medically managed immune-related adverse events (irAEs) **eligible** for consideration is:

- Reversible autoimmune hepatitis
- Medically managed endocrinopathy
- Reversible dermatological toxicity

All other irAEs are not considered eligible for re-treatment!

Appendix 9: RECIST Criteria

Evaluation of measurable lesions

- Sum of all diameters

...... Comparisons with

CR	Disappearance of all measurable lesions<br>No new lesions	
PR	≥ 30% decrease in the LD sum	Baseline
PD	≥ 20% increase in the LD sum or at least one new lesion	Minimum = smallest LD sum subsequent to therapy start
SD	Beyond the conditions cited above	Minimum

Evaluation of non measurable lesions

CR	Disappearance of all non measurable lesions<br>No new lesions
Incomplete Response/ SD	Persistence of non measurable lesions
PD	New lesions<br>Progress of non measurable lesions
SD	Tumor marker

Best Overall Response

- Best response during therapy prior to PD

Target lesion	Non target lesion	OR
CR	CR	CR
CR	SD / IR	PR
PR	No PD	PR
SD	No PD	SD
PD	–	PD
–	PD	PD
–	New lesion	PD
New lesion	-	PD

Appendix 10-15: Management Algorithm for AEs

7.3 Suggested Evaluation and Treatment for Immunerelated Adverse Events

Early diagnosis and treatment intervention for high-grade irAEs can help prevent the occurrence of complications, such as GI perforation. Gastrointestinal (diarrhea) and skin (rash)-related toxicities are the most common irAEs reported in studies with ipilimumab. Suggested evaluation procedures for suspected irAEs of the GI tract, liver, skin, eye, pituitary, and adrenal gland are described below. When symptomatic therapy is inadequate or inappropriate, an irAE should be treated with systemic corticosteroids followed by a gradual taper.

Infrequently, subjects with irAEs may require courses of corticosteroid or other immunosuppressive agents that exceed 4 weeks in duration. It is recommended that subjects receiving immunosuppressive agents beyond 4 to 6 weeks should also receive antimicrobial prophylaxis to protect against the emergence of opportunistic infections.
Such prophylaxis should include protection against *Pneumocystis jiroveci* (formerly *P carinii*) and prevalent fungal strains, as well as considerations for any additional pathogens that may be indicated by the medical history (e.g., herpes simplex virus, cytomegalovirus) or the environment (e.g., occupation, recent travel) of the subject. Consultation with infectious disease specialists may be considered.
Immune-related (ir)AE management algorithms for general irAEs, and ipilimumabrelated diarrhea, hepatotoxicity, endocrinopathy, and neuropathy have been developed and are attached in Appendices 6, 7, 8, 9, and 10 respectively.

7.3.1 Gastrointestinal Tract

The differential diagnosis for subjects presenting with abdominal pain should include colitis, perforation, or pancreatitis. Additionally, a few subjects with abdominal pain also
had acute swelling of the cecal wall on CT scan that may have represented localized inflammation.
Diarrhea (defined as either first watery stool, or increase in frequency 50% above baseline with urgency or nocturnal bowel movement, or bloody stool) should be further
evaluated and infectious or alternate etiologies ruled out. Subjects should be advised to
inform the investigator if any diarrhea occurs, even if it is mild. An algorithm for managing subjects with diarrhea or suspected colitis is provided in Appendix 7.
The majority of subjects with ipilimumab-induced diarrhea or colitis responded to symptomatic therapy or corticosteroids. Permanent discontinuation of ipilimumab and starting high dose corticosteroid therapy (e.g., methylprednisolone 2 mg/kg once or twice per day or equivalent) is strongly recommended for ipilimumab
related ≥ Grade 3 diarrhea/colitis and steroids should be slowly tapered according to symptomatic response over at least 1 month.

Subjects with ipilimumab-related Grade 2 diarrhea/colitis have to skip ipilimumab dosing
and may be initially treated conservatively with loperamide, fluid replacement or budesonide, but should be immediately switched to corticosteroids (prednisone 1 mg/kg or equivalent) if symptoms persist for 3-5 days or worsen. Most subjects with diarrhea/colitis will rapidly respond to initiation of corticosteroids. The dose should be

gradually tapered over at least a 1-month duration. Lower doses of prednisone may be considered for less severe cases of colitis. It is suggested that prednisone (for oral administration) or methylprednisolone (for IV administration) be the corticosteroids of choice in the treatment of colitis. Caution should be taken in the use of narcotics in patients with abdominal pain or colitis/diarrhea as narcotic use may mask the signs of colonic perforation.

Infrequently, subjects will appear refractory to corticosteroids or will flare following taper of corticosteroids. In these subjects, unless contraindicated (i.e., sepsis, perforation and other serious infections), a single dose of infliximab at 5 mg/kg may provide benefit.

For patients with concomitant hepatitis, use of mycophenolate mofetil is recommended in place of infliximab. For patients with long term immunosuppressive therapy, administer antimicrobial prophylactics as appropriate per institutional guidelines. Such cases should be discussed with the sponsor study medical monitor.

If the event is prolonged or severe or is associated with signs of systemic inflammation oracute phase reactants (e.g., increased CRP or platelet count; or bandemia), it is recommended that sigmoidoscopy (or colonoscopy, if appropriate) with colonic biopsy of 3 to 5 specimens for standard paraffin block be performed. If possible, 1 to 2 biopsy specimens should be snap-frozen and stored. All subjects with confirmed colitis should also have an ophthalmologic examination, including a slit-lamp exam, to rule out uveitis. Tests should also be performed for stool calprotectin and WBCs as outlined in Appendix 7.

7.3.2 Liver

Liver function tests should always be performed and reviewed prior to administration of all ipilimumab doses. Patients treated with ipilimumab may develop elevation in LFT in
the absence of clinical symptoms. In addition, subjects presenting with right upper quadrant abdominal pain, unexplained nausea, or vomiting should have LFTs performed immediately and reviewed before administering the next dose of study drug. Any increase in LFT should be evaluated to rule out non-inflammatory causes of hepatotoxicity including infections, disease progression or medications and followed with frequent LFT monitoring at 3-day intervals until resolution.

Any LFTs ≥ Grade 2 (for subjects with normal baseline LFT) or LFT ≥ 2 times baseline values (for subjects with baseline LFT of Grade 1 or 2) should prompt treating physicians to: (1) contact the medical monitor; (2) increase frequency of monitoring LFTs to at least every 3 days until LFT have stabilized or improved; (3) investigate to rule out non-irAE etiologies; and (4) initiate an autoimmunity evaluation. Disease progression, other malignancies, concurrent medications, viral hepatitis, and toxic etiologies should be considered and addressed, as appropriate. Imaging of the liver, gall bladder, and bile ducts should be considered to rule out neoplastic or other non-irAE-related causes for the increased LFTs. An ANA, perinuclear anti-neutrophil cytoplasmic antibody (pANCA), and anti-smooth muscle antibody test should be performed if an autoimmune etiology is considered.

If the LFTs are > 5 times the upper limit of normal (ULN) or total bilirubin is > 3 times the ULN, then ipilimumab dosing should be held according to the dose modification guidelines.

Subjects with LFT elevations > 8x the ULN that are judged to be due to ipilimumab should initiate high-dose corticosteroid therapy (e.g., methylprednisolone 2 mg/kg once or twice daily or equivalent) and permanently stop administration of ipilimumab. In subjects with > 8x ULN, LFT should be performed daily until stable or declining for

5 consecutive days. LFT should be monitored for at least 2 consecutive weeks to ensure sustained treatment response. If symptoms or LFT elevations are controlled, the corticosteroid dose should be gradually tapered over a period of at least 1 month. Flare in LFTs during this taper may be treated with an increase in the dose of steroid and a slower taper.

In subjects without response to corticosteroid therapy within 3 to 5 days or who have an LFT flare during steroid tapering that is not responsive to an increase in steroids, addition of immunosuppression with mycophenolate mofetil should be considered after a gastroenterology/hepatology consult. Patients receiving immunosuppression for more than 4 weeks should be evaluated for prophylaxis of opportunistic infections per institutional guidelines. An algorithm for evaluating subjects with elevated LFT is provided in Appendix 8.

7.3.3 Skin

A dermatologist should evaluate persistent or severe rash or pruritus. A biopsy should be performed if appropriate and if possible, photos of the rash should also be obtained.

Patients with low-grade ipilimumab-mediated skin toxicity (Grade 1 or 2) may remain on therapy and could be treated with symptomatic therapy (e.g., antihistamines). Low-grade symptoms persisting for 1 to 2 weeks and relapsing should be treated with topical or oral corticosteroid therapy (e.g., prednisone 1 mg/kg once daily or equivalent). High-grade (Grade 3 or 4) symptoms require high-dose IV corticosteroid therapy (e.g., methylprednisolone 2 mg/kg once or twice per day or equivalent) to control initial symptoms. A skin biopsy should be performed if appropriate. Once rash or pruritis is controlled, the initiation of corticosteroid taper should be based on clinical judgment; however, the corticosteroid dose should be gradually tapered over a period of at least 1 month.

Patients with any high-grade skin related toxicity (Grade 3 regardless of causality) have to skip ipilimumab and may only continue treatment with ipilimumab if the initial symptoms have improved to ≤ Grade 1, while patients with grade 4 skin toxicities have to permanently discontinue ipilimumab.

7.3.4 Endocrine

Most subjects with hypopituitarism presented with nonspecific complaints such as fatigue, visual field defects, confusion, or impotence. Some have had headache as the predominant presentation. The majority of subjects with hypopituitarism demonstrated enlarged pituitary glands based on brain MRI. Low adrenocorticotropic hormone (ACTH) and cortisol were the most common biochemical abnormality; low TSH, free T4, T3, testosterone or prolactin have also been reported in some subjects. Subjects with unexplained symptoms such as fatigue, myalgias, impotence, mental status changes, or constipation should be investigated for the presence of thyroid, pituitary or adrenal endocrinopathies. An endocrinologist should be consulted if an endocrinopathy is suspected. If there are any signs of adrenal crisis such as severe dehydration, hypotension, or shock, IV corticosteroids with mineralocorticoid activity (e.g., methylprednisolone) should be initiated immediately. If the patient's symptoms are suggestive of an endocrinopathy but the patient is not in adrenal crisis, an endocrine laboratory results should be evaluated before corticosteroid therapy is initiated. Endocrine work up should include at least thyroid stimulating hormone (TSH) and free T4 levels should be obtained to determine if thyroid abnormalities are present. TSH, prolactin and a morning cortisol level will help to differentiate primary adrenal insufficiency from primary pituitary insufficiency. Radiographic imaging (e.g.,

MRI) with pituitary cuts should be performed. If the pituitary scan and/or endocrine laboratorytests are abnormal, a short course of high dose corticosteroids (e.g., dexamethasone 4 mg every 6 hours or equivalent) should be considered in an attempt to treat the presumed pituitary inflammation, but it is currently unknown if this will reverse the pituitary dysfunction. Abrupt discontinuation of corticosteroids should be avoided due to possible prolonged adrenal suppression. Once symptoms or laboratory abnormalities are controlled, and overall patient improvement is evident, the initiation of steroid taper should be based on clinical judgment; however the corticosteroid dose should be gradually tapered over a period of at least 1 month.
Appropriate hormone replacement therapy should be instituted if an endocrinopathy isdocumented, and it is possible that subjects may require life-long hormone replacement. An endocrinopathy management algorithm is presented in Appendix 9.

7.3.5 Neuropathy

Subjects presenting with sensory symptoms lasting more than 5 days or motor symptoms confirmed by physical examinations should be evaluated and non-inflammatory causes such as disease progression, infections (including Lyme disease), metabolic syndromes and medications (such as taxanes and/or platinum salts) should be ruled out. A neurology consult should be obtained and diagnostic characterization of the neurological syndrome (electromyogram, nerve conduction studies) should be started. The next dose of ipilimumab should be skipped if the event of a Grade 2 neuropathy (sensitive or motor) is related to study drug. The administration of ipilimumab should be permanently discontinued in patients with Grade 3 or 4 sensory neuropathy suspected to be related to study drug. Patients should be treated according to institutional guidelines and the administration of IV steroids (e.g., methylprednisolone 2 mg/kg once or twice per day or equivalent) should be considered. The administration of ipilimumab should be permanently discontinued in patients with Grade 3 or 4 motor neuropathy regardless of the causality to study drug. Patients with Grade 3 or 4 motor neuropathy who are clinically stable should be treated according to institution guidelines and the administration of IV steroids therapy (e.g., methylprednisolone 2 mg/kg once or twice per day or equivalent) should be considered. Patients with Grade 3 or 4 motor neuropathy who are not clinically stable or who have atypical symptoms should be hospitalized and the IV administration of steroids should be initiated and IV immunoglobulin (IVIg) or other immunosuppressive therapies (as appropriate) should be considered. A neuropathy management algorithm is presented in Appendix 10.

7.3.6 Other

Ocular inflammation (episcleritis or uveitis) was reported in a few subjects. These conditions responded to topical corticosteroid therapy. An ophthalmologist should evaluate visual complaints with examination of the conjunctiva, anterior and posterior chambers, and retina. Visual field testing and an electroretinogram should also be performed. Examination and testing should be documented on the appropriate case report form (CRF). Ipilimumab-related uveitis or episcleritis may be treated with topical corticosteroid eye drops. Symptoms of abdominal pain associated with elevations of amylase and lipase suggestive of pancreatitis may be associated with anti-CTLA-4 monoclonal antibody administration.
The differential diagnosis of acute abdominal pain should include pancreatitis.
Appropriate evaluation should include serum amylase and lipase tests.

Diarrhea Management Algorithm

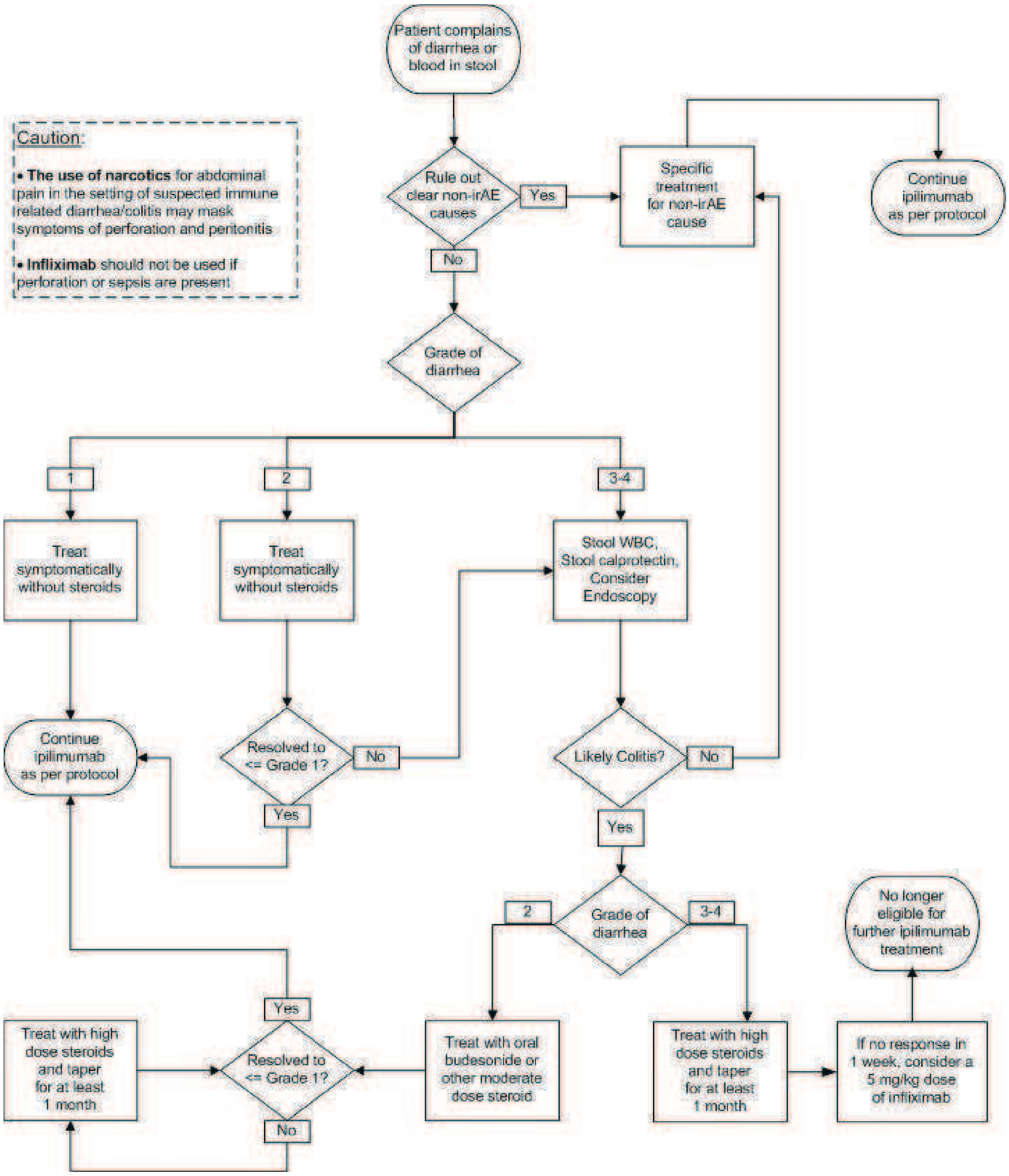

Diarrhea	**GRADE 1**<br>Increase of < 4 stools per day over baseline; mild increase in ostomy output compared with baseline	**GRADE 2**<br>Increase of 4-6 stools per day over baseline; IV fluids indicated < 24 hrs; moderate increase in ostomy output compared to baseline; not interfering with ADL	**GRADE 3**<br>Increase of ≥ 7 stools per day over baseline; incon-tinence; IV fluids ≥ 24 hrs; hospitalization; severe increase in ostomy output compared to baseline; interfering with ADL	**GRADE 4**<br>Life-threatening consequences (eg, hemodynamic collapse)	**GRADE 5**<br>Death

Approved v 6.0 930017531 5.0

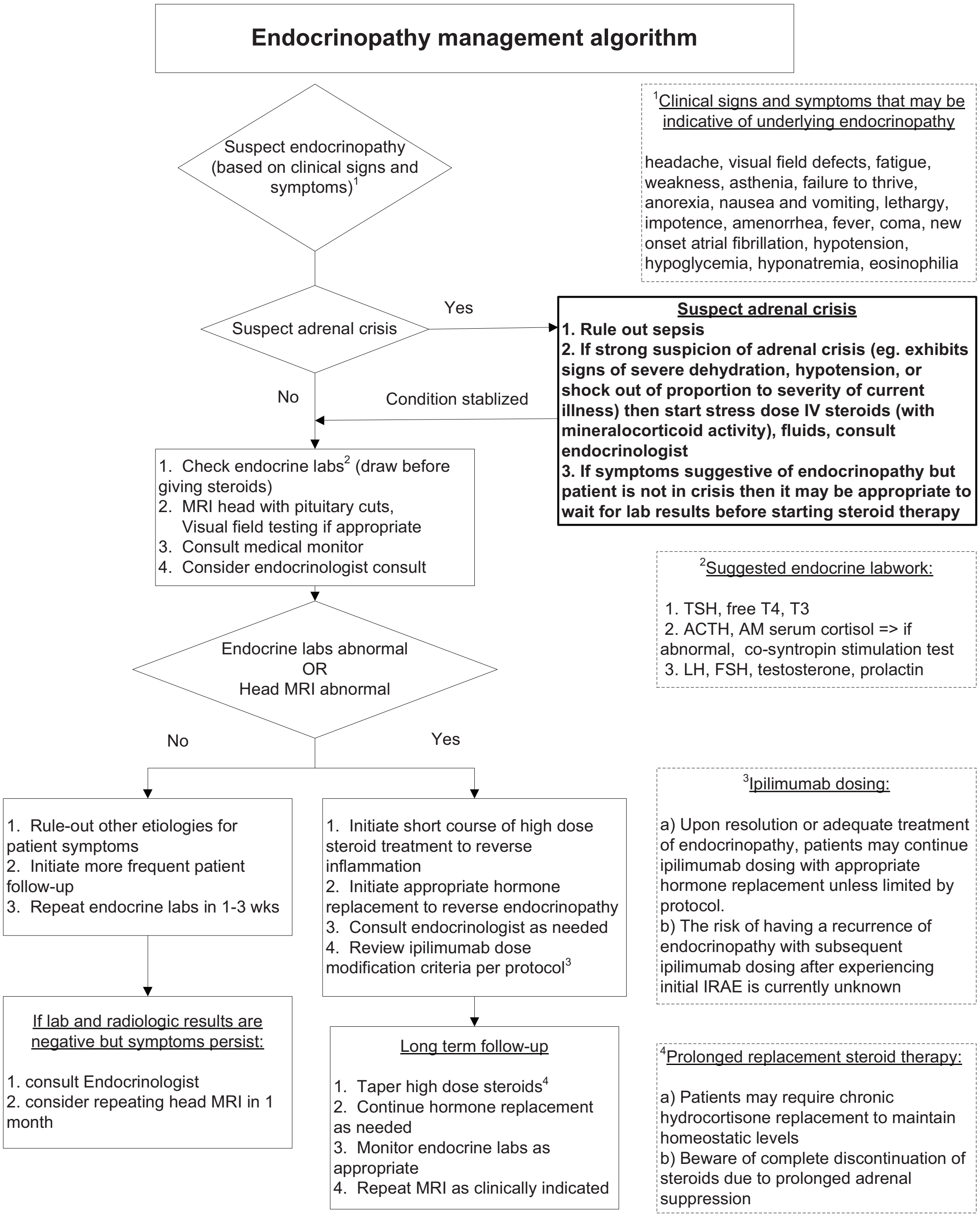

Footnote
For numbered footnotes ([1,2,3,4]), please refer to further explanation and text found in the corresponding dotted line boxes to the right side of the algorithm

Approved v 6.0 930017531 5.0

The most current experience with immune-related hepatitis has allowed further development of this management algorithm to include recommendations for treatment.

HEPATOTOXICITY THERAPEUTIC INTERVENTION ALGORITHM

Situation: rising liver function tests (LFTs) > 8X ULN or suspected immune-mediated hepatitis

1) Admit subject to hospital for evaluation and close monitoring
2) Stop further ipilimumab dosing until hepatotoxicity is resolved. Consider permanent discontinuation of ipilimumab per protocol (Section 6.2.5 of protocol)
3) Start at least 120 mg methylprednisolone sodium succinate per day, given IV as a single or divided dose
4) Check liver laboratory test values (LFTs, T-bilirubin) daily until stable or showing signs of improvement for at least 3 consecutive days
5) If no decrease in LFTs after 3 days or rebound hepatitis occurs despite treatment with corticosteroids, then add mycophenolate mofetil 1g BID per institutional guidelines for immunosuppression of liver transplants (supportive treatment as required, including prophylaxis for opportunistic infections per institutional guidelines)
6) If no improvement after 5 to 7 days, consider adding 0.10 to 0.15 mg/kg/day of tacrolimus (trough level 5-20 ng/mL)
7) If target trough level is achieved with tacrolimus but no improvement is observed after 5 to 7 days, consider infliximab, 5 mg/kg, once
8) Continue to check LFTs daily for at least 2 weeks to monitor sustained response to treatment

A flow chart of the algorithm is depicted in the following page.

Approved v 6.0 930017531 5.0

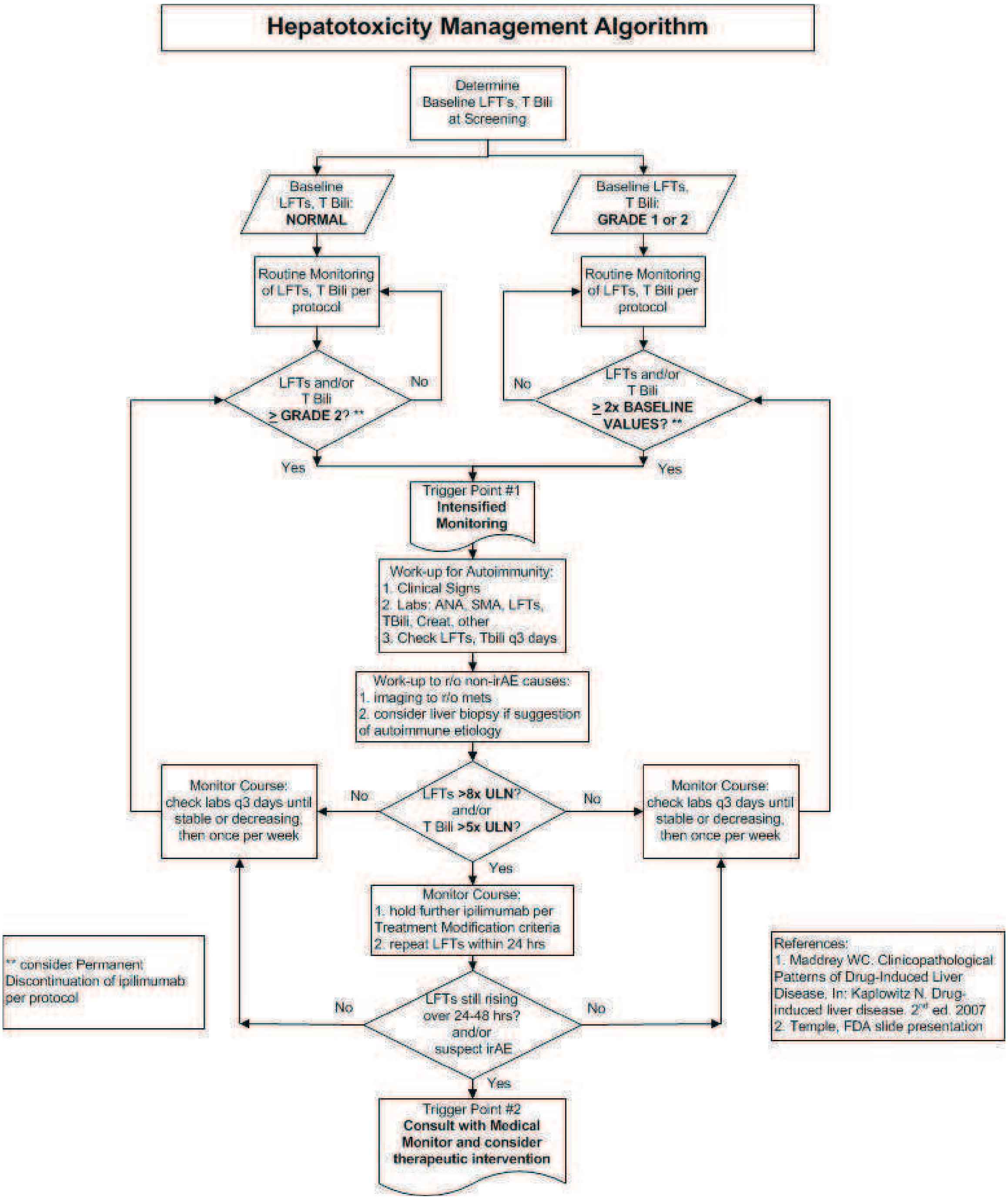
Hepatotoxicity Management Algorithm
Determine Baseline LFT's, T Bili at Screening
Baseline LFTs, T Bili: NORMAL
Baseline LFTs, T Bili: GRADE 1 or 2
Routine Monitoring of LFTs, T Bili per protocol
Routine Monitoring of LFTs, T Bili per protocol
LFTs and/or T Bili ≥ GRADE 2? **
No
No
LFTs and/or T Bili ≥ 2x BASELINE VALUES? **
Yes
Yes
Trigger Point #1 Intensified Monitoring
Work-up for Autoimmunity: 1. Clinical Signs 2. Labs: ANA, SMA, LFTs, TBili, Creat, other 3. Check LFTs, Tbili q3 days
Work-up to r/o non-irAE causes: 1. imaging to r/o mets 2. consider liver biopsy if suggestion of autoimmune etiology
Monitor Course: check labs q3 days until stable or decreasing, then once per week
No
LFTs >8x ULN? and/or T Bili >5x ULN?
No
Monitor Course: check labs q3 days until stable or decreasing, then once per week
Yes
Monitor Course: 1. hold further ipilimumab per Treatment Modification criteria 2. repeat LFTs within 24 hrs
** consider Permanent Discontinuation of ipilimumab per protocol
No
LFTs still rising over 24-48 hrs? and/or suspect irAE
No
References: 1. Maddrey WC. Clinicopathological Patterns of Drug-Induced Liver Disease. In: Kaplowitz N. Drug-induced liver disease. 2nd ed. 2007 2. Temple, FDA slide presentation
Yes
Trigger Point #2 Consult with Medical Monitor and consider therapeutic intervention

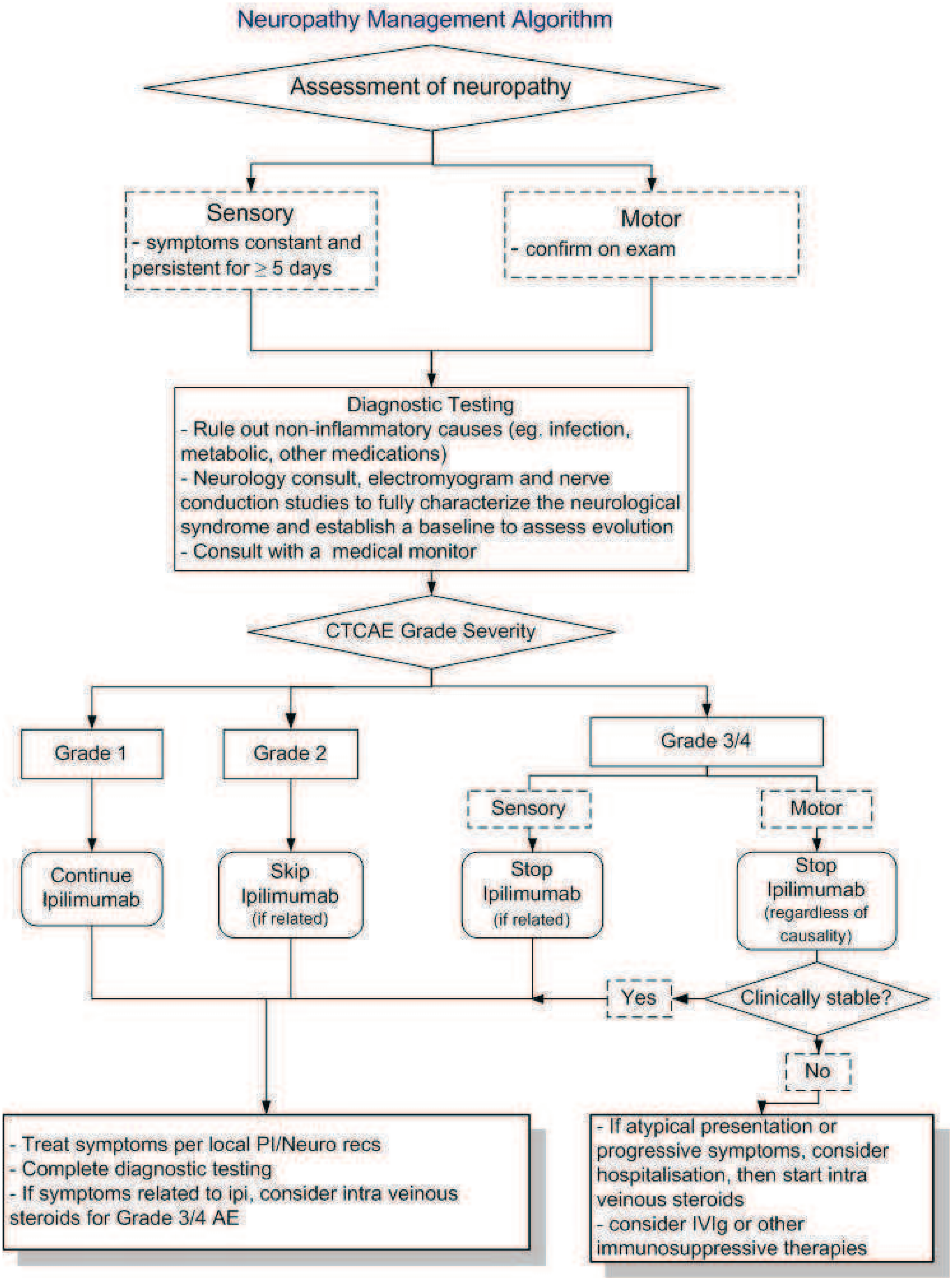
Neuropathy Management Algorithm
Assessment of neuropathy
Sensory
- symptoms constant and persistent for ≥ 5 days
Motor
- confirm on exam
Diagnostic Testing
- Rule out non-inflammatory causes (eg. infection, metabolic, other medications)
- Neurology consult, electromyogram and nerve conduction studies to fully characterize the neurological syndrome and establish a baseline to assess evolution
- Consult with a medical monitor
CTCAE Grade Severity
Grade 1
Grade 2
Grade 3/4
Sensory
Motor
Continue Ipilimumab
Skip Ipilimumab (if related)
Stop Ipilimumab (if related)
Stop Ipilimumab (regardless of causality)
Yes
Clinically stable?
No
- Treat symptoms per local PI/Neuro recs
- Complete diagnostic testing
- If symptoms related to ipi, consider intra veinous steroids for Grade 3/4 AE
- If atypical presentation or progressive symptoms, consider hospitalisation, then start intra veinous steroids
- consider IVIg or other immunosuppressive therapies

REFERENCES

Allison J.P., et al., Manipulation of costimulatory signals to enhance antitumor t cell responses. Curr Opin Immunol, 1995. 7(5):682-686.

Asim Amin A, et. al. Systemic Corticosteroids for the Treatment of Immune-Related Adverse Events (irAEs) Do Not Impact the Development or Maintenance of Ipilimumab Clinical Activity. [Poster presentation: #9037]. Presented at the 2009 American Society of Clinical Oncology Annual Meeting; May 29-June 2, 2009. Orlando, FL, USA.

Balch et al., Final Version of 2009 AJCC Melanoma Staging and Classification J Clin Oncol 2009 doi/10.1200/JCO.2009.23.4799

Becker JC et al. Treatment of disseminated ocular melanoma with sequential fotemustine, interferon alpha, and interleukin 2. Brit J Cancer 2002 Oct 7;87(8):840-5.

Bedekian AY, Millward, M, Pehamberger H, Conry R, Gore M, Trefzer U, Pavlick AC, et al. Bcl-2 Antisense (oblimersen sodium) Plus Dacarbazine in Patients With Advanced Melanoma: the Olimersen Melanoma Study. J Clin Oncol. 2006:24:4738 4745.

Blansfield JA, et al. Cytotoxic Tlymphocyte-associate antigen-4 blockage can induce autoimmune hypophysitis in patients with metastatic melanoma and renal cancer. J Immunother 2005;28(6): 593-598.

CA184022 Clinical Study Report. A Randomized, Double-Blind, Multi-center, Phase II Fixed Dose Study of Multiple Doses of Ipilimumab (MDX-010) Monotherapy in Patients with Previously Treated Unresectable Stage III or IV Melanoma. Bristol-Myers Squibb Company and Medarex Inc, 2008. Document Control No. 930026869.

CA184008 Clinical Study Report. A Multicenter, Single Arm Phase 2 Study of MDX-010 (BMS-734016) Monotherapy in Patients with Previously Treated Unresectable Stage III or IV Melanoma. Bristol-Myers Squibb Company and Medarex Inc, 2008. Document Control No. 930025464.

CA184007 Clinical Study Report. Randomized, Double-Blind, Placebo-Controlled, Phase 2 Study Comparing the Safety of Ipilimumab Administered With or Without Prophylactic Oral Budesonide (Entocort™ EC) in Patients with Unresectable Stage III or IV Malignant Melanoma. Bristol-Myers Squibb Company and Medarex Inc, 2008. Document Control No. 930026874.

CA184004 Clinical Study Report. An Exploratory Study to Determine Potential Predictive Markers of Response and/or Toxicity in Patients with Unresectable Stage III or IV Malignant Melanoma Randomized and Treated with Ipilmumab (MDX-010/BMS-734016) at Two Dose Levels. Bristol-Myers Squibb Company and Medarex Inc, 2008. Document Control No. 930027670.

Chambers C.A., et al., Lymphoproliferation in CTLA-4-deficient mice is mediated by costimulation-dependent activation of CD4+ T cells. Immunity, 1997, 7(6): 885-895.

Chapman PB, Einhorn E, Meyers ML, Saxman S, Destro AN, Panageas KS, Begg CB, et al. Phase III Multicenter Randomized Trial of the Dartmouth Regimen Versus Dacarbazine in Patients with Metastatic Melanoma. J Clin Oncol. 1999;17(9)2745-2751.

Chen L.S., et al., Costimulation of antitumor immunity by the B7 counterreceptor for the T lymphocyte molecules CD28 and CTLA-4. Cell, 1992. 71(7):1093-1102.

Hodi FS et al. Improved Survival with Ipilimumab in Patients with Metastatic Melanoma. *N Engl J Med.* 2010 Jun 14. [Epub ahead of print]

Hodi FS et al. Re-induction with ipilimumab, gp100 peptide vaccine, or a combination of both from a phase III, randomized, double-blind, multicenter study of previously treated patients with unresectable stage III or IV melanoma. J Clin Oncol 28:7s, 2010 (suppl; abstr 8509)

Jaber SH, et al. Skin reactions in a subset of patients with stage IV melanoma treated with anti-cytotoxic Tlymphocyte antigen 4 monoclonal antibody as a single agent. Arch Dermatol 2006;142:166-172.

Kearney E.R., et al., Antigen-dependent clonal expansion of a trace population of antigen-specific CD4+ T cells in vivo is dependent on CD28 costimulation and inhibited by CTLA-4. J Immunol, 1995. 155(3): 1032-1036.

Korn EL, Liu P-Y, Lee S, et al. Meta-analysis of phase II cooperative group trials in metastatic stage IV melanoma to determine progression-free and overall survival benchmarks for future phase II trials. J Clin Oncol. 2008;26:527-534.

Krummel M.F. and Allison J.P. CD28 and CTLA-4 have opposing effects on the response of T cells to stimulation. J Exp Med 1995; 182(2): 459-465.

Krummel M.F., et al., Superantigen responses and co-stimulation: CD28 and CTLA-4 have opposing effects on T cell expansion in vitro and in vivo. Int Immunol, 1996. 8(4): 519-523.

Ku GY, et al. Single-institution experience with ipilimumab in advanced melanoma patients in the compassionate use setting: lymphocyte count after 2 doses correlates with survival. Cancer. 2010 Apr 1;116(7):1767-75.

Lenschow D.J., et al., CD28/B7 system of T cell costimulation. Ann Rev Immunol, 1996. 14:233-258.

Linsley P.S., et al., CTLA-4 is a second receptor for the B cell activation antigen B7. J Exp Med, 1991. 174(3):561-569.

MDX010-19 Clinical Study Report. A Study of Intra-patient Escalating Doses of MDX-010 Given Alone or in Combination With Two GP100 Peptides Emulsified with Montanide ISA-51 in the Treatment of Patients with Stage IV Melanoma. Medarex Inc. and Bristol-Myers Squibb Company, 2007. Document Control No. 930023186.

Middleton MR, Grob JJ, Aaronson N, Fierlbeck G, Tilgen W, Seiter S, Gore M, et al. Randomized Phase III Study of Temozolomide Versus Dacarbazine in the Treatment of Patients With Advanced Metastatic Malignant Melanoma. J Clin Oncol. 2000;18: 158-166.

O'Day SJ, et al. Ann Oncol. Efficacy and safety of ipilimumab monotherapy in patients with pretreated advanced melanoma: a multicenter single-arm phase II study. 2010 Feb 10. epub

O´Day SJ et al. A phase III, randomized, double-blind, multicenter study comparing monotherapy with ipilimumab or gp100 peptide vaccine and the combination in patients with previously treated, unresectable stage III or IV melanoma. J Clin Oncol 28:18s, 2010 (suppl; abstr 4)

Penel N *et al.* O-Mel-Inib: a Cancero-pole Nord-Ouest multicenter phase II trial of high-dose imatinib mesylate in metastatic uveal melanoma. Invest New Drugs 2008;26: 561-5.

Robinson MR, et al. Cytotoxic Tlymphocyte-associate antigen-4 blockage in patients with metastatic melanoma: A new cause of uveitis. J Immunother 2004;27(6):478-479.

Schadendorf D et al Dose-intensified bi-weekly temozolomide in patients with asymptomatic brain metastases from malignant melanoma: a phase II DeCOG/ADO study Ann Oncol 17: 1592–1597, 2006

Schwartz R.H. Costimulation of T lymphoctyes: the role of CD28, CTLA4, and B7/BB1 in interleukin-2 production and immunotherapy. Cell, 1992. 71(7): 1065-1068.

Thompson C.B., and Allison J.P. The emerging role of CTLA-4 as an immune attenuator. Immunity, 1997. 7(4): p.445-450.

Tivol E.A., et al., Loss of CTLA-4 leads to massive lymphoproliferation and fatal multiorgan tissue destruction, revealing a critical negative regulatory role of CTLA-4. Immunity, 1995. 3(5): 541-547.

Townsend S.E., et al., Tumor rejection after direct costimulation of CD8+ T cells by B7-transfected melanoma cells. Science, 1993. 259(5093):368-370.

Townsend S.E., et al., Specificity and longevity of antitumor immune responses induced by B7-transfected tumors. Cancer Res, 1994. 54(24):6477-6483.

Walunas T.L., et al., CTLA-4 can function as a negative regulator of T cell activation. Immunity, 1994. 1(5): p. 405-413.

Waterhouse P. et al., Lymphoproliferative disorders with early lethality in mice deficient in CTLA-4. Science, 1995. 270(5238): 985-988.

Wolchok JD, Hoos A, O'Day S, Weber JS, Hamid O, Lebbé C, Maio M, Binder M, Bohnsack O, Nichol G, Humphrey R, Hodi FS. Guidelines for the Evaluation of Immune Therapy Activity in Solid Tumors: Immune-Related Response Criteria. Clin Cancer Res 2009;15(23):7412-7420.

Wolchok J, et al. Ipilimumab monotherapy in patients with pretreated, advanced melanoma: a randomized, double-blind, multicenter, phase II dose-ranging study. *Lancet Oncol* 2010 Feb;11(2):155-64. Epub 2009 Dec 8.

Yang A et al. CTLA-4 blockade with ipilimumab increases peripheral CD8+ T cells: Correlation with clinical outcomes J Clin Oncol 28:7s, 2010 (suppl; abstr 2555)
