# Supplementary material for: Phase II DeCOG-Study of Ipilimumab in Pretreated and Treatment-Naïve Patients with Metastatic Uveal Melanoma
Source: PLoS One. 2015 Mar 11;10(3):e0118564. doi: 10.1371/journal.pone.0118564 (PMC4356548; doi:10.1371/journal.pone.0118564)
Supplement: S1 Table — (PDF) [file pone.0118564.s003.pdf]

Table S1: Summary of previous studies reporting efficacy of ipilimumab and other therapies in metastatic uveal melanoma within the last 5 years.

Author	Year	Drug	Study design	Patients with uveal melanoma (n)	Treatment (pretreated vs. treatment-naïve)	Median PFS (months)	Median OS (months)	6-month PFS (%)	1-year OS (%)	2-year OS (%)
Kelderman	2013	Ipilimumab 3mg	EAP	22	pretreated	2.9	5.2	n.a.	27	n.a.
Khattak	2013	Ipilimumab 3mg	EAP	5	pretreated	n.a.	10.3	n.a.	n.a.	n.a.
Danielli	2012	Ipilimumab 10mg	EAP	13	pretreated	n.a.	9.0	n.a.	n.a.	n.a.
Maio	2013	Ipilimumab 3mg	EAP	83	pretreated	3.6	6.0	n.a., 1-year PFS: 11	Estimated 31	n.a.
Luke	2013	Ipilimumab 3mg and 10 mg	Retro-spective	39 (34: 3 mg; 5: 10 mg)	treatment-naïve (n=4) and pretreated	n.a.	9.6	n.a.	n.a.	n.a.
Deo	2014	Ipilimumab 3mg	Retro-spective	24	pretreated	2.8	9.7	n.a.	46	11
Piulats	2014	Ipilimumab 10mg	Phase 2	32	treatment-naive	n.a.	9.8	27	n.a.	n.a.
Zimmer	2014	Ipilumumab 3mg	Phase 2	53	treatment-naïve (n=8), pretreated (n=45)	2.8	6.8	19	22	7

Author	Year	Drug	Study design	Patients with uveal melanoma (n)	Treatment (pretreated vs. treatment-naïve)	Median PFS (months)	Median OS (months)	6-month PFS (%)	1-year OS (%)	2-year OS (%)
Carvajal	2014	1.dacarbazine 1000 mg/qm or temozolamide 150mg/qm<br>2. selumetinib 75 mg twice daily	Randomized open-label, phase 2	Total:101<br>Chemo-therapy:51<br>Selumetinib:50	treatment-naïve and pretreated	Chemotherapy: 7 weeks<br>Selumetinib: 15.9 weeks	Chemotherapy: 9.1<br>Selumetinib: 11.8	5.7<br>22.9	Around 32<br>Around 42	n.a.
Bhatia	2012	Carboplatin (AUC=6)+paclitaxel (225 mg/qm) plus sorafenib (400 mg per os twice daily)	Phase 2	25	treatment-naïve (n=20), pretreated (n=4)	4.0	11.0	29	Estimated 42	n.a.
Leyvraz	2014	1.hepatic intra-arterial (HIA) fotemustine 100 mg/qm<br>2.intravenous (IV) fotemustine 100mg/qm	Randomized phase 3	171	treatment-naïve	HIA: 4.5<br>IV: 3.5	HIA: 14.6<br>IV: 13.8	n.a.	n.a.	n.a.
Spagnolo	2013	Fotemustine 100mg/qm intravenous	Retro-spective	25	treatment-naive	n.a.	13.9	n.a.	60	n.a.
Homsi	2010	DHA-paclitaxel 500mg/qm	Phase 2	22	treatment-naïve (n=11), pretreated (n=11)	n.a.	9.8	n.a.	50	<10

Abbreviations: EAP, expanded access program; AUC, area under the curve; DHA, docosahexaenoic-acid; PFS, progression-free survival; OS, overall survival; n.a., not applicable
